# Radiation immunodynamics in patients with glioblastoma receiving chemoradiation

**DOI:** 10.3389/fimmu.2024.1438044

**Published:** 2024-09-13

**Authors:** Lindsey Sloan, Rupashree Sen, Chunnan Liu, Michele Doucet, Lee Blosser, Lisa Katulis, David O. Kamson, Stuart Grossman, Matthias Holdhoff, Kristin J. Redmond, Harry Quon, Michael Lim, Charles Eberhart, Drew M. Pardoll, Chen Hu, Sudipto Ganguly, Lawrence R. Kleinberg

**Affiliations:** ^1^ Department of Radiation Oncology, University of Minnesota, Minneapolis, MN, United States; ^2^ University of Minnesota Medical School, University of Minnesota, Minneapolis, MN, United States; ^3^ Masonic Cancer Center, University of Minnesota, Minneapolis, MN, United States; ^4^ Department of Radiation Oncology and Molecular Radiation Sciences, Johns Hopkins University School of Medicine, Baltimore, MD, United States; ^5^ The Sidney Kimmel Comprehensive Cancer Center, Johns Hopkins University School of Medicine, Baltimore, MD, United States; ^6^ The Bloomberg-Kimmel Institute for Cancer Immunotherapy, Johns Hopkins University School of Medicine, Baltimore, MD, United States; ^7^ Division of Biostatistics and Bioinformatics, Sidney Kimmel Comprehensive Cancer Center, Johns Hopkins University School of Medicine, Baltimore, MD, United States; ^8^ Brain Cancer Research Program, Johns Hopkins University School of Medicine, Baltimore, MD, United States; ^9^ Department of Neurology, Johns Hopkins University School of Medicine, Baltimore, MD, United States; ^10^ Department of Neurosurgery, Stanford University School of Medicine, Stanford, CA, United States; ^11^ Department of Pathology, Johns Hopkins University School of Medicine, Baltimore, MD, United States; ^12^ Department of Biostatistics, Johns Hopkins University, Baltimore, MD, United States

**Keywords:** glioblastoma, immune system, chemoradiotherapy, radiotherapy, brain tumor

## Abstract

**Introduction:**

This is a prospective, rigorous inquiry into the systemic immune effects of standard adjuvant chemoradiotherapy, for WHO grade 4, glioblastoma. The purpose is to identify peripheral immunologic effects never yet reported in key immune populations, including myeloid-derived suppressor cells, which are critical to the immune suppressive environment of glioblastoma. We hypothesize that harmful immune-supportive white blood cells, myeloid derived suppressor cells, expand in response to conventionally fractionated radiotherapy with concurrent temozolomide, essentially promoting systemic immunity similar what is seen in chronic diseases like diabetes and heart disease.

**Methods:**

16 patients were enrolled in a single-institution, observational, immune surveillance study where peripheral blood was collected and interrogated by flow cytometry and RNAseq. Tumor tissue from baseline assessment was analyzed with spatial proteomics to link peripheral blood findings to baseline tissue characteristics.

**Results:**

We identified an increase in myeloid-derived suppressor cells during the final week of a six-week treatment of chemoradiotherapy in peripheral blood of patients that were not alive at two years after diagnosis compared to those who were living. This was also associated with a decrease in CD8^+^ T lymphocytes that produced IFNγ, the potent anti-tumor cytokine.

**Discussion:**

These data suggest that, as in chronic inflammatory disease, systemic immunity is impaired following delivery of adjuvant chemoradiotherapy. Finally, baseline investigation of myeloid cells within tumor tissue did not differ between survival groups, indicating immune surveillance of peripheral blood during adjuvant therapy may be a critical missing link to educate our understanding of the immune effects of standard of care therapy for glioblastoma.

## 1 Introduction

Even with the standard of care therapy of maximal safe resection followed by adjuvant chemoradiotherapy (CRT), patients with glioblastoma have an exceedingly poor prognosis with inevitable local recurrence and progression-free survival at six months of 54% (1, 2). Systemic immune dysfunction, including lymphopenia, induced by the tumor and therapy, may be important to the failure of existing treatment paradigms and is currently the focus of investigation (3). Here we explore the effect of CRT, the standard cytotoxic therapy for glioblastoma, in dynamically altering the systemic immune environment through increase in regulatory myeloid populations thereby inhibiting the anti-neoplastic tumor response and limiting effectiveness. An understanding of this effect of treatment that occurs in some patients may guide development of strategies to improve outcome.

Peripheral lymphocytes, essential in anti-neoplastic immune response, are extremely radiosensitive and the substantial decrease observed in a large proportion of patients with glioblastoma undergoing radiotherapy has been attributed to the relatively high volume of circulating blood exposed to radiation in patients receiving radiotherapy to the brain (4). Consequently, radiotherapy-induced lymphopenia is a predictor of poor outcomes across multiple solid tumor types and is associated with decreased tumor control (4–7). The clinical impact of treatment-induced lymphopenia has been prospectively studied and is a consequence of standard therapy (6). However, persistent lymphopenia is observed in only a proportion of patients and persists for a lengthy period of time without the expected recovery (8, 9). We hypothesize that the variability and persistence may be explained by an off-target effects of a course of daily fractionated radiation that lead to a physiological state mirroring chronic inflammation. A principal component of this state is the dysregulated immunosuppressive response mediated mainly by increased myeloid-derived suppressor cells (MDSC) resulting in prolonged lymphopenia, that is intriguingly a homeostatic protective mechanism against prolonged unwanted inflammatory response in other CNS injury such as stroke or trauma occurring in the absence of any direct lymphotoxic events (10).

Malignant tumors, and glioblastoma in particular, are known to hijack normal homeostatic responses to reduce inflammation in chronic injury which also suppresses antineoplastic immunity*. Ex vivo* and *in vitro* studies in peripheral blood samples from patients with glioblastoma prior to therapy report the presence of dysfunctional systemic immunity with increased T lymphocyte helper 2 (Th2) (IL-10, IL-4, IL-6) (11, 12) and decreased Th1 (IFNγ, TNFα, IL-1β) cytokines (13). These cytokine profiles are not unique to glioblastoma and are found in many chronic disease processes including diabetes, heart disease, and renal failure (14–16). Further, anti-inflammatory cytokine signature in resected glioblastoma tissue was recently demonstrated to be a negative prognostic indicator suggesting that understanding patterns of systemic cytokine expression in glioblastoma may be valuable (17). Since immune cell function and phenotype are closely linked to the composition of soluble factors, it is not surprising that there is altered cellular homeostasis in glioblastoma. MDSC, a heterogeneous group of immature myeloid cells, are believed to support the immune evasiveness reported in glioblastoma (18). MDSC frequency at the time of surgical resection positively correlates with grade in patients with glioma and the expansion of this cell population portends a poorer prognosis (19, 20). Several studies have implicated MDSC in tumor promotion through immunosuppressive mechanisms (21, 22).

MDSC can be recruited to tumors following exposure to radiation, likely contributing to the immunosuppressive local tumor environment (18). Radiation has also been demonstrated to initiate a cascade of anti-tumor immunity, which activates and aids the immune response to the tumor through both innate and adaptive immunity (23). Despite the known impact of RT, very little is known about how peripheral MDSC frequency and activation status changes over time in patients with glioblastoma during adjuvant CRT and whether differential kinetics can be correlated to clinical benefit. Here we report on the longitudinal changes in peripheral immune sub-populations in patients with glioblastoma during six weeks of standard CRT and importantly, evaluate associations of these effects with tumor outcome.

## 2 Materials and methods

### 2.1 Human subjects

Our research protocol was approved by our institutional review board and ethics committee at the Johns Hopkins University School of Medicine. All participants gave written informed consent for participation in the study. We recruited 16 patients over the age of 18 years old with a pathologic diagnosis of glioblastoma based on the CNS5 WHO Classification of Tumors of the Central Nervous System from the Sidney Kimmel Comprehensive Cancer Center (SKCCC) at Johns Hopkins Hospital (JHH) who were planned to receive adjuvant CRT (24). Patients with recurrent glioblastoma were excluded. Blood kits were prepared by SKCCC Central Kit Service and delivered to the clinical laboratories. Researchers completed and passed all JHH Biosafety Training Modules prior to the handling of human blood samples. Donor blood was transported in a BioTransport Carrier (Nalgene). A total of 210-mL of blood, seven-30mL blood draws, was collected: once after surgery but before CT simulation and five times weekly during CRT. Research blood was collected at the time of standard lab draws when possible. Donor-matched formalin-fixed paraffin-embedded tumor tissue was collected from each participant.

### 2.2 Isolation of PBMC and flow cytometry analysis

Donor blood was handled within a biosafety cabinet that meets OSHA and JHH standards. PBMC were isolated from heparinized whole blood by Ficoll density gradient separation. PBMC were then counted after Trypan Blue staining using a hemocytometer. PBMC phenotype and activation status were analyzed by multiparametric FC. Cells were incubated with pre-titrated fluorochrome-conjugated α-human antibodies including CD14, CD4, CD8, CD16, CD33, CD163, CD80, CD86, HLA-DR, CSF1R, PD-1, PD-L1, LAG3, TIM-3, CTLA-4, TIGIT, VSIG4, and BTLA4. Non-specific binding was blocked with BD Fc Block and True Stain Block. Dead cells were excluded with a viability stain. PBMC were permeabilized and stained with antibodies to intracellular targets including IL-10, IFNγ, M-CSF, IL-34, TGFβ1, and FoxP3. All FC was performed through the Flow Cytometry and Human Immunology Technology Center in the Bloomberg~Kimmel Institute for Cancer Immunotherapy at JHH. Raw data was collected on a BD FACS Celesta. 20,000 (monocytes within PBMC), 10,000 (monocytes within whole blood), 125,000 (unstimulated lymphocytes), 30,000 (stimulated lymphocyte) events were collected for each test. Singlets were identified within a broad myeloid cell or lymphocyte gate, defined by forward and side scatter properties, and immunophenotyped (MDSC= CD33^+^HLA-DR^-^). FCS files were analyzed using FlowJo v10 (10.6.2.) software.

### 2.3 Bulk RNAseq

Monocytes were isolated from PBMC using the EasySep Cell Separation magnetic bead kit. CD14^+^ monocytes were freshly isolated through negative selection and cryopreserved in 90% FBS and 10% DMSO. Total RNA was extracted from thawed monocytes using RNEasy micro-kits (Qiagen) and quantified. Peripheral monocyte transcriptomic profiles were generated by bulk RNAseq. Counts data were analyzed for differential expression using a negative binomial model implemented with DESeq2 v1.34.0 (25), which was also used to normalize expression values for visualizations via variance stabilizing transformation. For all response metadata, time point was included in the design matrix unless samples were subset to a specific time point. The resulting differential expression results were analyzed with gene set enrichment analysis using fgsea v1.20.0 with a selection of gene sets relevant to glioblastoma, taken from the Molecular Signatures Database (26).

### 2.4 NanoString digital spatial profiling

NanoString Digital Spatial Profiling (DSP) technology was used to analyze protein expression in formalin-fixed paraffin-embedded tumor tissue. Four immunofluorescence markers were used to identify regions of interest (CD163=myeloid cells, CD3= T-cells, GFAP= Tumor/Astrocytes, DAPI= nuclei) with the help of a neuropathologist for multiplex targeting. Only CD163^+^ annotated spots were included to control for presence of immune cell populations. The NanoString GeoMX DSP analysis kit was used for scaling and normalization of expression data followed by t-test for statistical comparisons. Spots were then split by responder status and analyzed by multiple t-test with multiple testing correction using the Benjamini-Hochberg method.

### 2.5 Statistical analysis

Patients with missing baseline values were excluded from all analyses of temporal immune changes. In addition, those with missing subsequent immune response during CRT were also excluded. Descriptive analysis of log fold change at both time points 3 and 5 during CRT was presented in tables by patients’ survival status at 2 years after diagnosis. The Mann–Whitney U test was used to compare log fold change between survivors and non-survivors. FDR-adjusted p-values were provided to account for the multiplicity of hypothesis testing. An FDR-adjusted p-value of 0.05 indicates that 5% of significant tests will result in false positives. The temporal immune changes were visualized as scatterplots showing log fold change from pre-CRT baseline over the course of CRT by survival status. LOWESS (locally weighted scatterplot smoothing) was used, and the associated 95% confidence intervals were shown in gray.

The correlation between RNA seq and CD14^+^ flow data at each time point was visualized by scatterplots, and fitted using linear regression model. Pairwise Pearson correlation coefficient was used to quantify the correlation between RNA seq and CD14^+^ flow data. Exact p-values from Kendall’s test were provided to test for correlation between paired samples.

Percent frequency of PD-L1^+^ MDSC and CD8^+^IFNγ^+^ lymphocytes was presented as box plots and median at all time points. Percent frequency of lymphocyte populations over the course of CRT was visualized as Spaghetti plots, smoothed by LOWESS.

## 3 Results

### 3.1 Characteristics of the participants and summary of clinical management

During October 2018 to June 2019, a total of 16 patients were enrolled at a single institution. Of these, 14 patients completed the study while two stopped early: one declined active management of their cancer and the other withdrew from further participation mid-study (**Supplementary Figure 1**). Participant baseline characteristics are shown in **Table 1**. The majority of patients (68.8%) underwent surgical resection for glioblastoma followed by adjuvant CRT with temozolomide and 60 Gy of radiotherapy in 30 fractions, considered the gold-standard management strategy for glioblastoma. 56.3% (n=9) of participants received a gross total resection and 43.7% (n=7) received a subtotal resection. Four participants were enrolled in concurrent interventional clinical trials. Three of these explored alterations in RT dose, target, or fraction size. Additionally, one clinical trial tested an experimental therapeutic as an alternative to temozolomide. Follow-up data for outcomes were available through September 4, 2021. The median follow-up period was 16.2 months (range, 3.5 to 35.6 months). No patients were lost to follow-up.

**Table 1:** Patient Demographics.

	Total Cohort (n=16)
Demographics	
Median Age at diagnosis	63 yrs (range- 46-73 yrs)
Sex Assigned at Birth	
Female	3 (18.8%)
Geographic region - no. (%)	
North America	16/16 (100%)
Europe	0/16 (0%)
Asia	0/16 (0%)
Rest of World	0/16 (0%)
Hispanic or Latinx ethnic group	0/16 (0%)
Pathological Features	
MGMT Promoter Methylated	4/16 (25%)
IDH1/2 mutation	1/16 (6.3%)
Extent of Resection	
Gross Total Resection	9/16 (56.3%)
Subtotal Resection	7/16 (43.7%)
Biopsy Only	0/16 (0.0%)
Treatment Details (ClinicalTrials.gov Identifier, if applicable)	
Received for Standard Adjuvant CRT	11*/16 (68.8%)
Altered Radiation Therapy Dose Spectroscopy-guided Dose Escalation (NCT03137888)	1/16 (6.3%)
Altered Radiation Therapy Target Standard + SV Zone (NCT02177578)	1/16 (6.3%)
Altered Radiation Therapy Fraction Size Low Dose Fractionated RT (NCT01466686)	1/16 (6.3%)
Altered Systemic Therapy TMZ Alternative, BAL101553 (NCT03250299)	1/16 (6.3%)

*One patient did not start planned adjuvant CRT.

### 3.2 MDSC frequency in patients with glioblastoma

**Figure 1:**
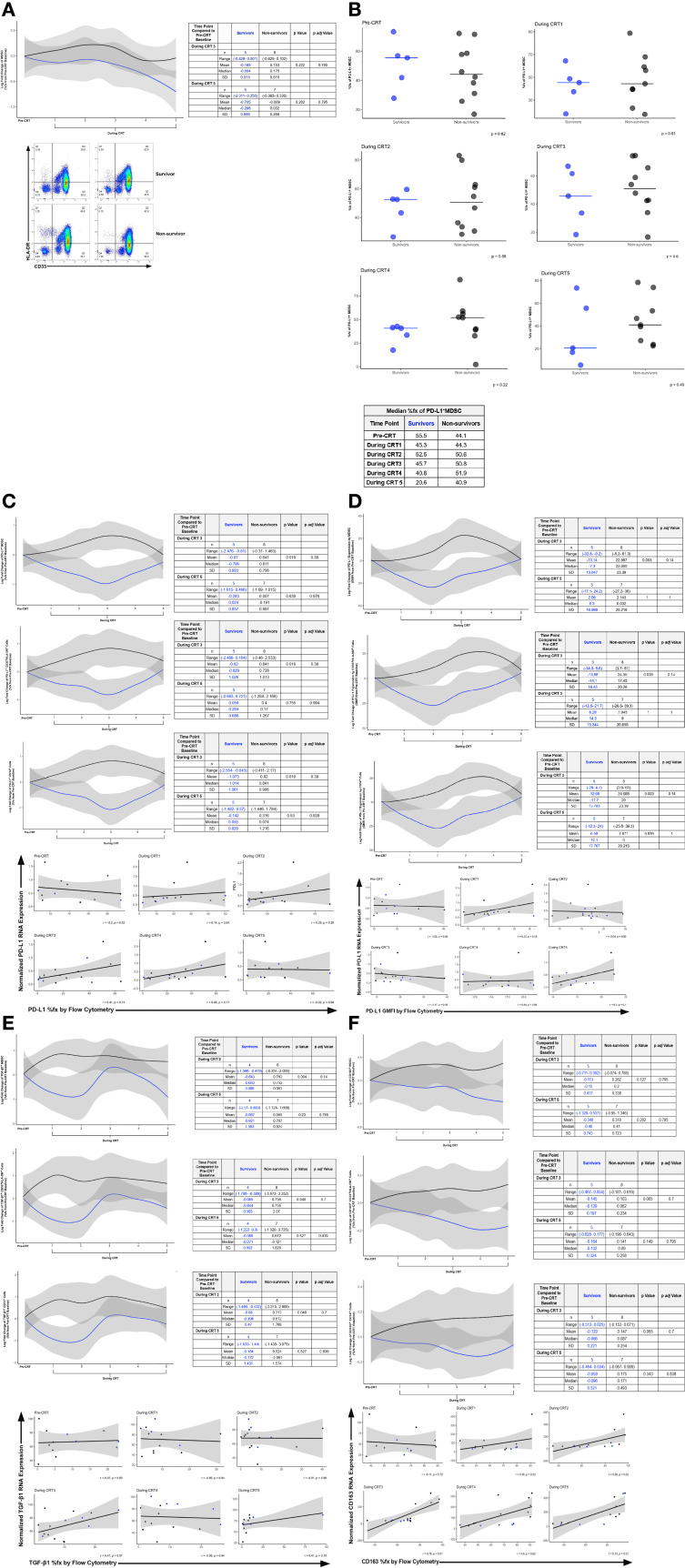
**(A)** Log Fold Change of Percent Frequency of MDSC before and during CRT. The line graph shows modeled log fold change data from survivors (blue line) and non-survivors (black line) over the course of CRT (top). Example Flow Cytometry Scatter Plots of MDSC Percent Frequency (bottom). MDSC were defined using the gating strategy described within the **Supplementary Materials**. Dot plots are shown from survivors (top row) and non-survivors (bottom row) at pre-CRT (left) and CRT TP 5 (right). CD33 and HLA-DR expression were used to identify MDSC within Q3. Scatterplot arrows indicate increasing expression of CD33 or HLA-DR. **(B)** Percent Frequency (%fx) of PD-L1^+^ MDSC in Survivors (blue) and Non-survivors (black) during CRT. **(C)** Log Fold Change of the Percent Frequency of PD-L1^+^ MDSC, PD-L1^+^ CD33^+^HLA-DR^+^ Cells, and PD-L1^+^ CD14^+^ Cells before and during CRT in Survivors and Non-survivors. The line graphs show modeled log fold change data from survivors (blue line) and non-survivors (black line) over the course of CRT (top). Correlation was performed between normalized PD-L1 RNA expression (y-axis) and PD-L1%fx by flow cytometry in CD14^+^ cells (x-axis) (bottom). **(D)** Log Fold Change of the Geometric Mean Fluorescence Intensity of PD-L1 Expression by MDSC, CD33^+^ HLA-DR^+^ Cells, and CD14^+^ before and during CRT in Survivors and Non-survivors. The line graphs show modeled log fold change data from survivors (blue line) and non-survivors (black line) over the course of CRT (top). Correlation between PD-L1 RNA expression and PD-L1 %fx by flow cytometry in CD14^+^ cells (bottom). **(E)** Log Fold Change of the Percent Frequency of TGF-β1^+^ MDSC, TGF-β1^+^ CD33^+^ HLA-DR^+^ Cells, and TGF-β1^+^ CD14^+^ Cells before and during CRT in Survivors and Non-survivors. The line graphs show modeled log fold change data from survivors (blue line) and non-survivors (black line) over the course of CRT (top). Correlation between TGF-β1 RNA expression and TGF-β1%fx by flow cytometry in CD14^+^ cells (bottom). **(F)** Log Fold Change of the Percent Frequency of CD163^+^ MDSC, CD163^+^CD33^+^HLA-DR^+^ Cells, and CD163^+^ CD14^+^ Cells before and during CRT in Survivors and Non-survivors. The line graphs show modeled log fold change data from survivors (blue line) and non-survivors (black line) over the course of CRT. The line graphs show modeled log fold change data from survivors (blue line) and non-survivors (black line) over the course of CRT (top). Correlation between CD163 RNA Expression and CD163% fx by flow cytometry in CD14^+^ cells (bottom).

Elevated peripheral MDSC numbers have been causally linked to exacerbated post-CRT lymphopenia in glioblastoma. In our study, we first correlated dynamic changes in peripheral MDSC over the course of CRT with survival outcome. The log fold change of MDSC frequency from “pre-CRT” baseline, throughout adjuvant CRT (“during CRT”) was compared in participants who were alive at two years after diagnosis (“survivors”) compared to those who were deceased at two years after diagnosis (“non-survivors”). MDSC were defined through gating strategy including CD33^+^HLA-DR^-^ cell surface expression by flow cytometry (**Supplementary Figures 2A–D**). The mean log fold change in the percent frequency of MDSC at CRT 3 compared to baseline was -0.169 (± 0.515) for two-year survivors and 0.153 (± 0.702) for non-survivors (n=8) (**Figure 1A**, top). At the end of concurrent treatment, the mean log fold change in the percent frequency of MDSC at CRT 5 compared to baseline was -0.705 (± 0.985) for survivors and -0.009 (± 0.330) for non-survivors (n=7) (**Figure 1A**, top). **Figure 1A**, bottom demonstrates the flow cytometry quadrant gate used to identify MDSC based on CD33 and HLA-DR expression. MDSC percent frequency at pre-CRT baseline (55.5%, 44.1%) and during CRT (range, 20.8%-52.8% to 44.3%-51.9%) were similar in survivors and non-survivors (**Figure 1B**). Alternative gating strategies for peripheral myeloid populations, including CD14^+^ cells and CD33^+^HLA-DR^+^ cells were also employed to determine degrees of overlap with the MDSC population (**Supplementary Figures 3E–J**). The log fold change of programmed death-ligand 1 (PD-L1) expressing peripheral blood myeloid populations (MDSC, CD33^+^HLA-DR^+^ cells, CD14^+^ cells) increased from baseline in non-survivors but decreased in survivors (**Figure 1C**). CRT 3 survivors (n=5) had a mean PD-L1^+^ MDSC %fx of -0.970 (± 0.993) and non-survivors (n=8) had a mean of 0.641 (± 0.706). At the end of CRT, this difference abated with a mean PD-L1^+^ MDSC %fx of -0.283 (± 0.857) in survivors (n=5) and 0.007 (± 0.997) in non-survivors (n=7). Geometric mean fluorescent intensity (GMFI) of PD-L1 followed similar expression levels to cell surface percent frequency, but with a lag in increase in amount of PD-L1 expression per cell after starting CRT by non-survivors in myeloid cells based on all three gating strategies employed (**Figure 1D**). We also assessed differences in MDSC TGF-β1 expression over the course of CRT (**Figure 1E**). CRT 2 survivors (n=4) had a mean TGF-β1^+^ MDSC %fx of -0.843 (± 0.496) and the non-survivors (n=8) had a mean of 0.763 (± 0.903). At CRT 5, this difference remained disparate with a mean TGF-β1^+^ MDSC %fx of -0.867 (± 1.393) in survivors (n=4) and 0.565 (± 0.924) in non-survivors (n=7). The %fx of CD163 expression by group also differed (**Figure 1F**). CRT 3 survivors (n=5) had a mean CD163^+^ MDSC %fx of -0.113 (± 0.417) and the non-survivors (n=8) had a mean of 0.262 (± 0.338). For CRT 5, this difference remained disparate with a mean CD163^+^ MDSC %fx of -0.348 (± 0.743) in survivors (n=5) and 0.318 (± 0.723) in non-survivors (n=7). The expression of myeloid-relevant cell surface markers and soluble factors of interest by flow cytometry are shown in **Supplementary Figures 4A–H**.

### 3.3 T cell response and relationship to MDSC frequency

**Figure 2:**
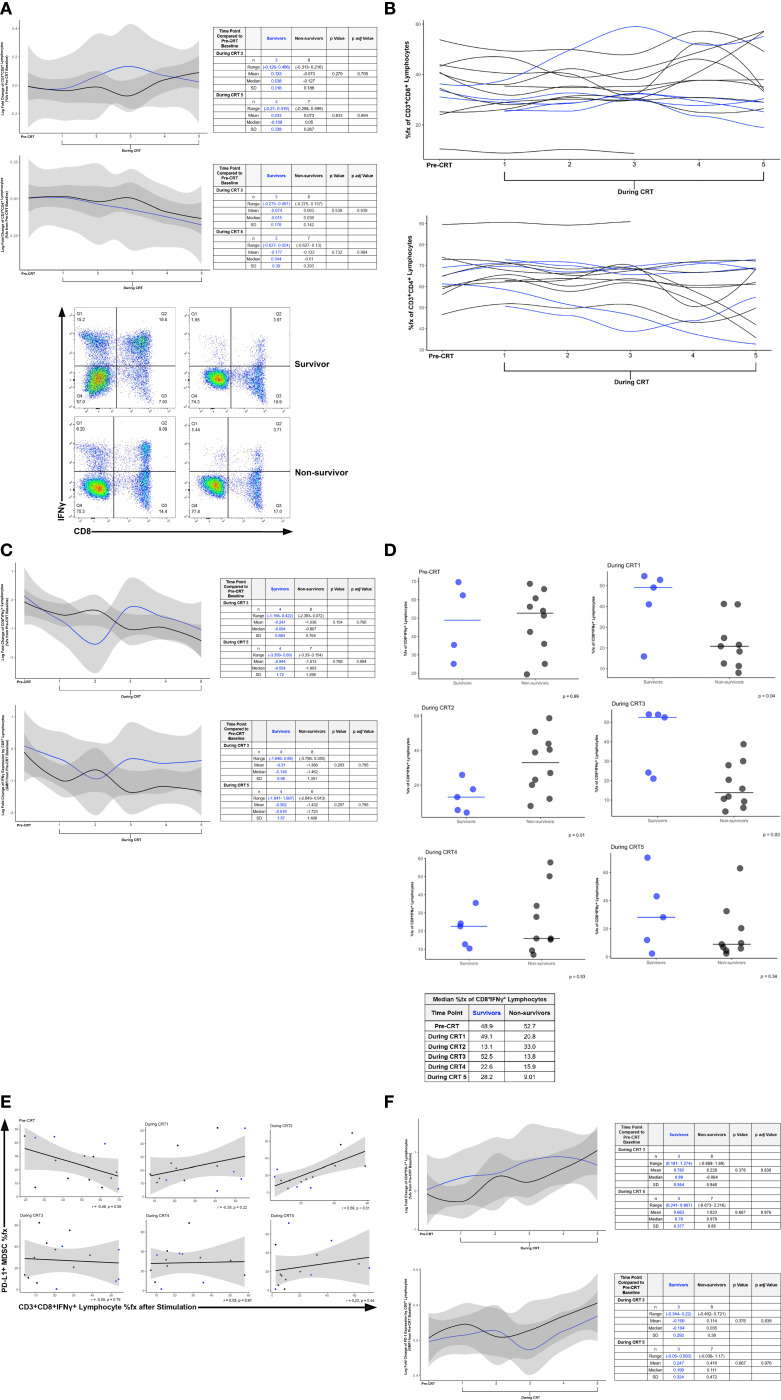
**(A)** Log Fold Change of CD8 (top line graph) and CD4 (bottom line graph) Percent Frequency (%fx) by CD3^+^ Lymphocytes before and during CRT in Survivors and Non-survivors. The line graphs show modeled log fold change data from survivors (blue line) and non-survivors (black line) over the course of CRT (top). Example Flow Cytometry Scatter Plots of CD8^+^IFNγ^+^ Percent Frequency (bottom). IFNγ expressing CD8^+^ lymphocytes were defined using the gating strategy described within the **Supplementary Materials**. Dot plots are shown from survivors (top row) and non-survivors (bottom row) at pre-CRT (left) and CRT TP 5. Q2 identifies CD8^+^IFNγ^+^ lymphocytes. **(B)** Percent Frequency of Lymphocyte Populations over the Course of CRT: CD3^+^CD8^+^ Lymphocyte %fx (top), CD3^+^CD4^+^ Lymphocyte %fx (bottom). **(C)** Log Fold Change of IFNγ Expression by Stimulated Lymphocytes before and during CRT in Survivors and Non-survivors. The line graphs show modeled log fold change data from survivors (blue line) and non-survivors (black line) over the course of CRT. The log fold change of the %fx (top) and expression per CD3^+^CD8^+^ lymphocyte (bottom) of IFNγ are displayed. **(D)** Percent Frequency of CD8^+^IFNγ^+^ Lymphocytes in Survivors and Non-survivors during CRT. **(E)** Correlation between PDL1^+^ MDSC %fx by Flow Cytometry and CD3^+^CD8^+^IFNγ^+^ Lymphocyte %fx after Stimulation. **(F)** Log Fold Change of PD-1 Expression by Stimulated CD8^+^ Lymphocytes before and during CRT in Survivors and Non-survivors. Freshly isolated peripheral blood mononuclear cells were stained and %fx (top) and GMFI (bottom) of PD-1 expression by CD8+ lymphocytes was identified. The line graphs show modeled log fold change data from survivors (blue line) and non-survivors (black line) over the course of CRT.

The anti-tumor T-cell-centric immune adjuvanticity of RT is well-established (27). Thus, RT efficacy is intrinsically dependent on T cells (28), but at the same time, RT can “self-limit” by promoting lymphopenia. In our next set of cellular analyses, we focused on whether CRT-associated changes in the MDSC landscape were also reflected in both qualitative and quantitative perturbations to the T cell compartment. Cytotoxic CD3^+^CD8^+^ (**Figure 2A**, line graph, top) lymphocyte percent frequency in freshly stained peripheral blood samples identified by flow cytometry, more so increased in the peripheral blood than CD3^+^CD4^+^ lymphocytes with a slight decrease in CD3^+^CD4^+^ at the end of CRT (**Figure 2A**, line graph, bottom). Using a gating strategy described in **Supplementary Figures 5A, B**, the percent frequency of the CD8^+^ IFNγ-expressing population was identified. Example scatter plots of two participants before CRT and during CRT 5 is depicted in **Figure 2A**, bottom. This is demonstrated by spaghetti plot for each lymphocyte subtype in **Figure 2B**. Intracellular flow cytometry was used to determine the log fold change of IFNγ expression by stimulated CD8^+^ lymphocytes before and during CRT in survivors and non-survivors (**Figure 2C**, top). The mean log fold change from pre-CRT baseline to CRT 3 in the %fx of IFNγ^+^CD8^+^ lymphocytes was -0.241 (± 0.684) for survivors (n=4) and -1.036 (± 0.764) for non-survivors (n=8). At CRT 5, the mean log fold change in %fx of IFNγ^+^CD8^+^ lymphocytes in survivors (n=4) was -0.944 (± 1.720) and -1.513 (± 1.298) in non-survivors (n=7) at CRT 5. **Figure 2D** depicts the %fx of CD8^+^IFNγ^+^ lymphocytes based on survival group. A positive correlation was identified between PD-L1^+^ MDSC %fx by flow cytometry and CD8^+^IFNγ^+^ lymphocyte %fx after stimulation (**Figure 2E**). Log fold change of PD-1 %fx by stimulated CD8^+^ lymphocytes before and during CRT (**Figure 2F**, top) differed between survivors and non-survivors. The mean % fx of survivors at CRT 3 was a mean of 0.782 (± 0.554) in survivors (n=3) and 0.228 (± 0.948) in non-survivors (n=8). The gap further widened later in concurrent treatment during CRT 5 with a mean log fold change in %fx of 0.663 (± 0.377) in survivors (n=3) and 1.023 (± 0.850) in non-survivors (n=7). Similar increases in PD-1 GMFI were seen (**Figure 2F**, bottom). The mean log fold changes from pre-CRT baseline in survivors (n=3) and non-survivors (n=8) at CRT 3 was -0.106 (± 0.324) and 0.114 (± 0.380), respectively. The mean log fold change of PD-1 GMFI for CD8^+^ lymphocytes was 0.247 (± 0.324) in survivors (n=3) and 0.416 (± 0.472) in non-survivors (n=7). The expression of T lymphocyte-relevant cell surface markers and soluble factors of interest by flow cytometry are shown in **Supplementary Figures 7A–G** for CD8^+^ lymphocytes and **Supplementary Figures 8A–G** for CD4^+^ lymphocytes. The correlation between TGF-β1^+^ MDSC %fx and CD3^+^CD8^+^IFNγ^+^ lymphocyte frequency after stimulation and CD163^+^ MDSC %fx and CD3^+^CD8^+^IFNγ^+^ lymphocyte frequency after stimulation are depicted in **Supplementary Figures 9A, B**, respectively.

### 3.4 Effect of chemoradiotherapy on the peripheral myeloid transcriptome

**Figure 3:**
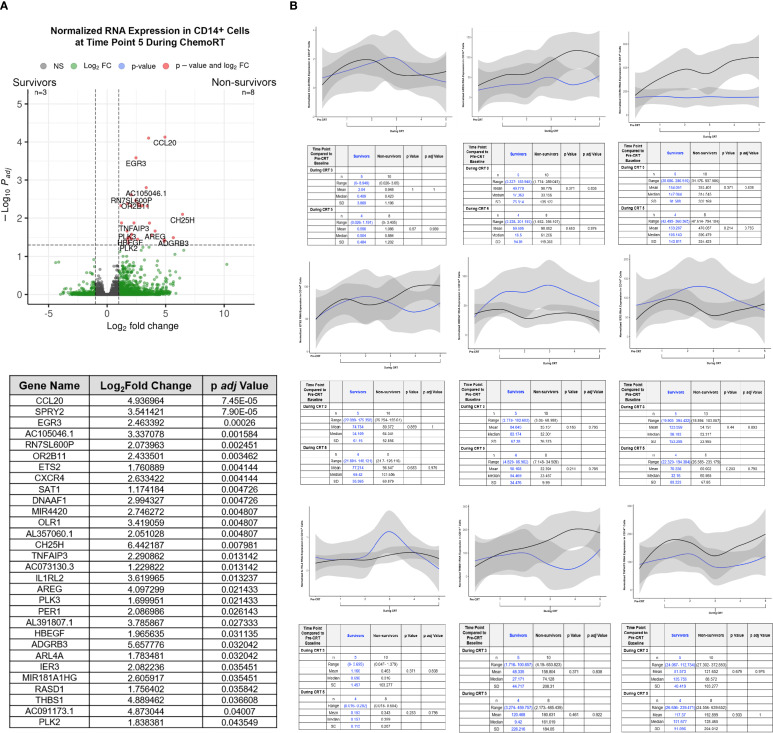
**(A)** RNA Expression by CD14^+^ Monocytes by Bulk RNAseq at CRT 5. **(B)** Normalized RNA Expression during CRT of CCL20, AREG, CXCR4, Ets2, HBEGF, IER3, IL1RL2, THBS1, and TNFAIP3 by Bulk RNAseq in CD14^+^ Cells. The line graph show modeled data from survivors (blue line) and non-survivors (black line) over the course of CRT.

In the aftermath of CRT, patients with lymphopenia have been shown to harbor significantly elevated MDSC-specific gene signatures relative to baseline and concomitantly lower effector T and NK cell gene signatures (29). Here, we wanted to evaluate whether the peripheral MDSC phenotype (e.g. PD-L1^low^) observed in our survivor cohort of patients with glioblastoma (**Figure 1**) had a corresponding transcriptomic repertoire that was less regulatory and more differentiated/activated. For this, we performed bulk RNA sequencing on peripheral monocytes collected prior to the start of CRT and five times during CRT, the same time points as the flow cytometric analysis. Total RNA was extracted from CD14^+^ monocytes (including monocytic MDSC) that had been isolated through negative (untouched) selection from participant peripheral blood mononuclear cells (PBMC). The volcano plot in **Figure 3A** identifies transcripts that were significantly elevated in non-survivors (n=8) compared to survivors (n=3) at CRT 5 (**Figure 3A**, top plot). Of the thirty genes found to be significantly upregulated, several are linked to specialized myeloid cell function (**Figure 3A**, bottom table). These include immune-active genes (**Figure 3B**) such as chemokine (C-C motif) ligand 20 (CCL20), amphiregulin (AREG), C-X-C chemokine receptor type 4 (CXCR4), ets2 transcription factor (ETS2), heparin-binding epidermal growth factor (EGF)-like growth factor (HBEGF), immediate early response 3 (IER3), interleukin 1 receptor like 2 (IL1RL2), and thrombospondin 1 (THBS1). Each gene is reported in the form of normalized RNA expression from housekeeping genes. Interestingly, survivor-non-survivor differences in peripheral monocyte gene expression at other study time points were not apparent. Volcano plots for these time points are shown in **Supplementary Figures 10A–E**.

### 3.5 Spatial profiling of the baseline glioblastoma tumor microenvironment

**Figure 4:**
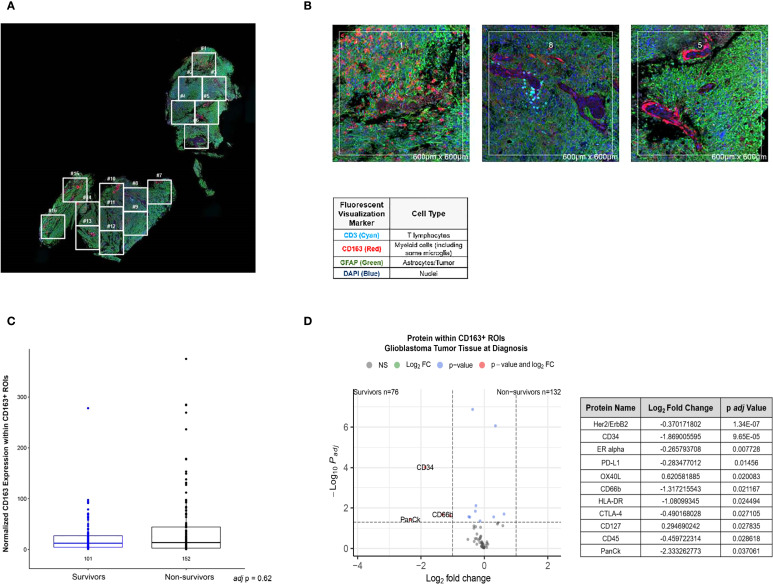
**(A)** Digital Spatial Profiling (DSP) Example of Glioblastoma Tumor Tissue. Full slide example of one of ten glioblastoma cases analyzed by DSP. **(B)** Example regions of interest (ROIs) Spatial Profiling. Magnified views (600 x 600 μm) of example regions of interest (ROIs).Visualization markers were used to identify ROIs by immunofluorescence. **(C)** Normalized Expression of CD163 in CD163^+^ ROIs in Glioblastoma Tumor Tissue. **(D)** Enriched Proteins in Glioblastoma Tissue by DSP at the Time of Resection.

Spatial analyses of the glioblastoma tumor microenvironment have yielded lucid insight into the functional linkage between the spatial cellular and transcriptomic landscape and disease prognosis (30). Here, we have undertaken a similar effort to characterize the spatial expression of select immune and stromal protein markers in baseline tumor sections from our respective survivor and non-survivor cohorts. Selection of regions of interest (ROIs) in participant FFPE tissue was guided by immunofluorescent staining of CD163 for myeloid cells (some macrophages, MDSC, microglia), CD3 (T lymphocytes), and GFAP (tumor and astrocytes) in conjunction with an expert neuropathologist. **Figure 4A** depicts the FFPE slide identifying selected ROIs with magnified examples of ROIs found in **Figure 4B** from one selected non-survivor. **Supplementary Figures 11A, B** demonstrate an example of digital spatial profiling (DSP) in tissue of a survivor. Additional spatial profiling imaging can be found CD163 expression was compared between CD163^+^ ROIs by immunofluorescence in survivors (n=2) and non-survivors (n=8), where no difference was found in tissue (**Figure 4C**). CD163 expression by ROI was identified (**Supplementary Figure 12A** for non-survivor example and **Supplementary Figure 12B** for survivor example). Spatial profiling of ROIs for protein expression with CD163^+^ ROIs were compared between survivors (n ROIs= 76) and non-survivors (n ROIs= 132) and represented as a volcano plot (**Figure 4D**).

## 4 Discussion

A comprehensive characterization of the temporal immune changes occurring over a conventionally fractionated course of CRT has not been reported. To that end, our aggregate readout or “radiation immunodynamics” described in this study is expected to offer translatable insight into the utility of second-line immunotherapy for glioblastoma and other cancer types eligible for frontline CRT. Here, we sought to understand how standard CRT affects kinetics of peripheral MDSC emergence, and subsequently, link these changes to any clinical benefit. We determined that the peripheral MDSC proportions displayed an increasing trend throughout the course of CRT relative to baseline in patients who did not survive at two years after diagnosis whereas patients who survived at two years displayed a downward trend relative to baseline. This is a critical finding that has not previously been reported.

In our interrogation of the immune landscape in patients with glioblastoma undergoing active management with CRT, we relay findings of the serial analysis of fresh peripheral blood samples with matched spatial cellular analyses (Digital spatial profiling: DSP) from tumor tissue obtained at initial resection. Like in the periphery, we identified the myeloid burden to be similar in resected tumor tissue between responders and non-responders (**Figure 4C**). Our DSP results focused on CD163^+^ rich regions within tumor to approximate immune signatures of immunosuppressive myeloid cells in glioblastoma. Among our findings, OX40L was highly enriched in tumor samples obtained from non-survivors (**Figure 4D**). OX40L is a ligand of OX40, member of the tumor necrosis factor superfamily that promotes T cell activation, as well as longevity (31). It is possible this upregulation of OX40L, which is known to be expressed by myeloid cells among others, in non-survivors seen in our data is reflective of early signs of additional immunologic challenge within the tumor microenvironment in patients with poor outcomes. Potentially, even at this early time point prior to adjuvant therapy, either a compensatory upregulation of T cell activation markers has been triggered and/or a tumor-permissive “type 2” inhibition of immune response has already been selected in patients with glioblastoma (31, 32). Interestingly, preclinical evidence suggests that OX40L is further upregulated by radiotherapy (33), suggesting that the immune suppression in glioblastoma is indeed multilayered. Once easily obtained and reliable biomarkers are successfully identified in the peripheral immune compartment, this could allow for discovery of tissue-based markers that predict treatment-related immune side effects that exist even prior to initiation of CRT.

The role of the immune system and the complex immunity seen in patients with glioblastoma may be one limitation to our current approach to developing new therapies for this patient subpopulation. The glioblastoma tumor microenvironment is potently suppressive. Glioblastoma is known as an immunologically cold tumor with an abundance of suppressive myeloid cells (microglia/macrophages/MDSC) that foster an environment inimical to T lymphocyte priming and activation (34, 35). The addition of immune-modifying stimuli, in this case, standard therapy of CRT that most patients receive, makes immunity a dynamic variable, especially when considering the longitudinal nature of anti-neoplastic therapy packages. Recently Akkari et al., characterized the interaction of myeloid cells and fractionated radiation using a mouse model of glioblastoma and patient biospecimens, and concluded that radiotherapy increased PD-L1^+^ MDSC (36), a finding that was also observed in our participant cohort. Immune-targeting therapies have yet to be demonstrated as effective for the treatment of glioblastoma, with responses to immunotherapy only occurring in a select number of patients (37). Recent strategies have focused on circumventing immune suppression within the tumor microenvironment by priming T cells *ex vivo*, and some have been promising in that regard, as in the case of a Phase 3 clinical trial of a dendritic cell vaccine, DCVax-L (38). This also highlights the severity of immune dysfunction prevalent in the glioblastoma TME and warrants novel lines of investigation to identify effective reversal strategies.

Our dataset is unique and to our knowledge there has never been a dedicated prospective clinical trial reporting findings of freshly stained myeloid cells from patients with glioblastoma receiving CRT at this many time points. Lymphopenia has been shown to be inversely linked to MDSC frequency using cryopreserved PBMC from a cohort of patients with glioblastoma receiving CRT (29). There is a large body of evidence showing myeloid-related immune markers, especially lineage makers needed to identify MDSC by flow cytometry, are markedly altered by cryopreservation (39, 40). Although these data support the importance of the peripheral immune system in glioblastoma, studies utilizing fresh samples are critical for elucidating the immune response occurring in patients. Additionally, only a few prospective and longitudinal studies have been undertaken to date that could serve as templates for future studies attempting to interrogate immune changes as they relate to pre- and post-radiotherapy. As mentioned, because changes in myeloid cell subsets can reflect systemic inflammation outside of cancer, each participant’s baseline may be an important factor to consider when evaluating the effects of cancer therapy. Here, we chose to consider the individual contribution to myeloid cells and represent data in terms of log fold change from pre-CRT baseline as opposed to raw quantitative values that may not be comparable within the context of the larger cohort. Additional studies are needed to understand how myeloid changes can best be represented to accurately reflect clinical relevance of cell populations.

We identified only a modest decrease in the frequency of CD8^+^IFNγ^+^ lymphocytes at the end of CRT (“during CRT 5” time point) in non-survivors compared to survivors (**Figure 2D**, bottom right). As lymphopenia persists in some patients following CRT, our data could suggest that the expected decrease in lymphocyte count is more pronounced in the weeks to months following treatment, opposed to during the last week of CRT reported here. Additional studies quantifying lymphocyte levels that span from the end of RT to distant, post-CRT time points could begin to answer this question. Quantifying lymphocytes through experimental means using FCA, as opposed to using clinical complete blood cell count testing utilized by many prior studies to identify lymphopenia, may have also played a role in modest differences in lymphocyte counts in survivors and non-survivors that we observed. It is also possible that CRT has differential effects on specific lymphocyte populations. Although our spaghetti plots of lymphocyte frequency did not reveal noticeable decreases in CD3^+^CD8^+^ lymphocytes, nor CD3^+^CD4^+^ lymphocytes (**Figure 2B**), many lymphocyte subsets that we tested did display alterations with CRT (**Supplementary Figures 7**, **8**). These data include a notable, yet unexpected, rise in the frequency of CD8^+^TIGIT^+^ lymphocytes (**Supplementary Figure 7F**, top) and CD4^+^TIGIT^+^ lymphocytes (**Supplementary Figure 8G**, top) at the CRT5 time point, only in survivors. Further research with fresh biosamples is needed to understand how CRT affects lymphocyte subsets, especially those bearing targetable checkpoint molecules.

The results of our study also suggest that there may be more to learn about the function of the immune system when challenged by commonly utilized clinical strategies, like CRT. Peripheral blood samples from patients were first fractionated into CD14^+^ monocytes including the MDSC compartment before being analyzed for transcriptomic changes. Many myeloid-associated genes were found to have increased mid-CRT. Many of these genes relate to chemotaxis, including CXCR4 and CCL20. Altered immune states that support myeloid recruitment has been shown to be an important mechanism of myeloid cell influx into the brain in chronic disease (14–16), neuroinflammatory disease (41), and glioblastoma, specifically (22). Perhaps, other neurotherapeutics could be borrowed and tested as adjuvants in brain tumor patients receiving CRT. Importantly, differences in gene expression were most striking at the latest CRT time point we assessed (CRT5), with minimal changes early in CRT and few to none at mid-CRT time points (**Supplementary Figure 10**). These data may suggest that temporal immunologic thresholds may exist within a six-week course of CRT that could be addressed through future personalized radiotherapy approaches.

We employed spatial proteomics to compare myeloid cells in the TME in resected GBM tumor tissue between survivors and non-survivors. We considered this biosample to be reflective of the pre-CRT local immune environment which we know is densely populated with myeloid cells from the peripheral blood like MDSC. After confirming baseline CD163 expression in ROIs were similar among survivors and non-survivors (**Figure 4C**), comparison of baseline tissue identified enriched genes (**Figure 4D**). While survivors had more expression of the hematopoietic stem cell marker, CD34, within CD163^+^ ROIs, PD-L1 expression was significantly elevated in resected tissue from non-survivors. These findings could indicate that presence of invading of peripheral myeloid cells matters less to prognosis than the specific phenotype they assume in tumor. Spatialomics data from invading myeloid cells from resected tumor could be extrapolated and used to inform individualized immunotherapy strategies to neutralize MDSC niches that may remain after surgery.

In evaluating the impact of this treatment, it is important to be cognizant of the possible immune-enhancing effect of radiotherapy initiated via on-target effects. Radiation can elicit increased crosstalk between dying tumor cells and responder immune cells via damage-associated molecular patterns (DAMPs) (42), the basis for the exclusively immune-mediated abscopal effect on distant tumors (43). Emerging evidence suggests the STING pathway may be critical to this and is relevant to glioblastoma specifically (44). The challenge, therefore, lies in modulating CRT to unleash immune effectors such as cytotoxic CD8^+^ T cells against radio-resistant tumor cells and concomitantly limit the activity of immunoregulatory cells including MDSC.

There were limitations to our pilot study. Larger and adequately-powered studies would need to be conducted in order to conclusively correlate MDSC patterns with glioblastoma outcomes. An additional limitation of our study relates to the prospective nature of the collection of samples which occurred in 2018 when the 4^th^ Edition World Health Organization of Classification of Tumors of Central Nervous Systems criteria was used for the diagnosis of brain tumors (45). More recently, the WHO has released molecular-based profiling with integrated diagnoses. Within our cohort of 16 patients, only one participant had a pathologically-confirmed IDH1/2 mutation (**Table 1**). Not surprisingly, this same participant had previously received radiotherapy for a lower grade primary astrocytic tumor. The participant was co-enrolled in an interventional clinical trial where radiotherapy was delivered with an altered fractionation pattern which did not include daily radiotherapy for six weeks and was, thus, excluded from the many of the analyses presented above. In essence, although the former classification was used to diagnose glioblastoma for the patients within this cohort, our data are reflective of CNS WHO grade 4 glioblastoma diagnoses under the current revised classification system (24).

Further limitations in the tissue microenvironment relate to the plasticity of resident and myeloid subsets. Our data demonstrates that baseline expression of CD163, a myeloid cell marker that can be used to estimate myeloid burden in tissue, is expressed at a similar level in tumor tissue at the time of resection in survivors and non-survivors. Although some tissue biomarkers may be diagnostic and prognostic for gliomas such as MGMT promoter methylation and isocitrate dehydrogenase (IDH) mutations (46, 47), it is possible that any conclusions drawn about the immune landscape from analyses of tissue at the time of surgical resection may be too static to reflect the complex immune processes that are occurring throughout the disease course in response to anti-cancer therapy. Alternatively, CD163 as a marker of immunosuppressive myeloid cells in tumor may not be sensitive enough to detect the diverse myeloid populations in the brain and could be underestimating or reflecting only a small subset of myeloid cells (48, 49). More studies are needed to be able to accurately trace peripheral-origin/monocyte-derived myeloid cells that eventually infiltrate the tumor microenvironment.

It is known that CRT and even radiotherapy alone can cause lymphopenia and poor tumor control. We hypothesized that the variability across patients and lack of long-term recovery is related to induction of MDSC within a wider immunologic state that may mimic other chronic disease states. We found that increases in MDSC did occur during CRT in some patients and this was associated with poor outcome. Further, long term survivors had less change from baseline MDSC values at the end of CRT. Changes in other cell populations (cytotoxic CD8^+^ T lymphocytes, Tregs), cytokines, and cell phenotype were consistent with MDSC accumulation as a driver. Ultimately, our prospective study supports our hypothesis that CRT supports immunosuppressive myeloid cell phenotypes, with MDSC being increased in non-survivors compared to survivors in our cohort. This finding was further corroborated by corresponding impairment in the in CD8^+^ lymphocyte function as seen in dampened IFNγ and elevation of PD-1 expression at the end of CRT. Therefore, understanding the temporal dynamic effects of fractionated radiotherapy, or radiation immunodynamics, is crucial to decoding immunosuppression seen in glioblastoma. The results of this study will inform future research and clinical trials aimed at modifying and improving treatment to limit suppression of the antineoplastic immune response, perhaps based on real time changes measurable in individual patients. As the type of inflammation identified here can be applied to chronic disease, the application of these findings may have potential to translate beyond the effect of standard treatment for glioblastoma. We plan further studies to examine these and other cell populations in a larger cohort of patients, and ultimately design trials that will test modification of therapy based on dynamic changes in individual patients.

## Data Availability

The raw data supporting the conclusions of this article will be made available by the authors, without undue reservation.
