# Supplementary material for: Radiation immunodynamics in patients with glioblastoma receiving chemoradiation
Source: Front Immunol. 2024 Sep 13;15:1438044. doi: 10.3389/fimmu.2024.1438044 (PMC11427284; doi:10.3389/fimmu.2024.1438044)
Supplement: Supplementary file 1 [file DataSheet1.docx]

Supplementary Material

# Supplementary Figures


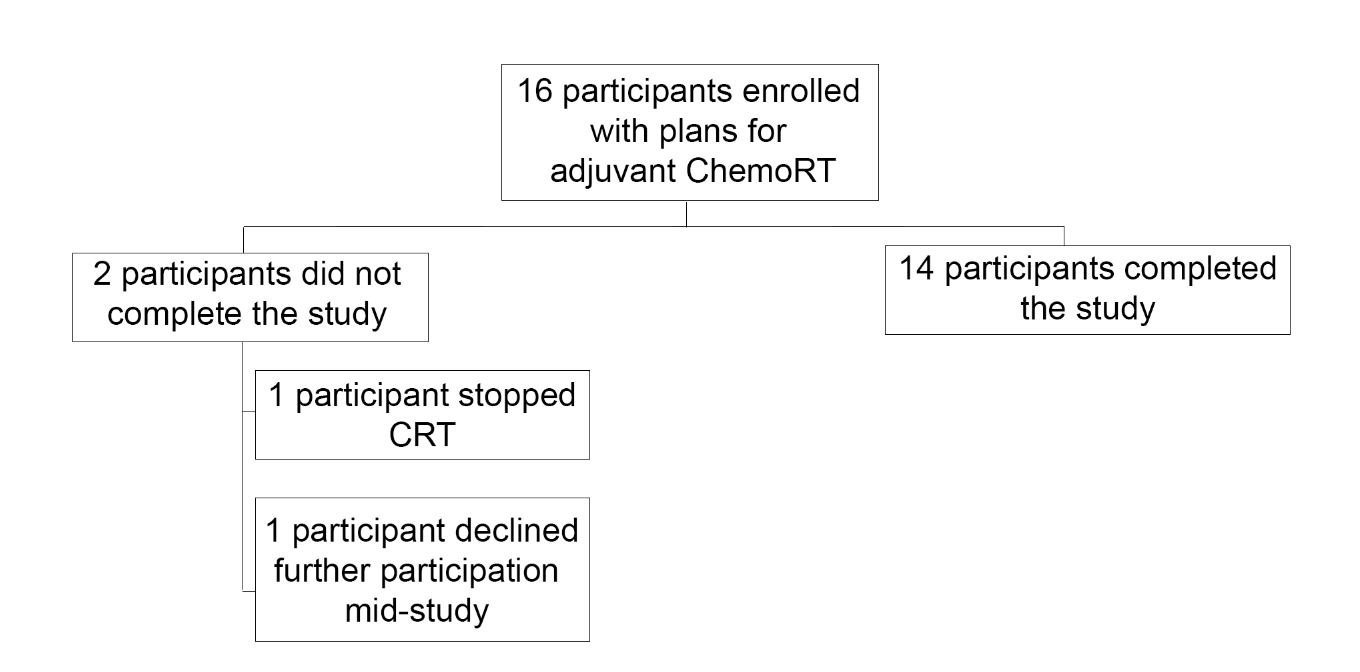


**Supplementary Figure 1.** Flow Diagram of the Enrollment of Study Participants. CRT= chemoradiotherapy.


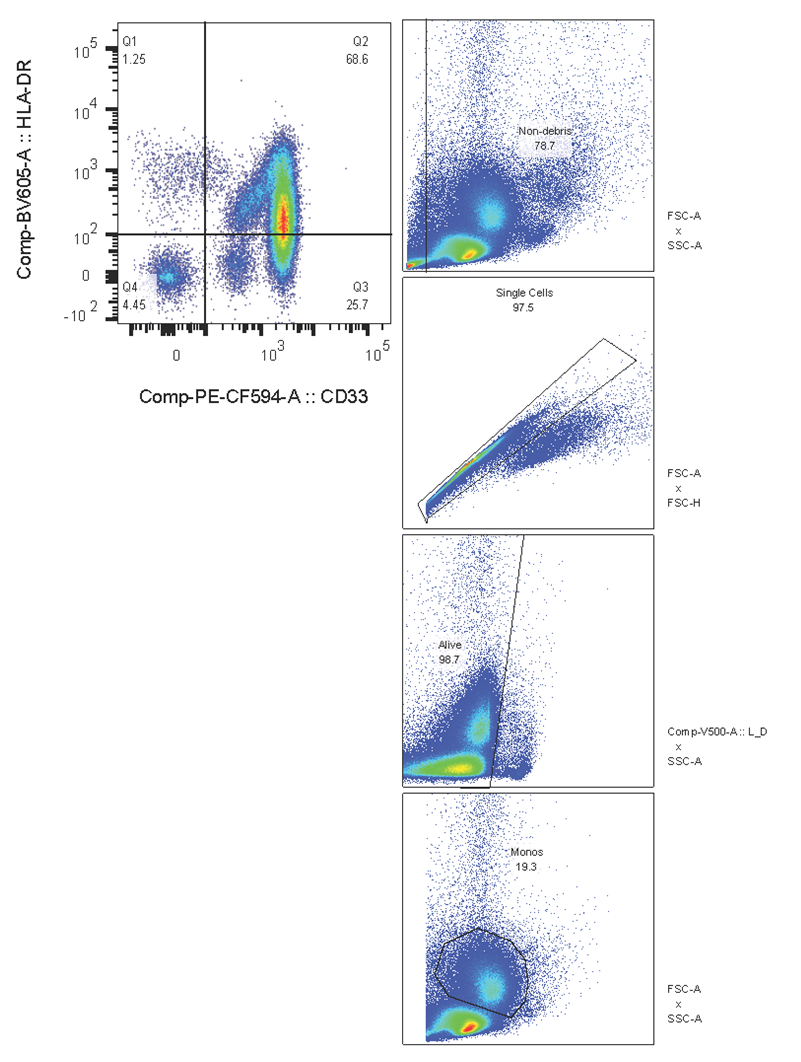


**Supplementary Figure 2a.** Gating Strategy Example of MDSC from Participant A.


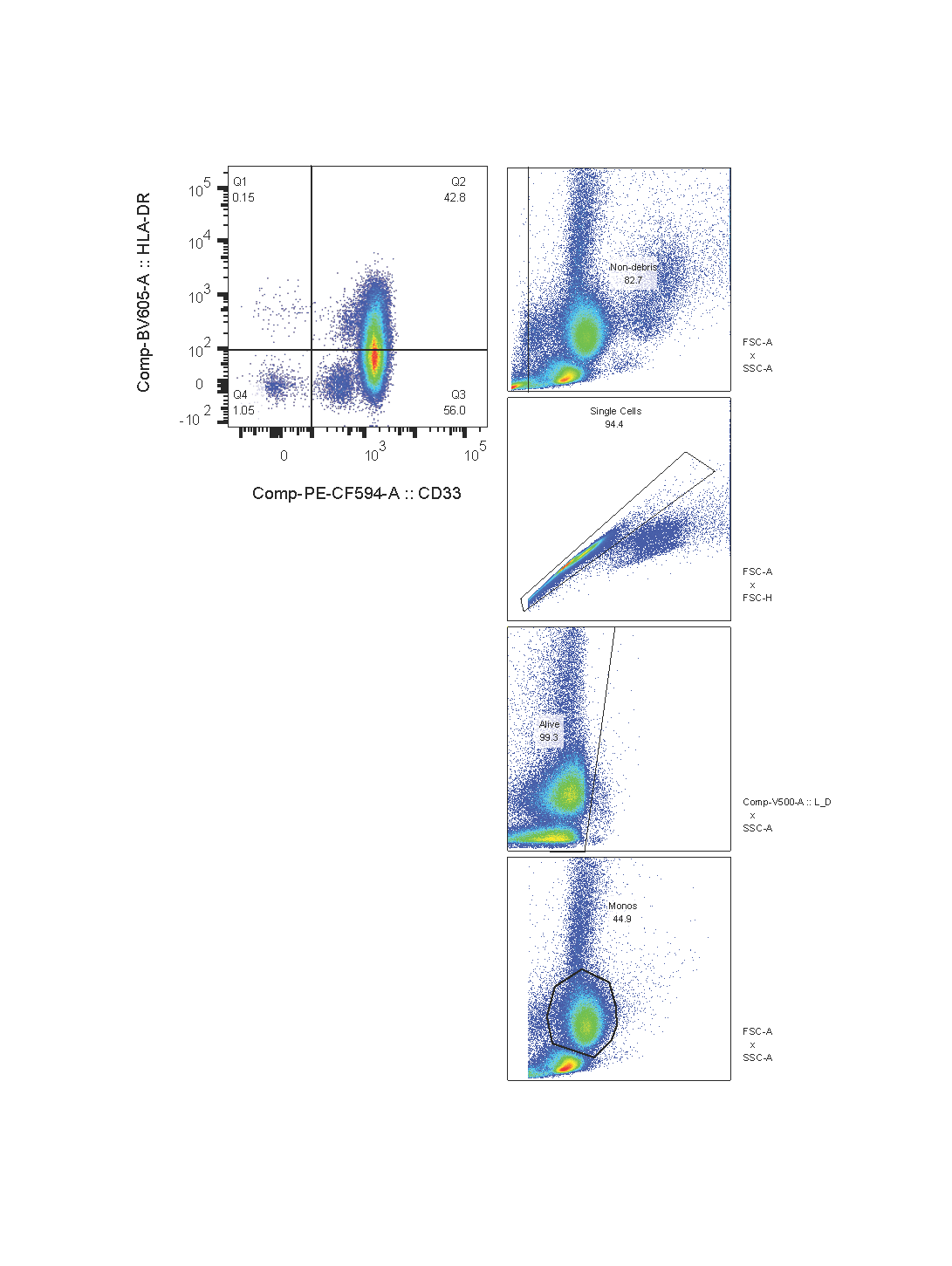


**Supplementary Figure 2b.** Gating Strategy Example of MDSC from Participant B.


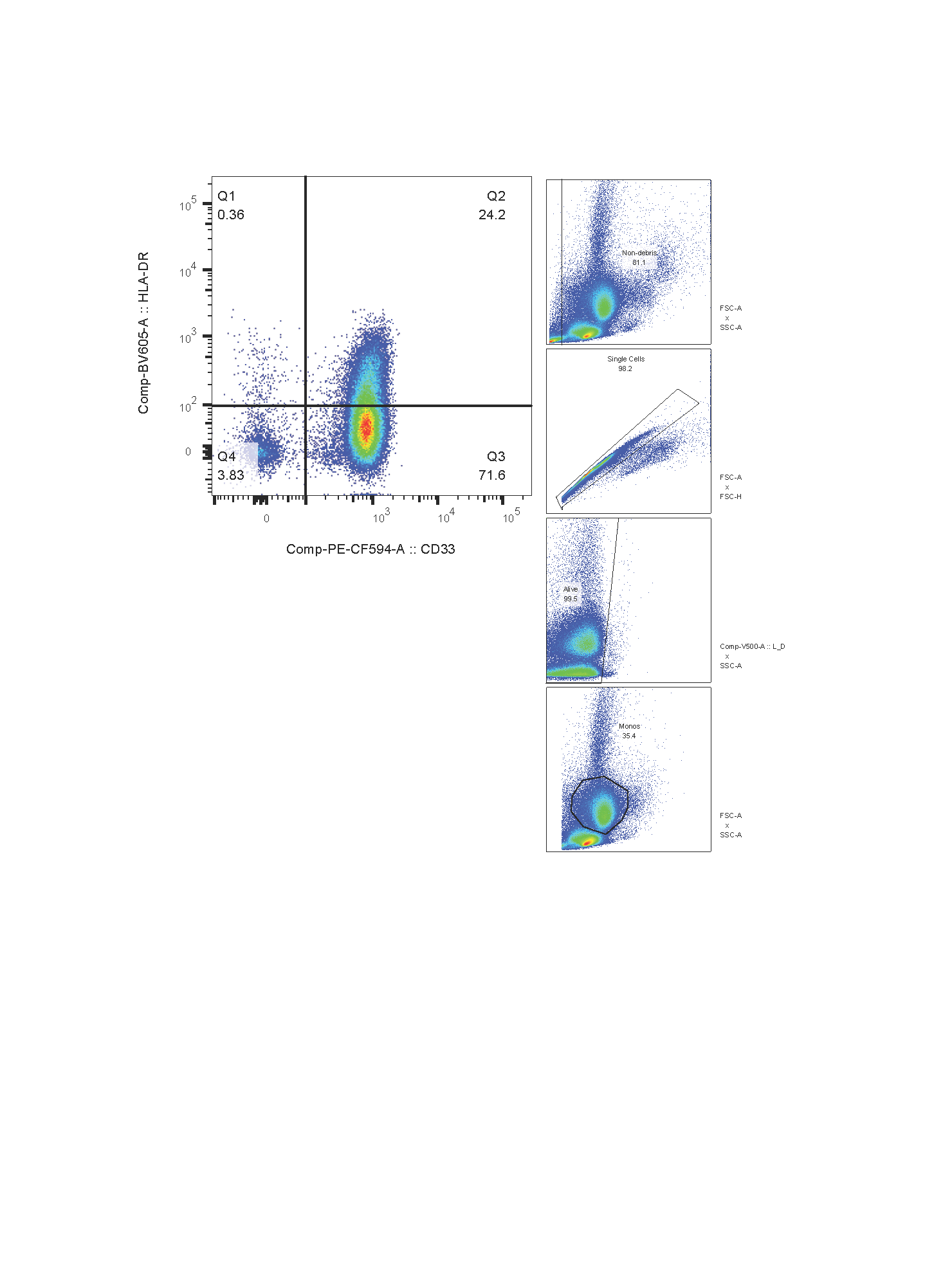


**Supplementary Figure 2c.** Gating Strategy Example of MDSC from Participant C.


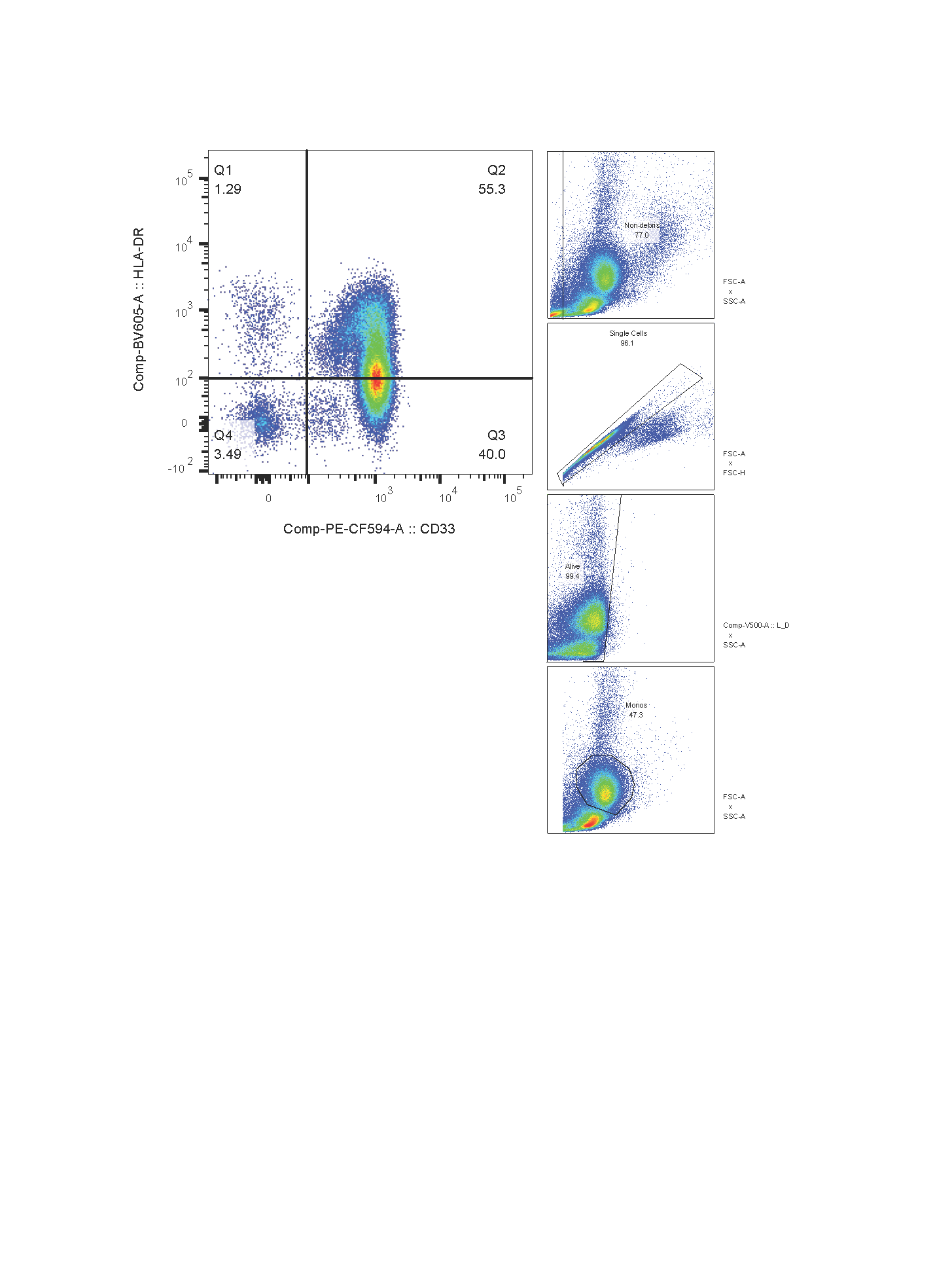


**Supplementary Figure 2d.** Gating Strategy Example of MDSC from Participant D.


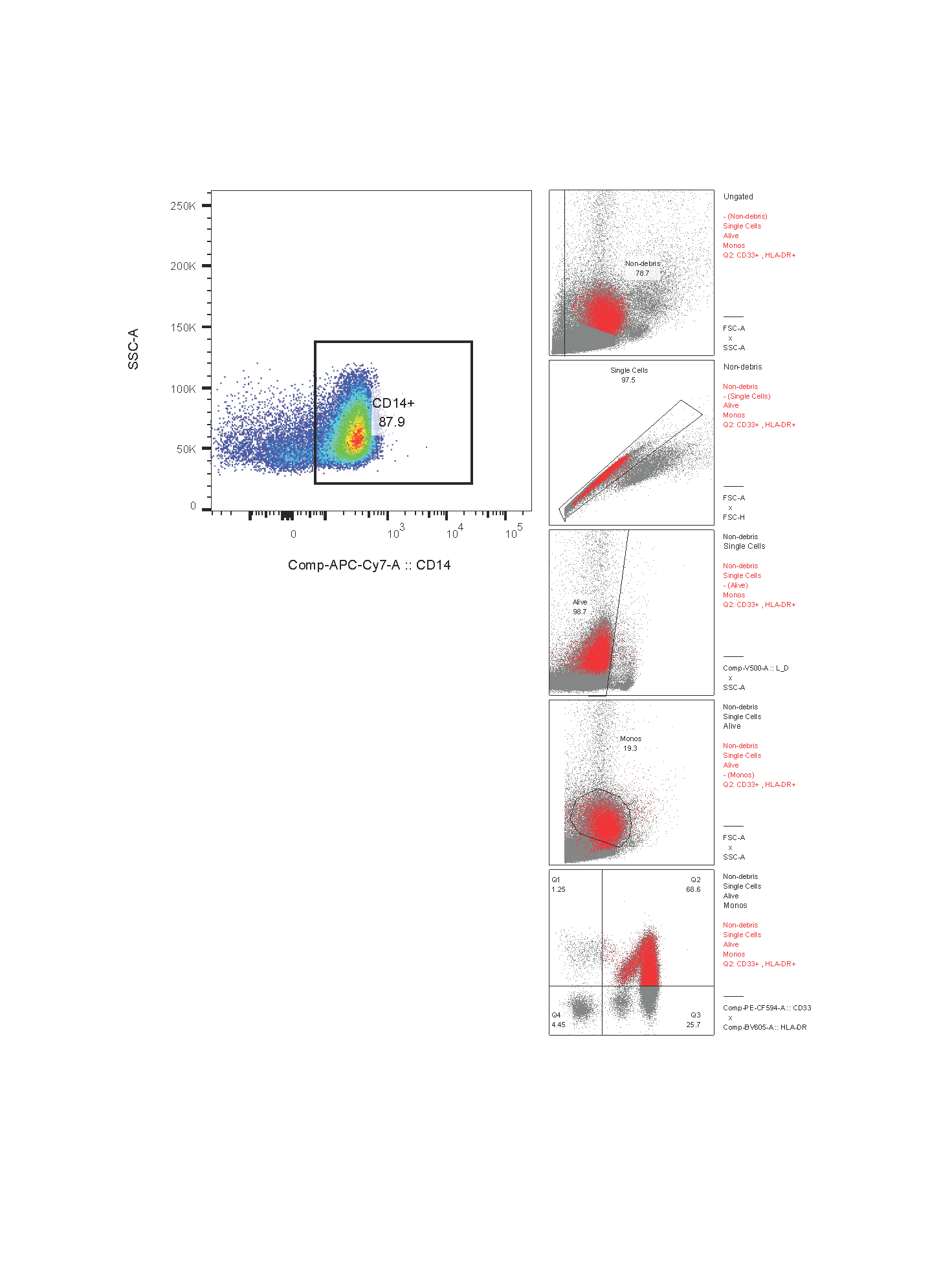


**Supplementary Figure 3a.** Example of Backgating of CD33^+^HLA-DR^+^ Myeloid Cells that Co-Express CD14 in the Peripheral Blood of Participant A.


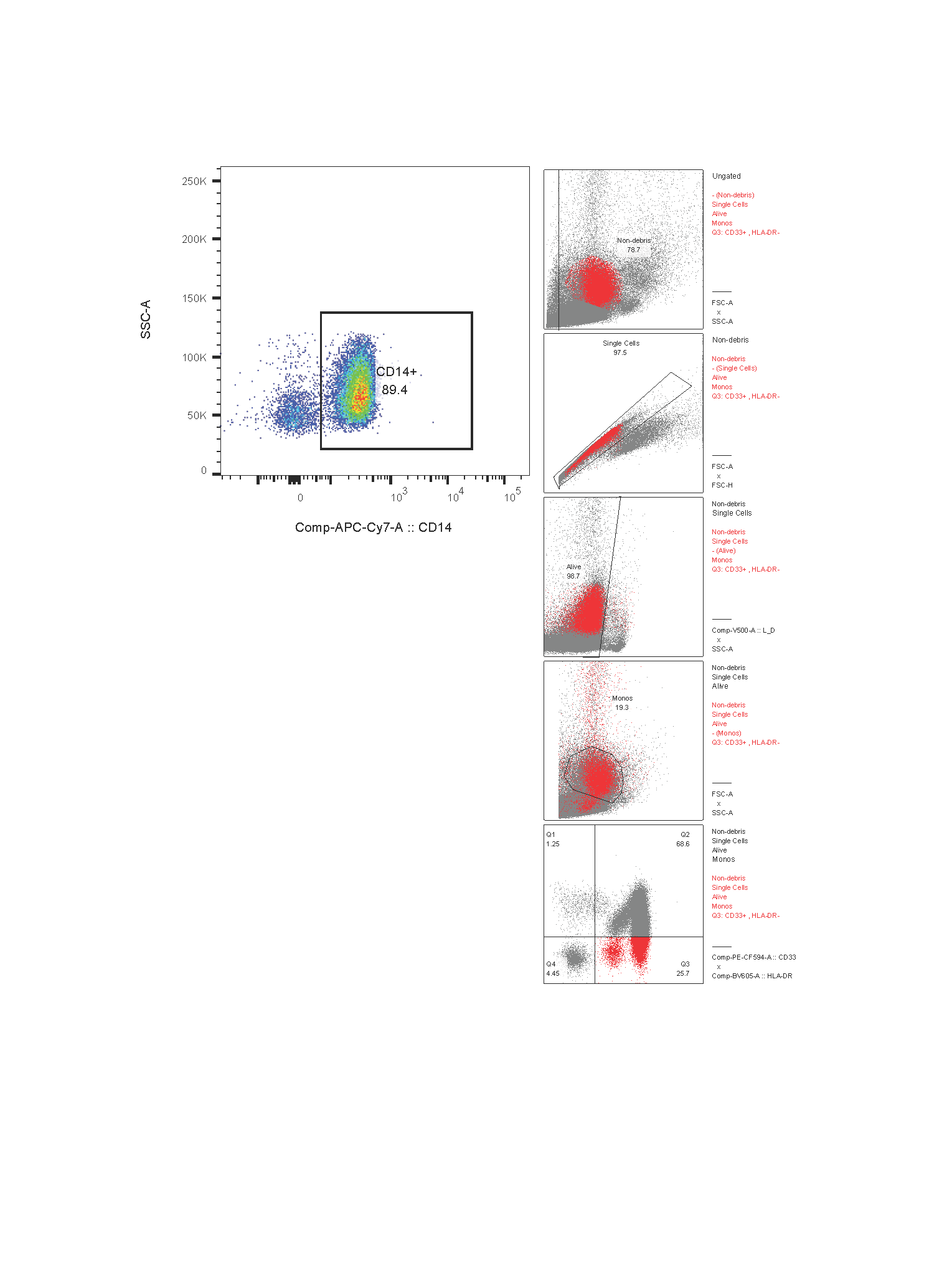


**Supplementary Figure 3b.** Example of Backgating of MDSC that Co-express CD14 in Peripheral Blood of Participant A.


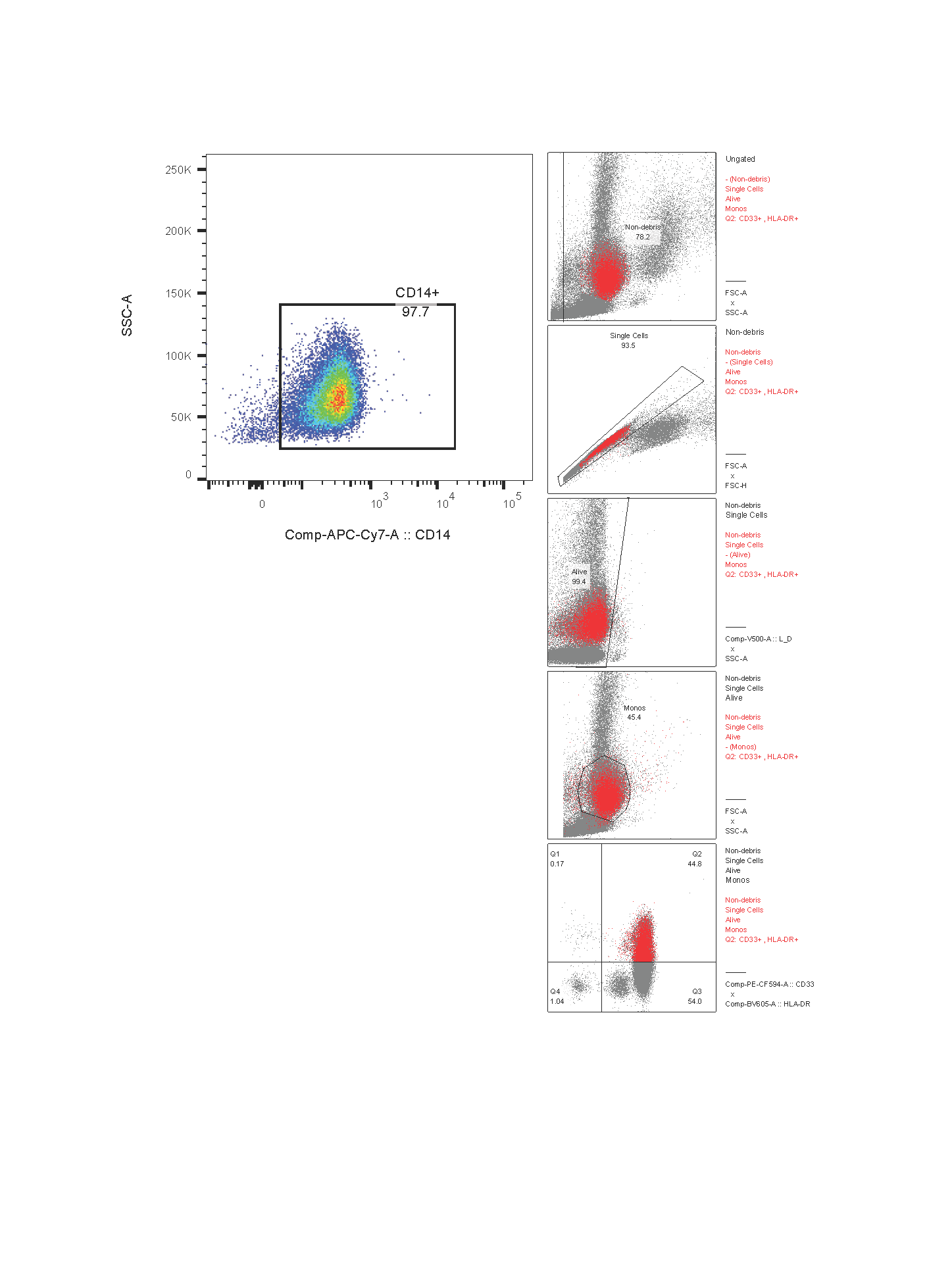


**Supplementary Figure 3c.** Example of Backgating of CD33^+^HLA-DR^+^ Myeloid Cells that Co-express CD14 in the Peripheral Blood of Participant B.


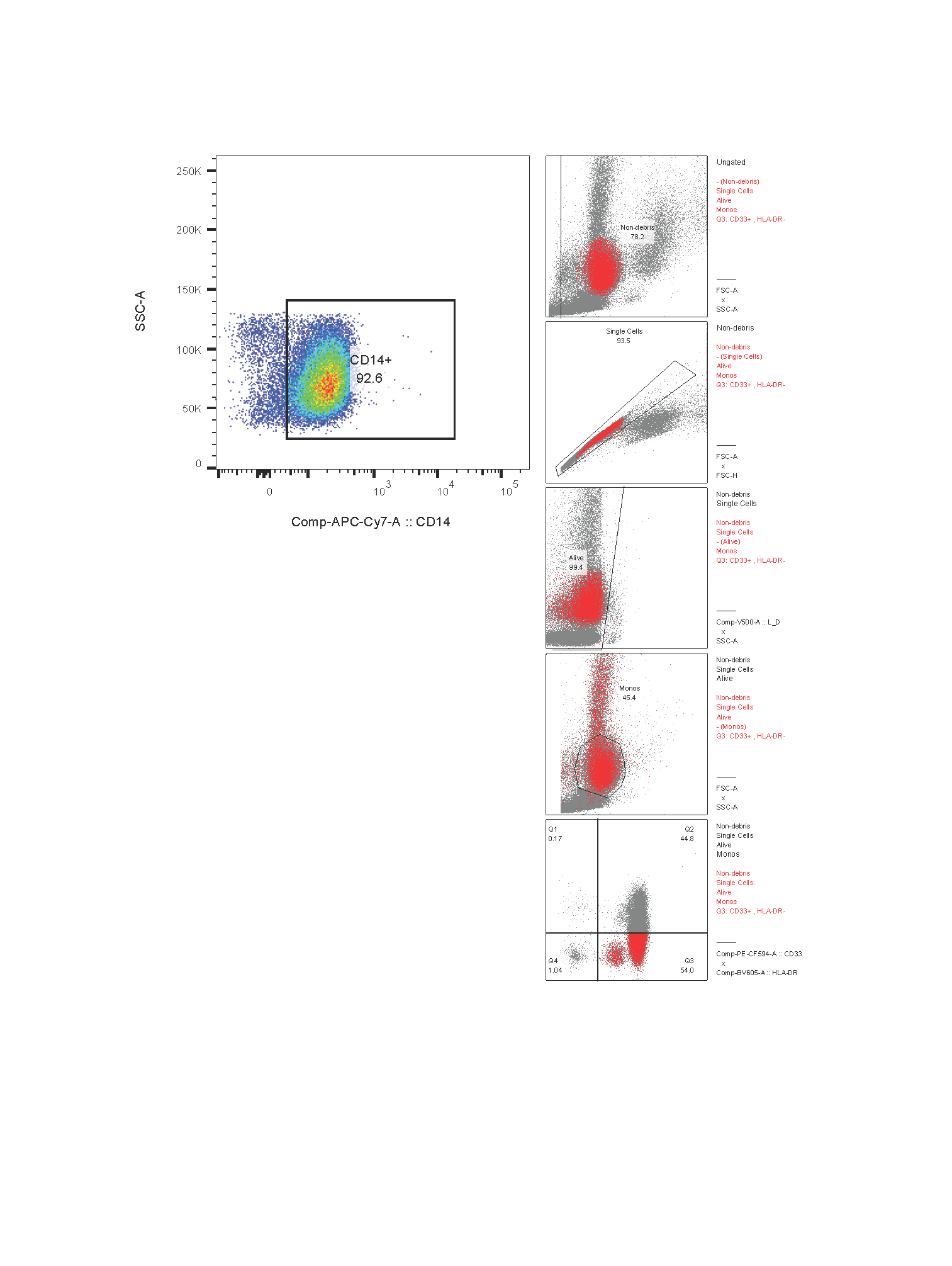


**Supplementary Figure 3d.** Example of Backgating of MDSC that Co-express CD14 in the Peripheral Blood of Participant B.


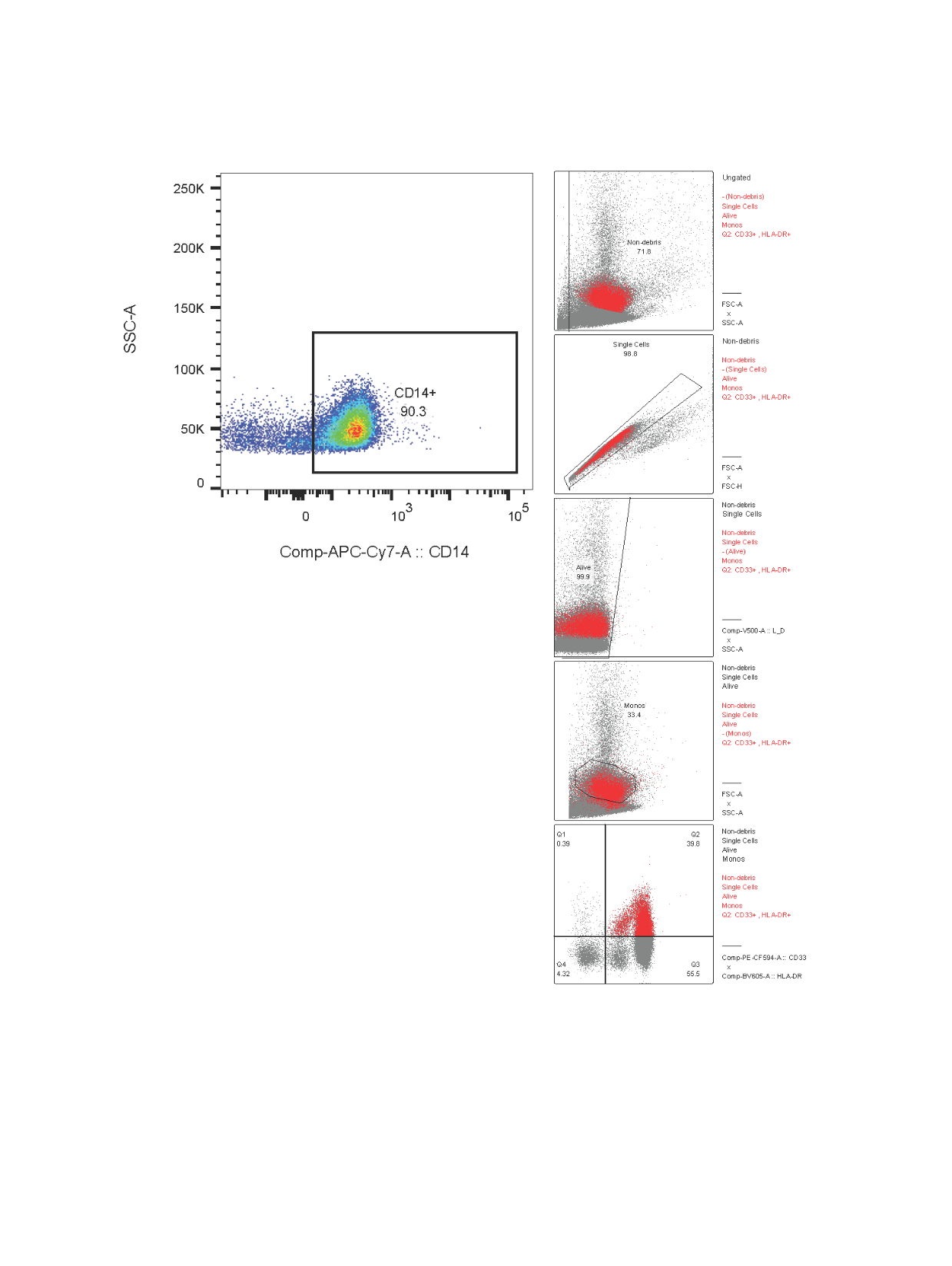


**Supplementary Figure 3e.** Example of Backgating of CD33^+^HLA-DR^+^ Myeloid Cells that Co-express CD14 in the Peripheral Blood of Participant C.


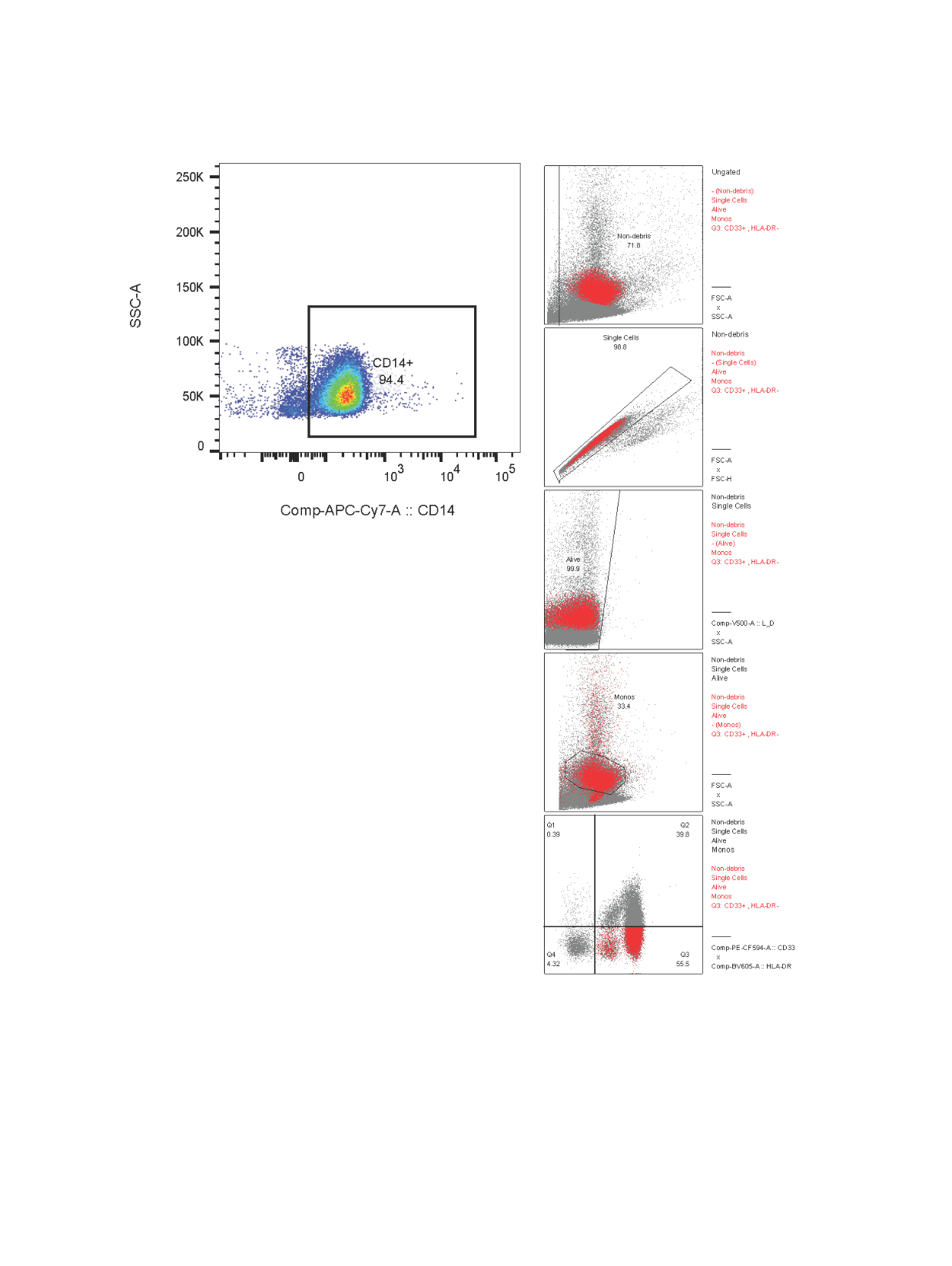


**Supplementary Figure 3f.** Example of Backgating of MDSC that Co-express CD14 in the Peripheral Blood of Participant C.


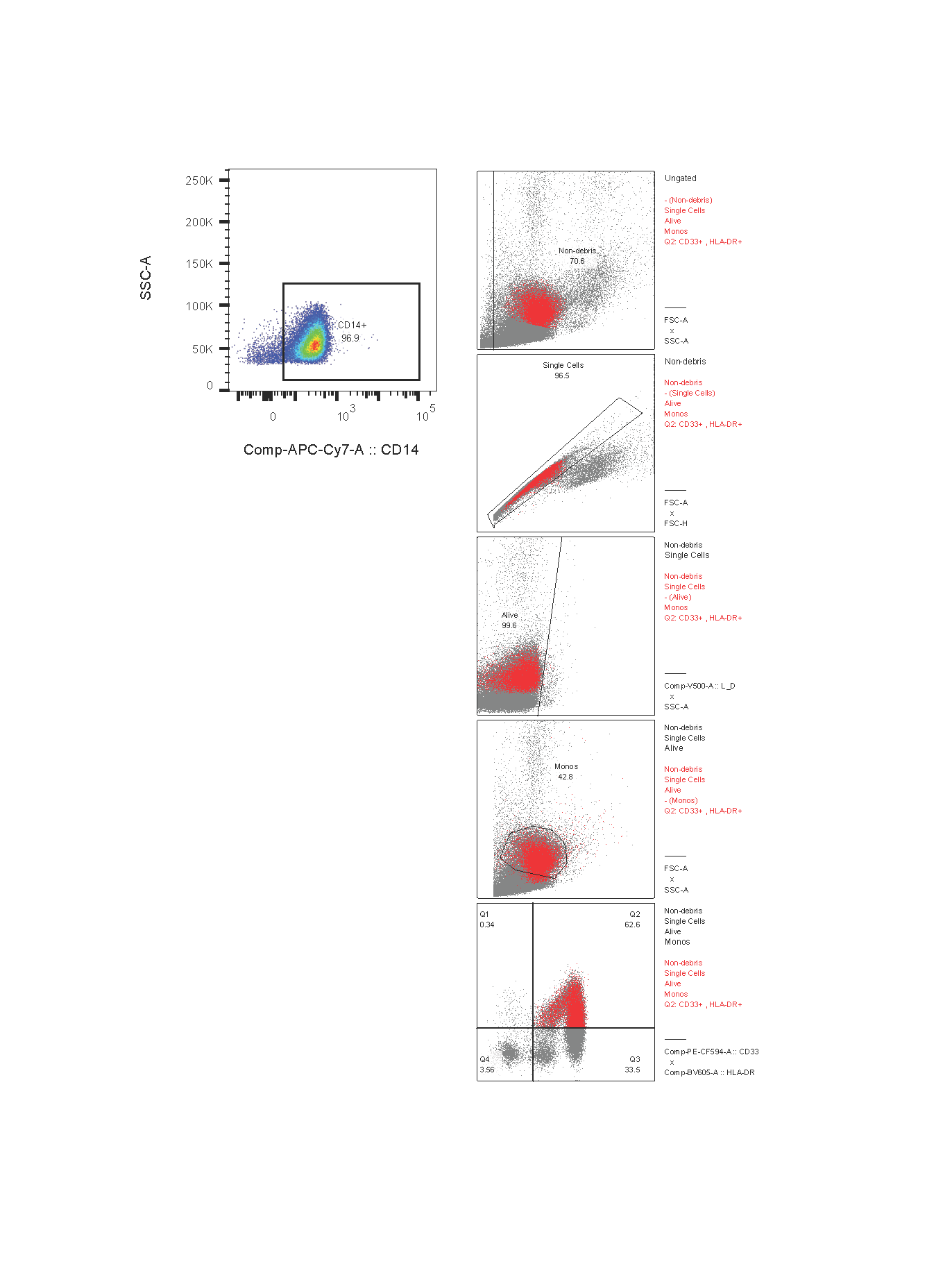


**Supplementary Figure 3g.** Example of Backgating of CD33^+^HLA-DR^+^ myeloid cells that Co-express CD14 in the Peripheral Blood of Participant D.


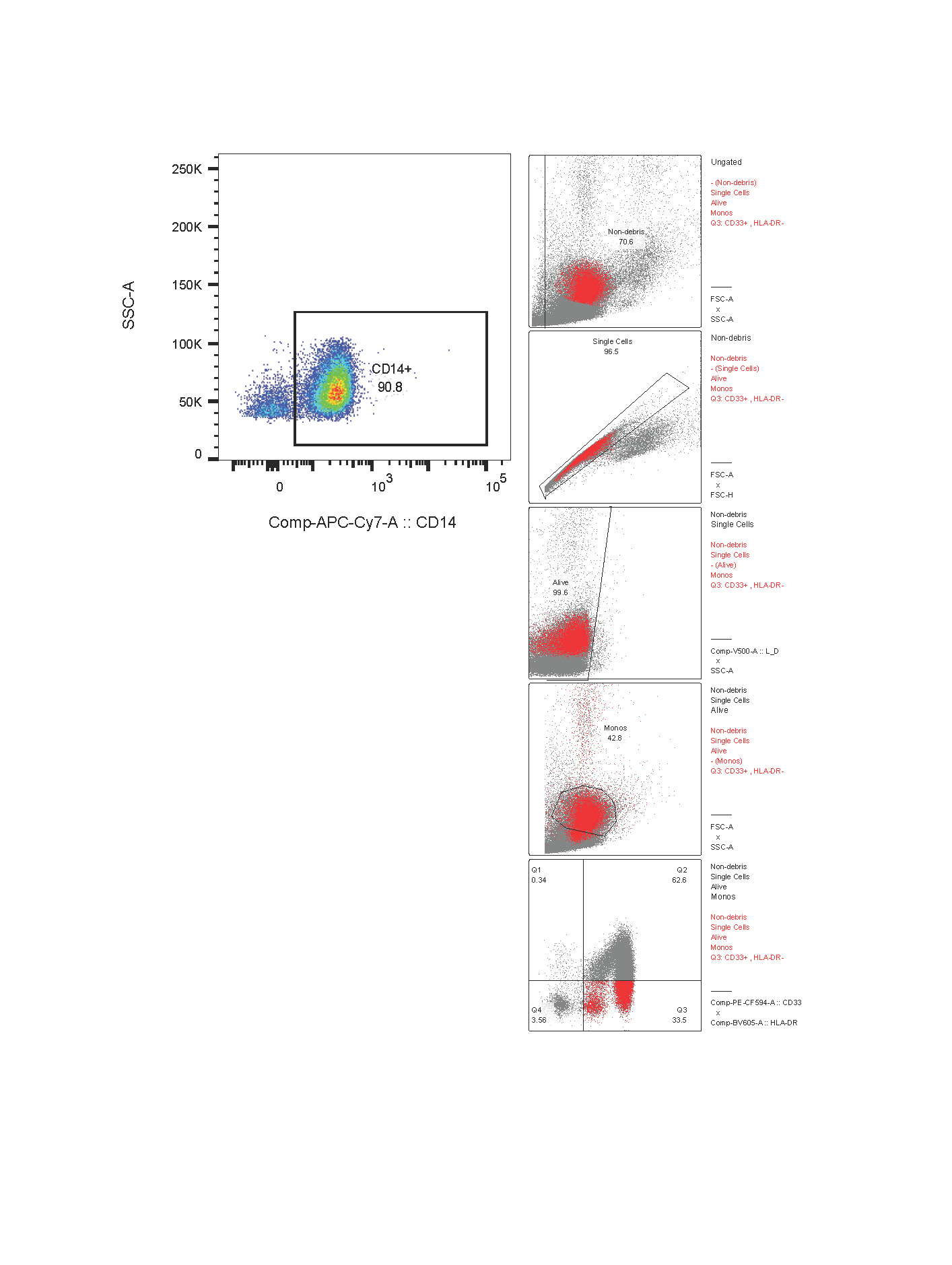


**Supplementary Figure 3h.** Example of Backgating of MDSC that Co-Express CD14 in the Peripheral Blood of Participant D.


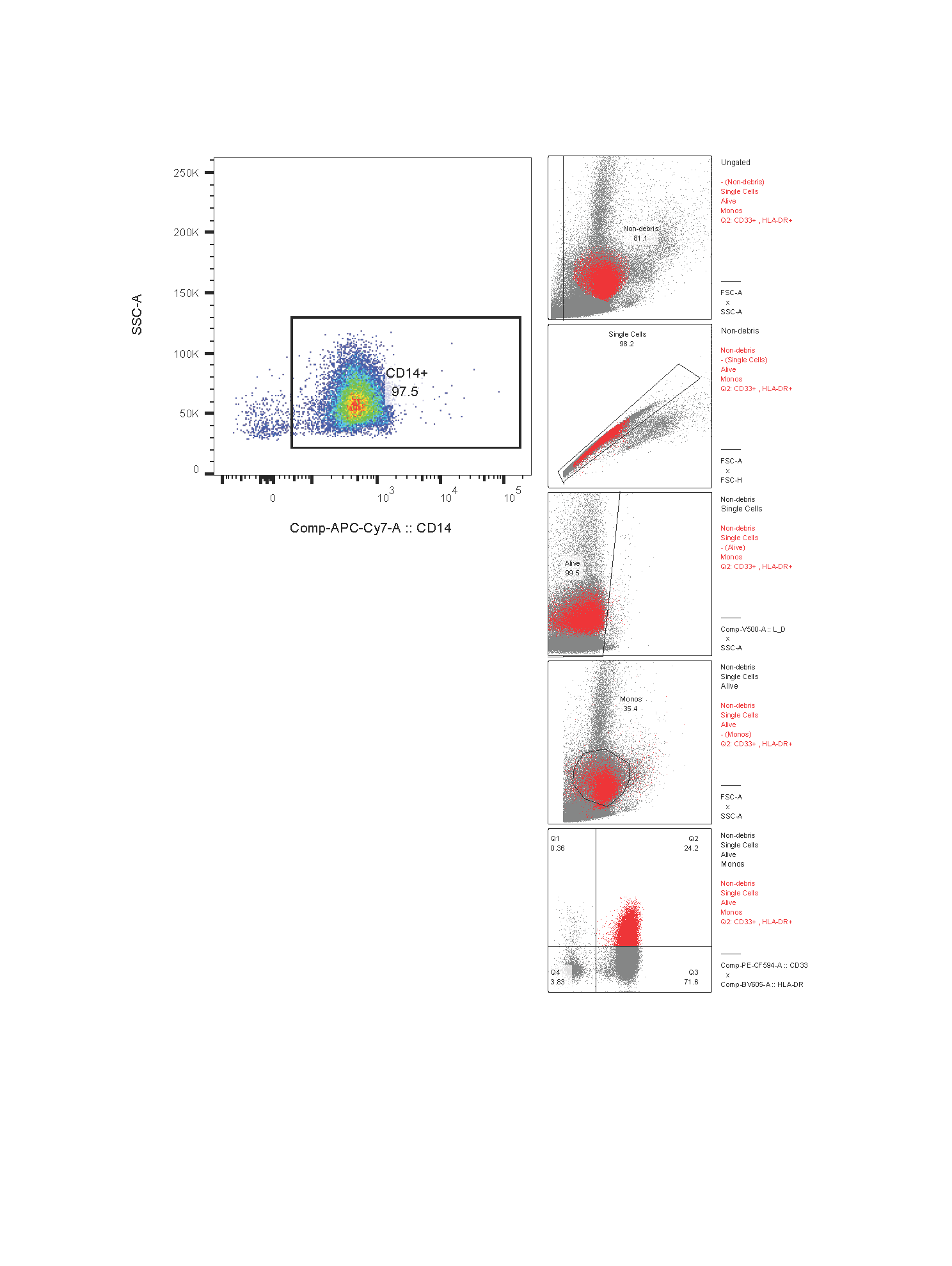


**Supplementary Figure 3i.** Example of Backgating of CD33^+^HLA-DR^+^ Myeloid Cells that also Co-express CD14 in the Peripheral Blood of Participant E.


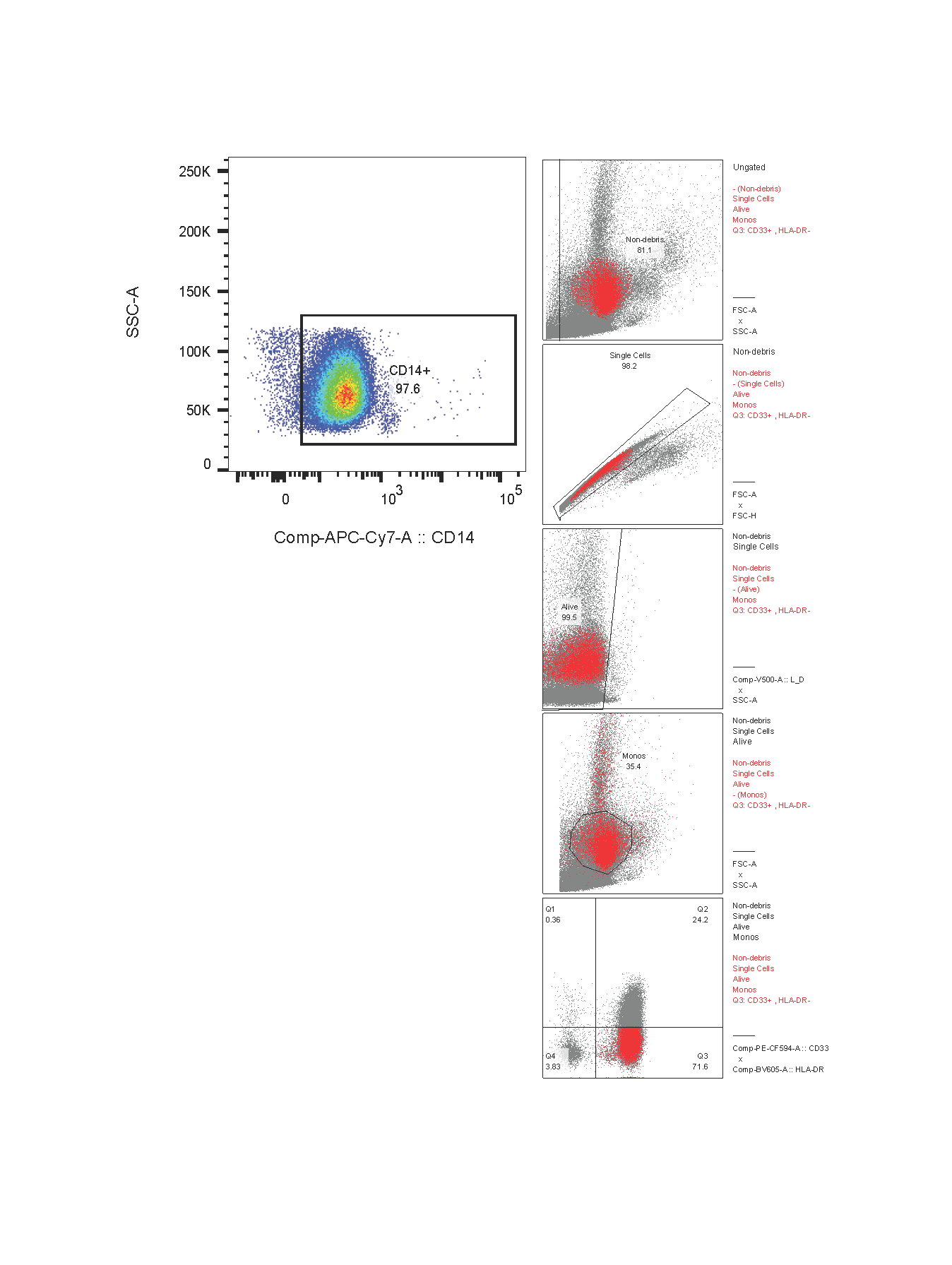


**Supplementary Figure 3j.** Example of Backgating of MDSC that Co-express CD14 in the Peripheral Blood of Participant E.


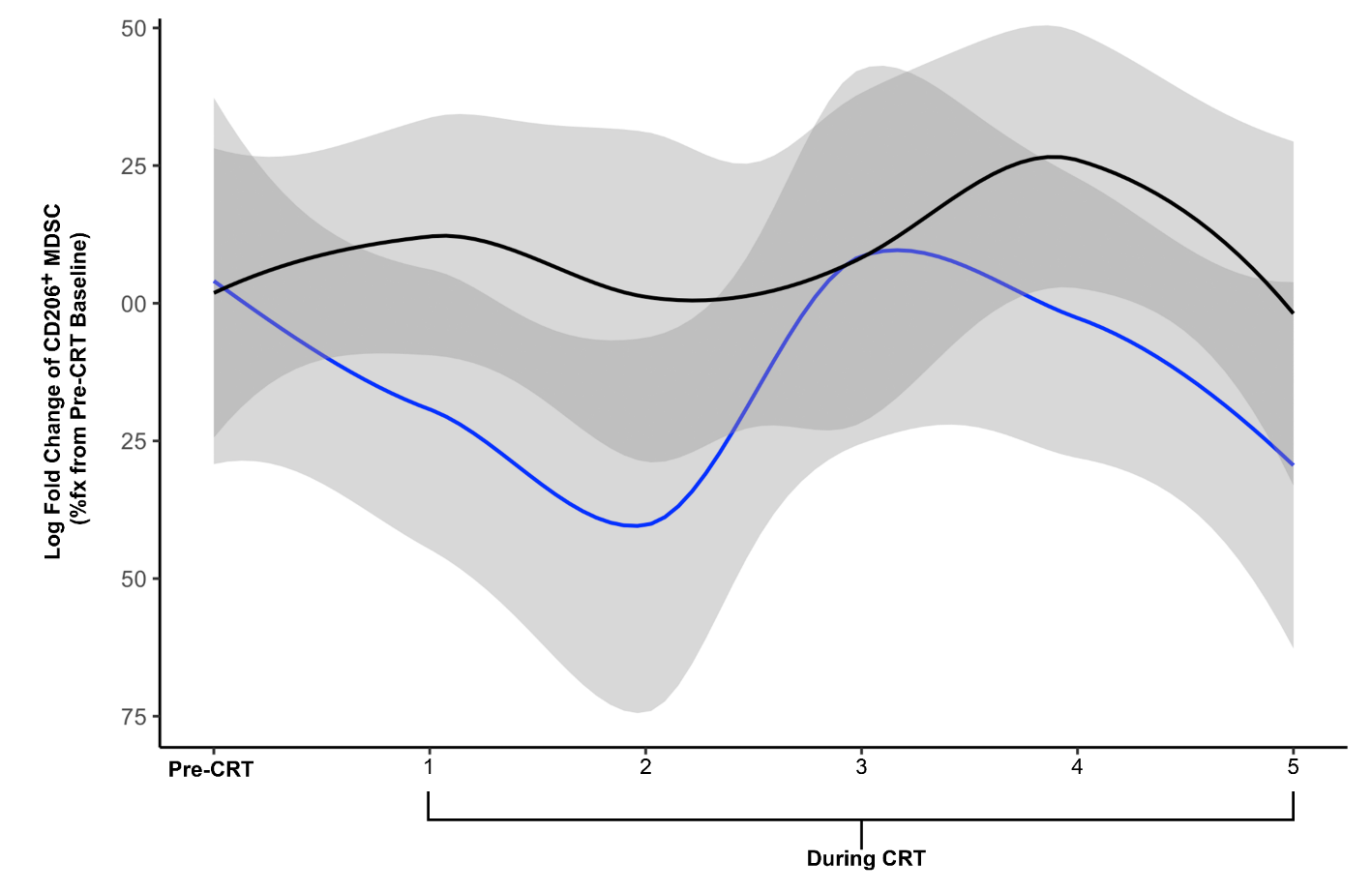

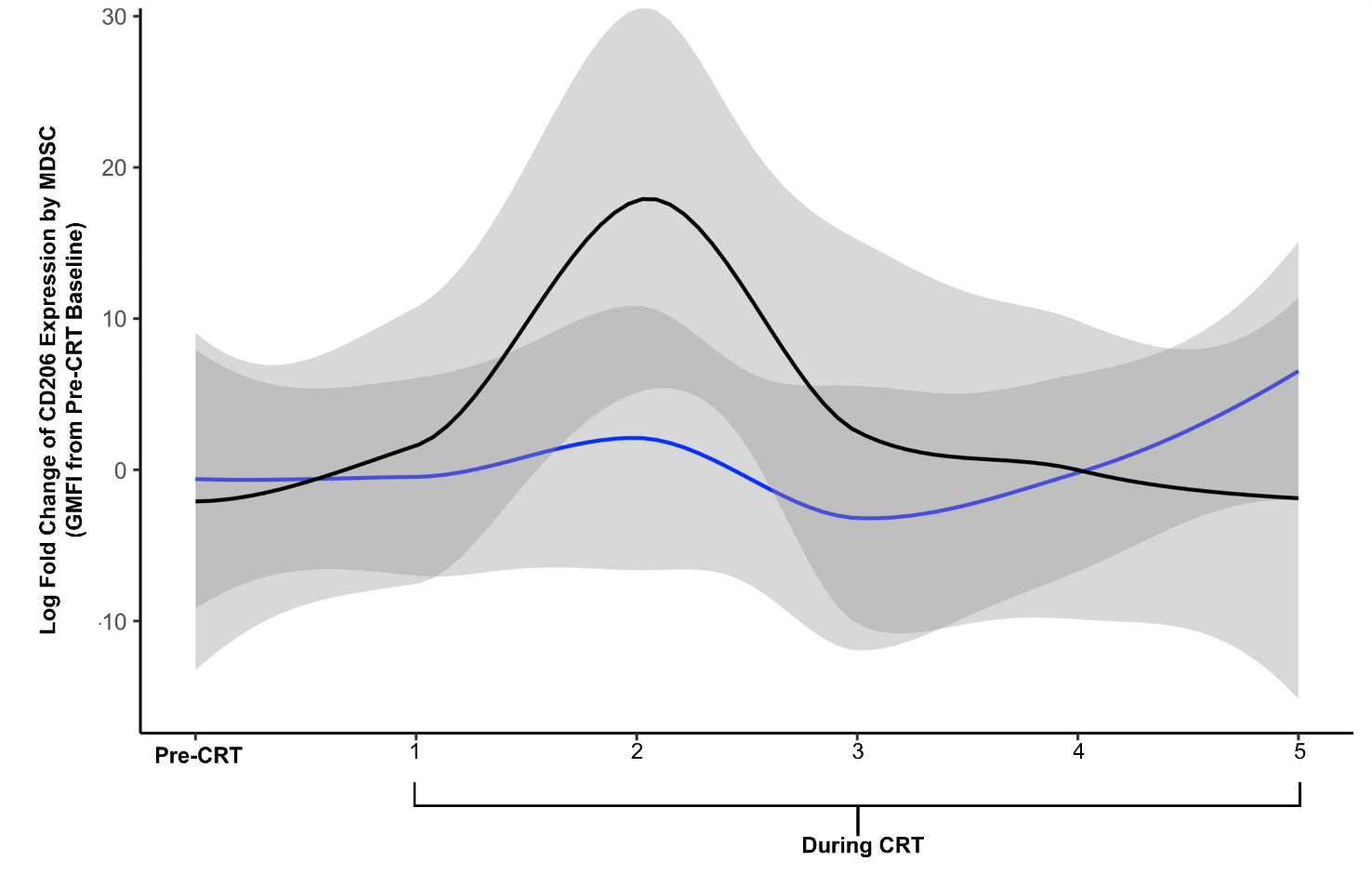


**Supplementary Figure 4a.** CD206 Expression by Flow Cytometry by MDSC: %fx (top) and GMFI (bottom).


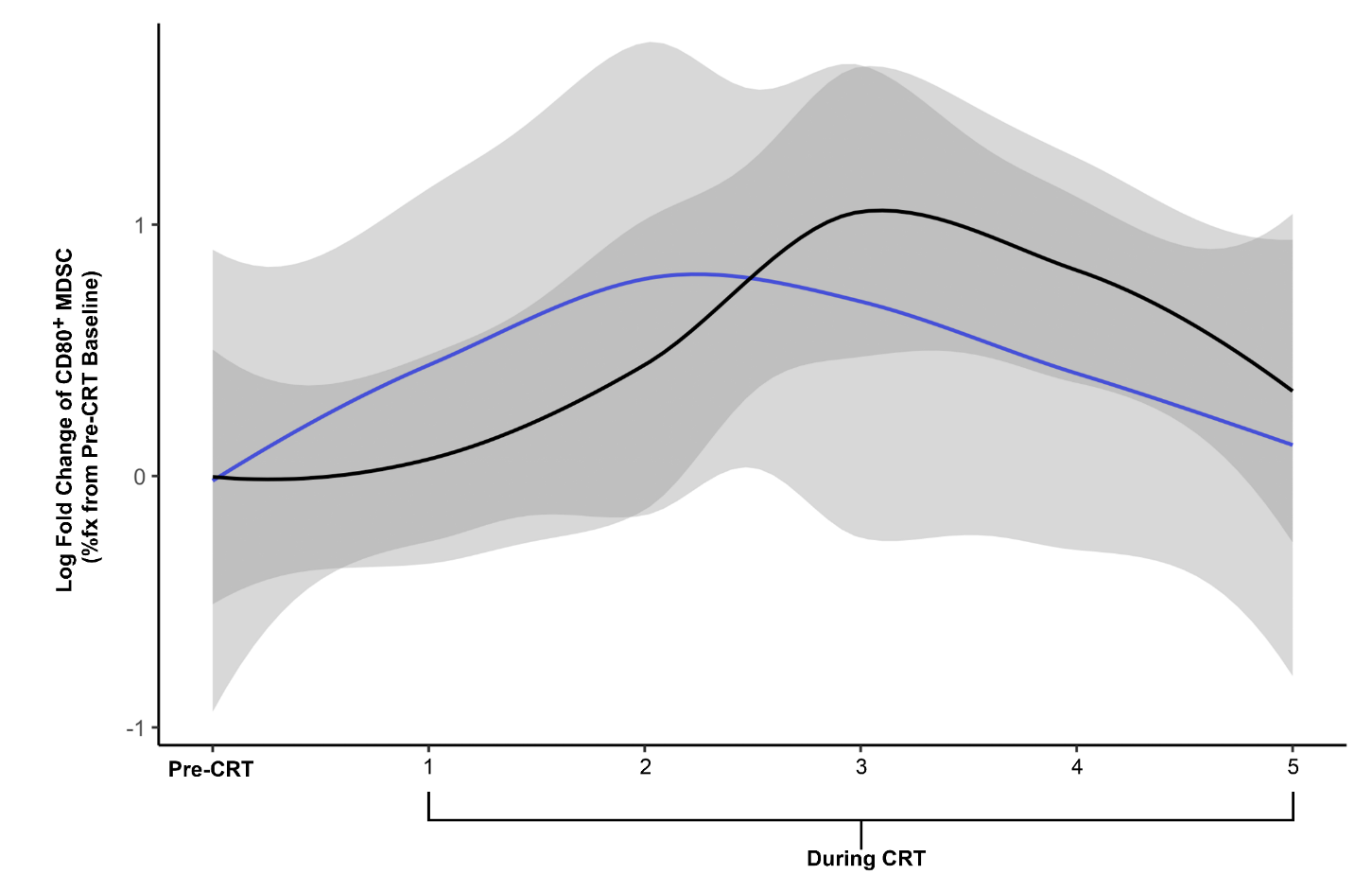


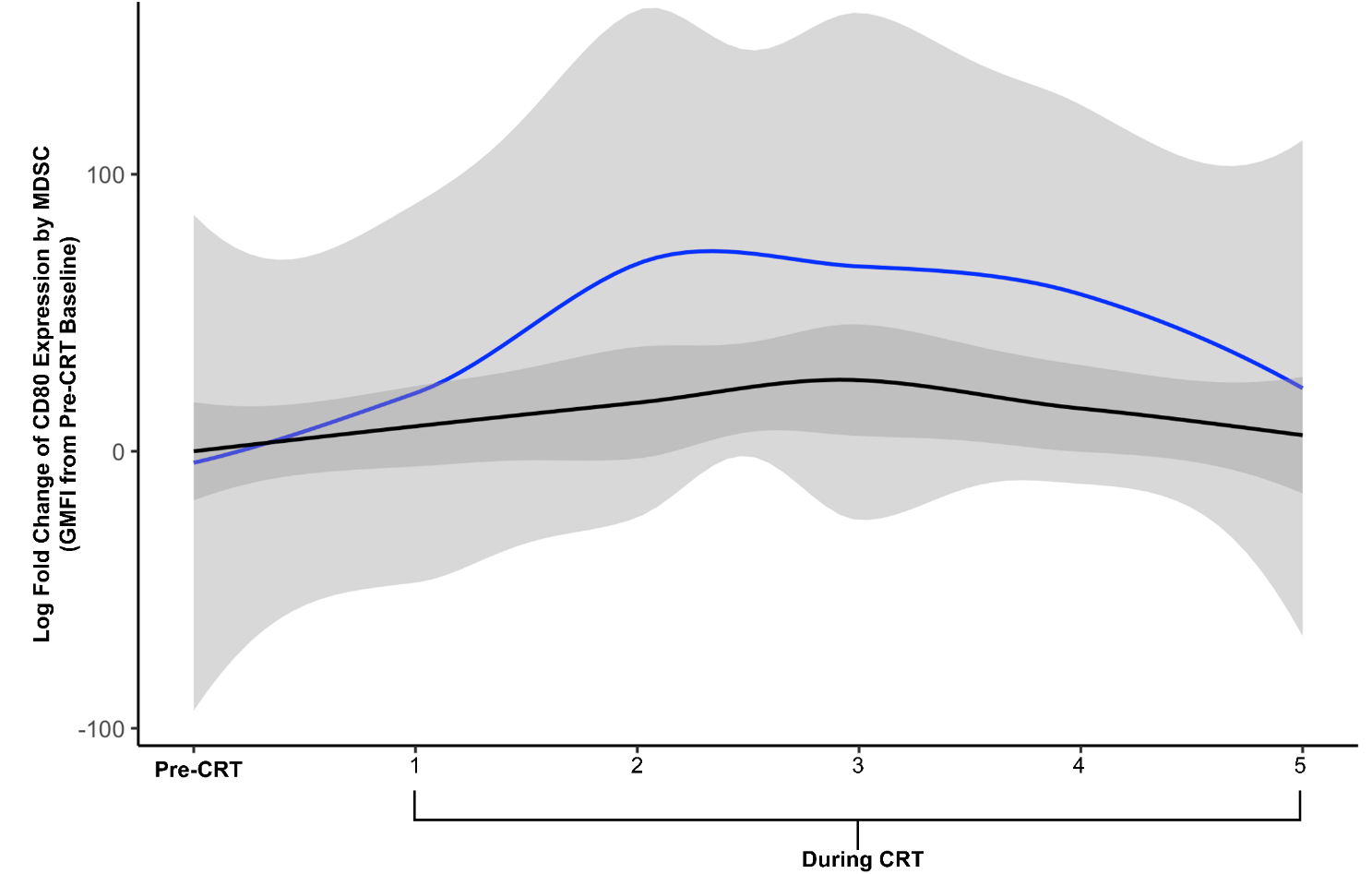


**Supplementary Figure 4b.** CD80 Expression by Flow Cytometry by MDSC: %fx (top) and GMFI (bottom).


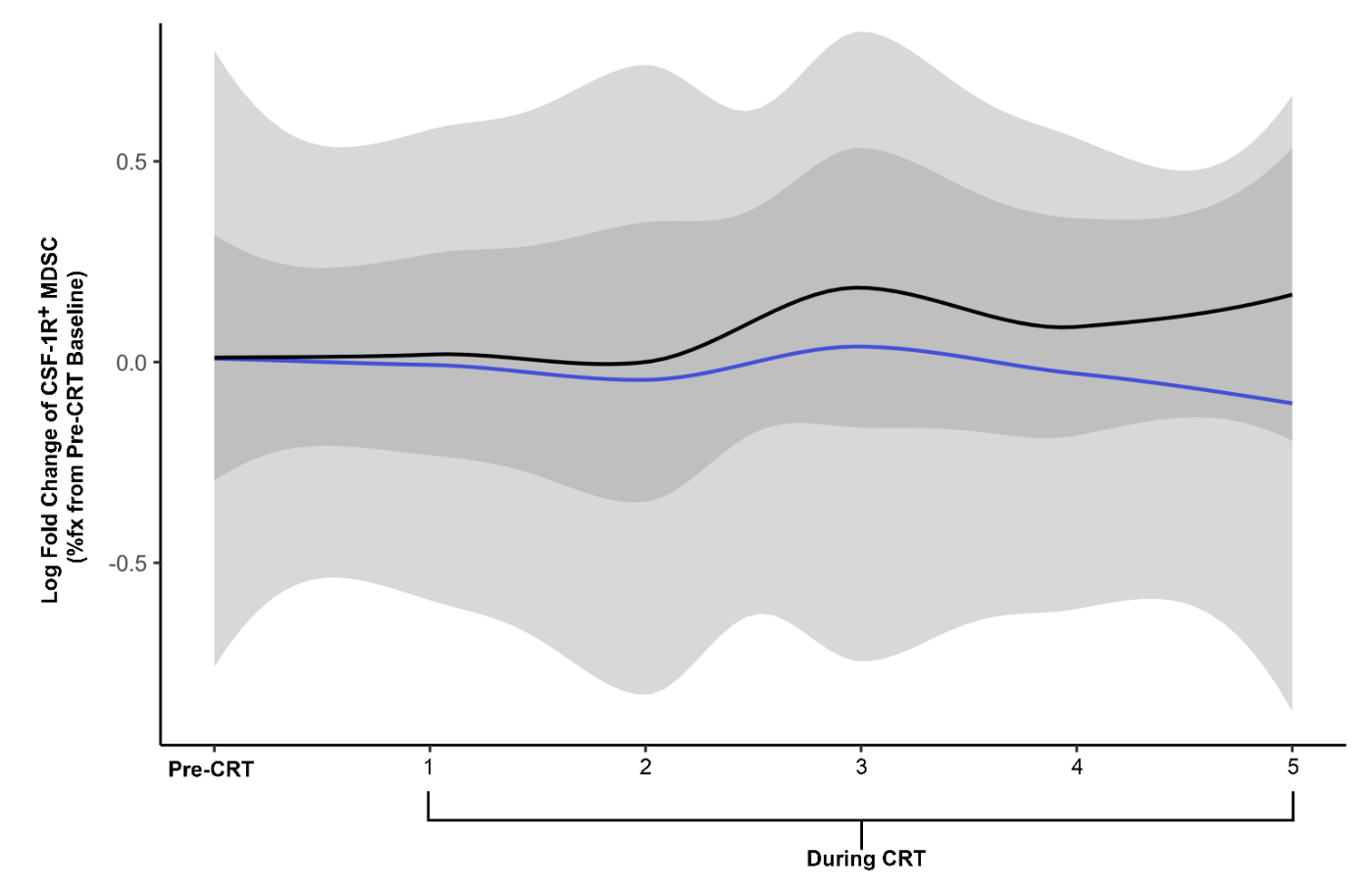


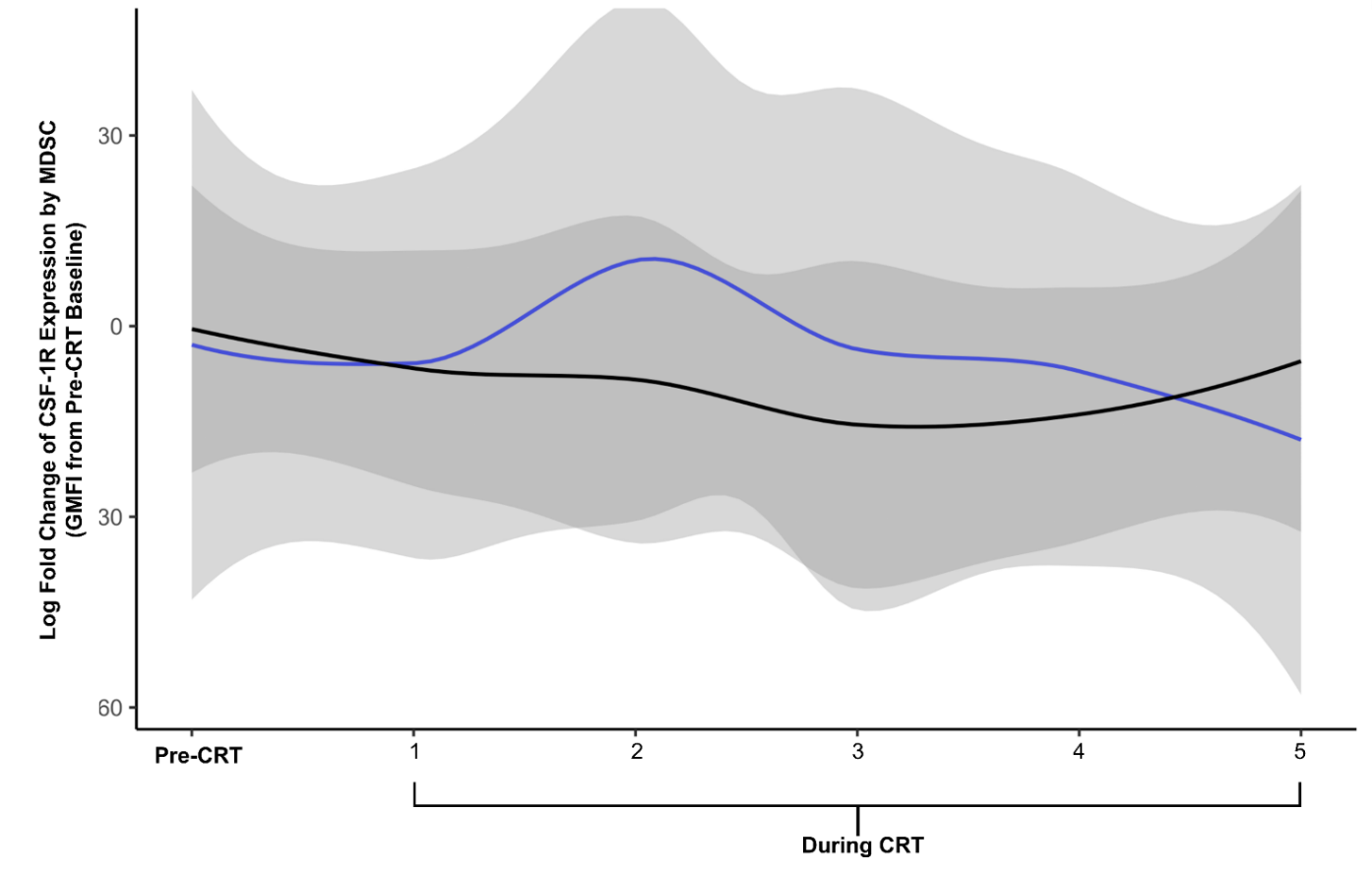


**Supplementary Figure 4c.** CSF1-R Expression by Flow Cytometry by MDSC: %fx (top) and GMFI (bottom).


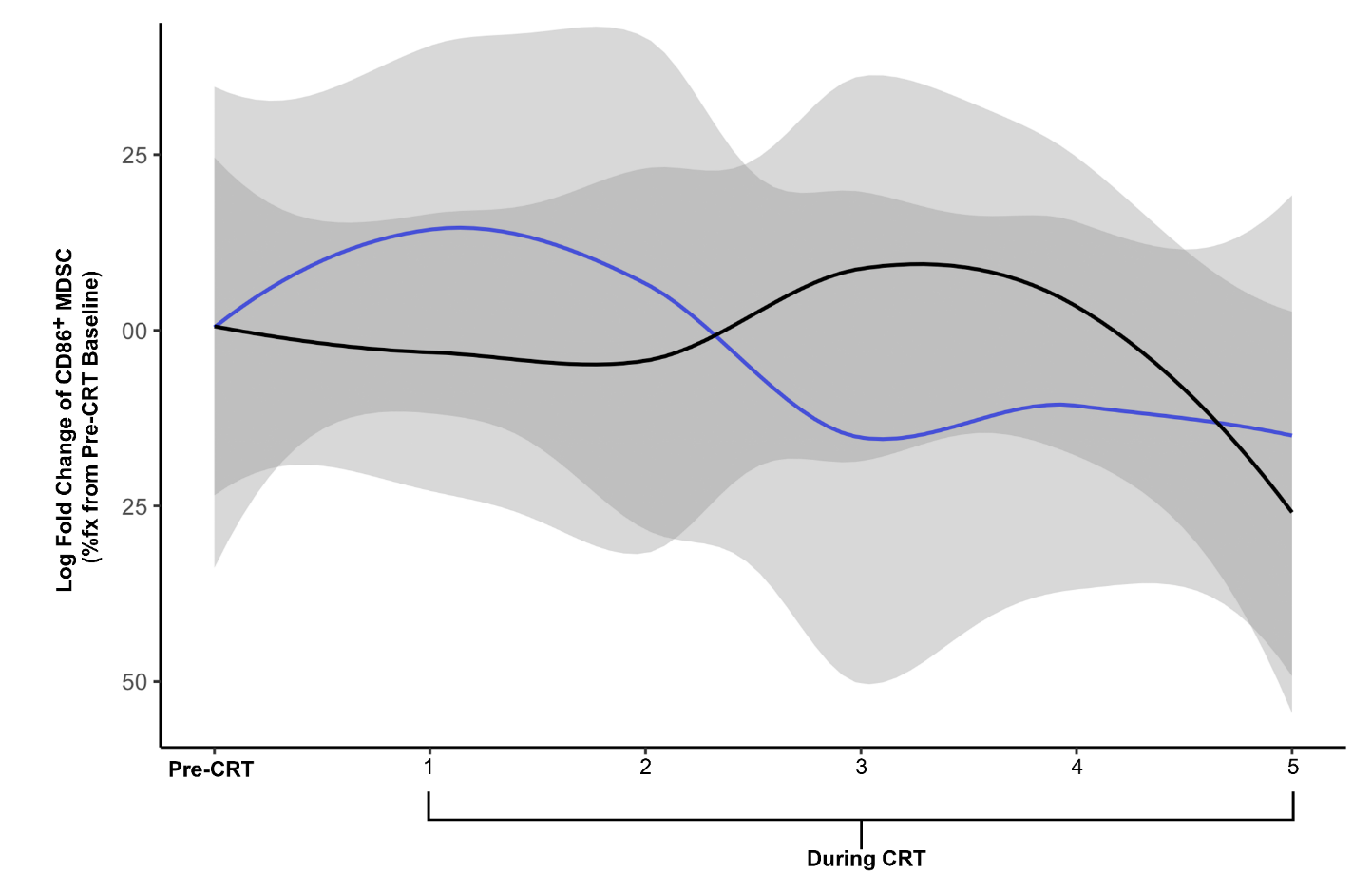


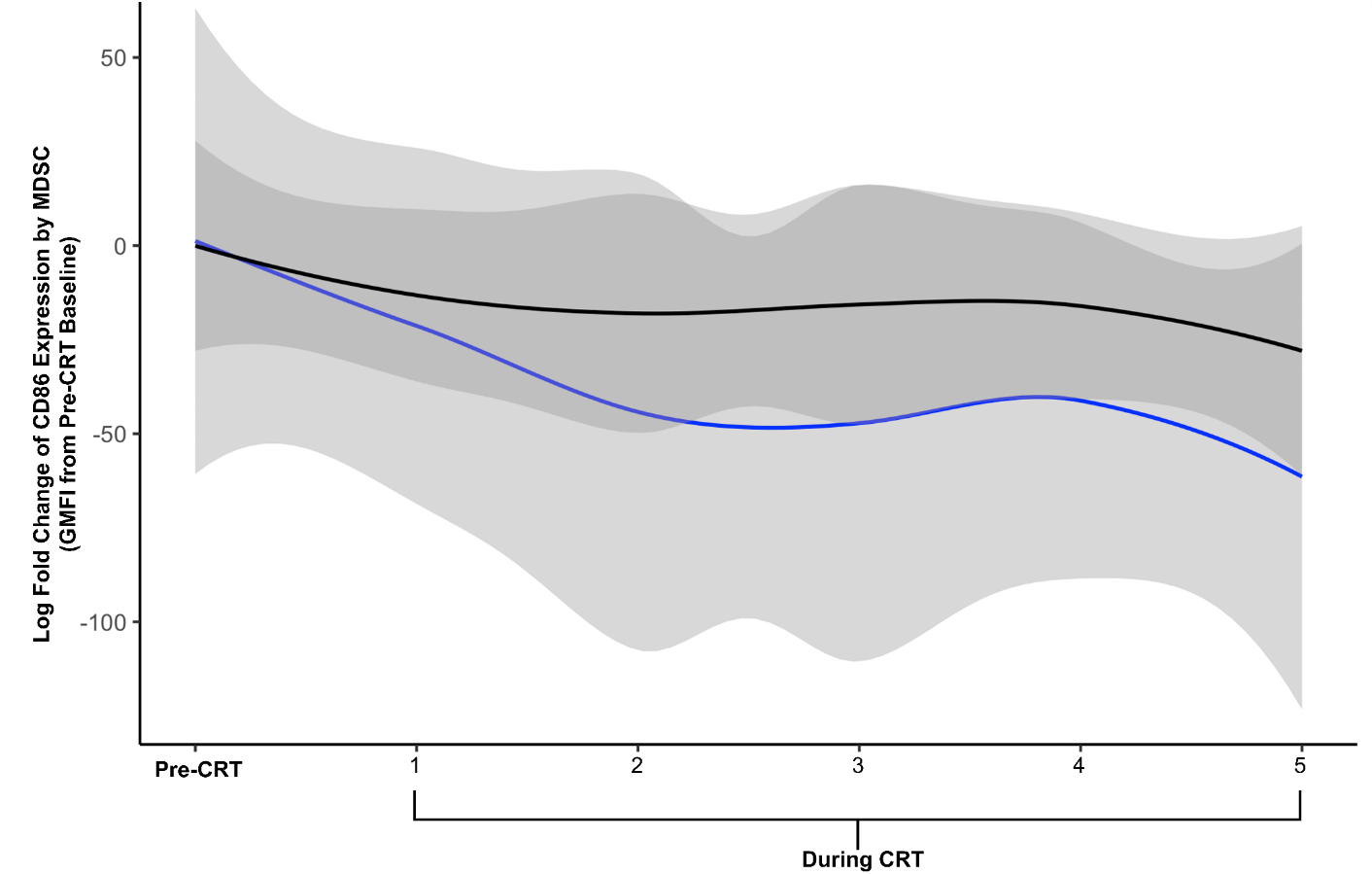


**Supplementary Figure 4d.** CD86 Expression by Flow Cytometry by MDSC: %fx (top) and GMFI (bottom).


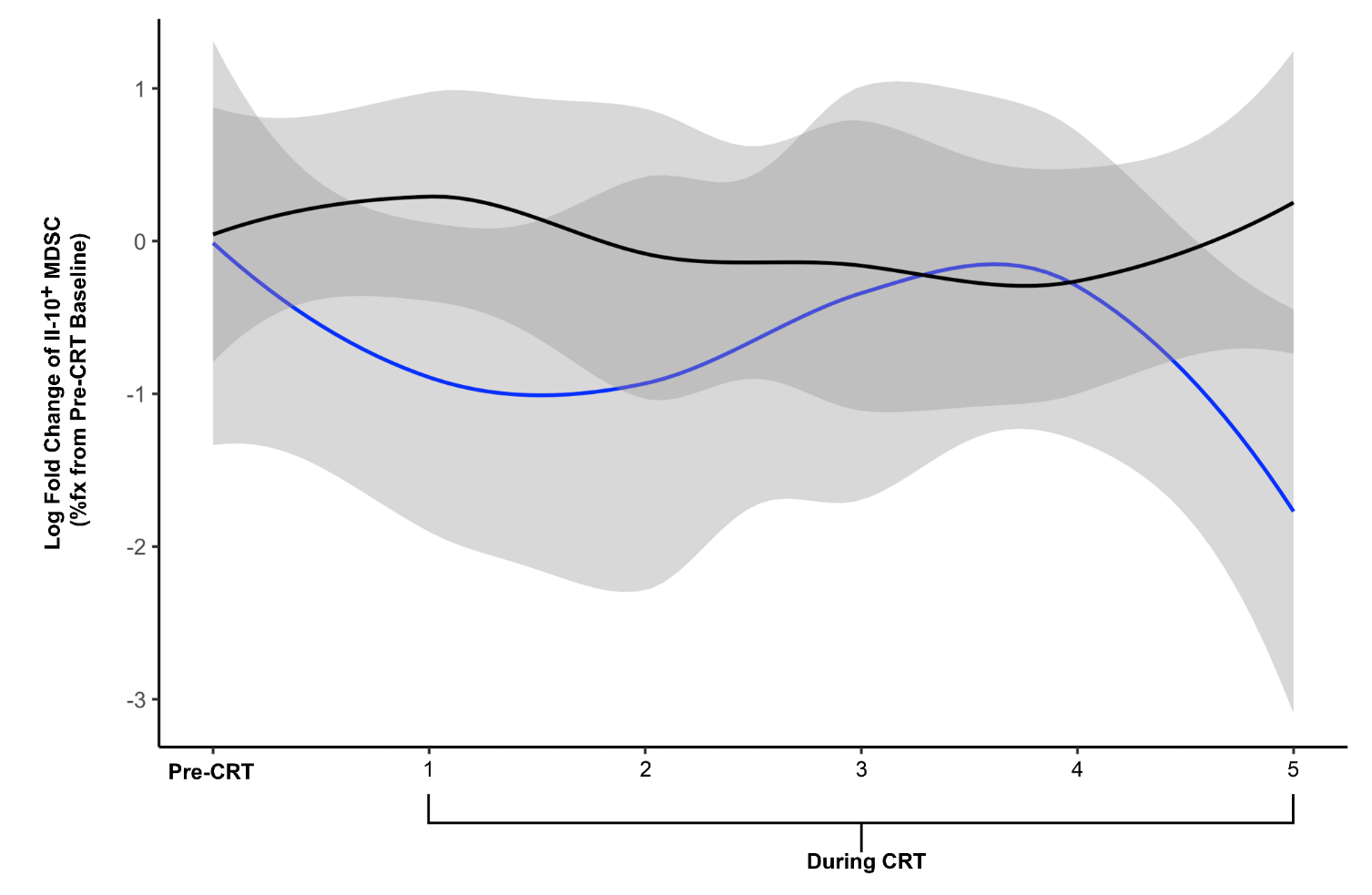


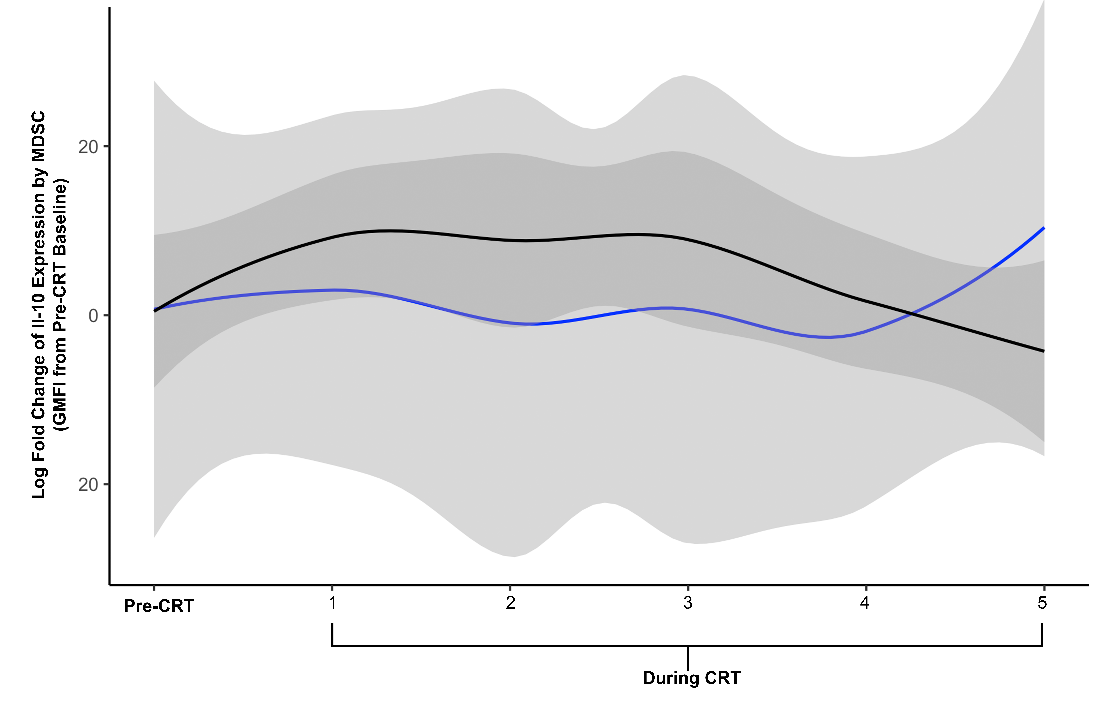


**Supplementary Figure 4e.** IL-10 Expression by Flow cytometry by MDSC: %fx (top) and GMFI (bottom).


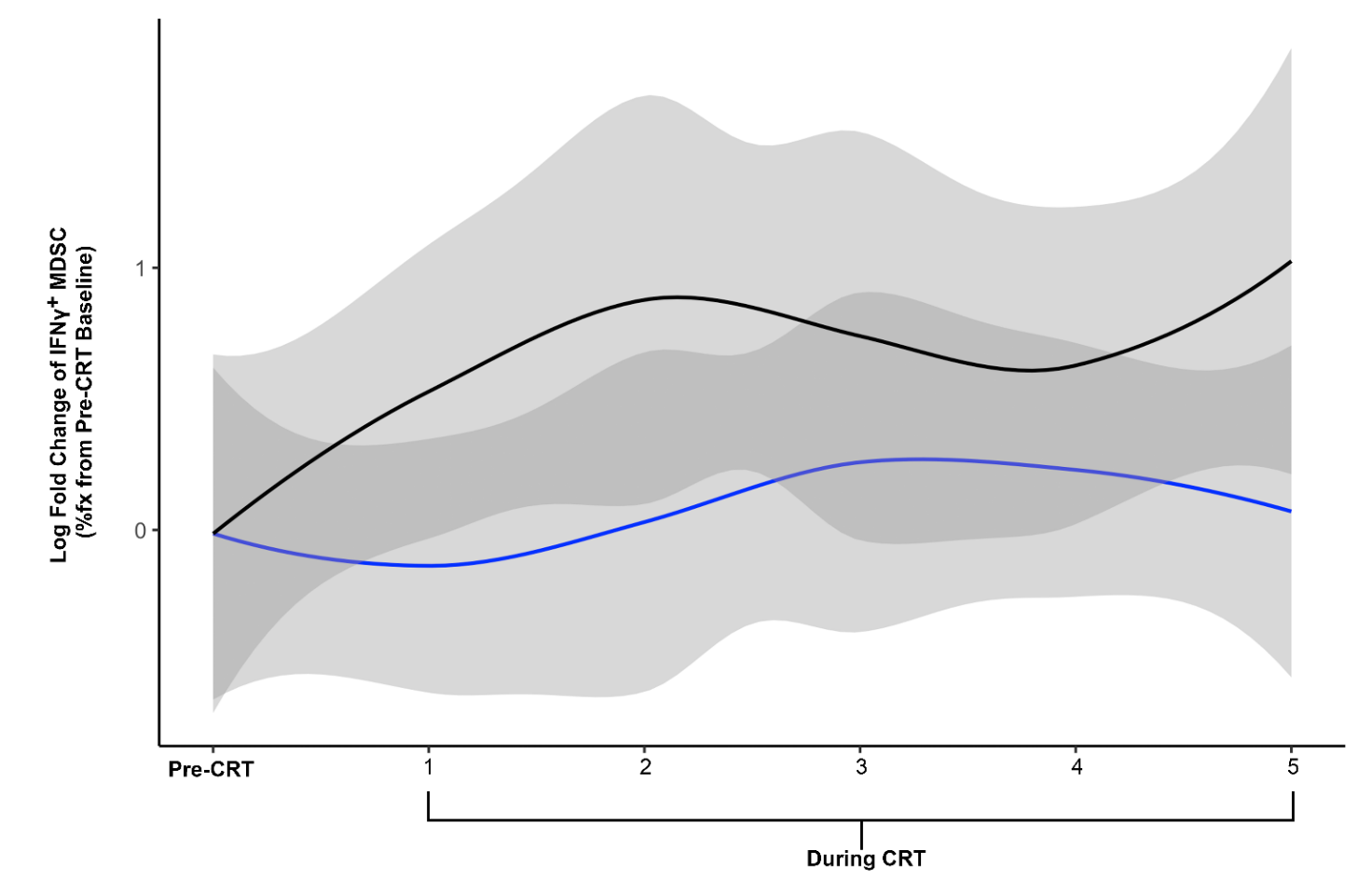


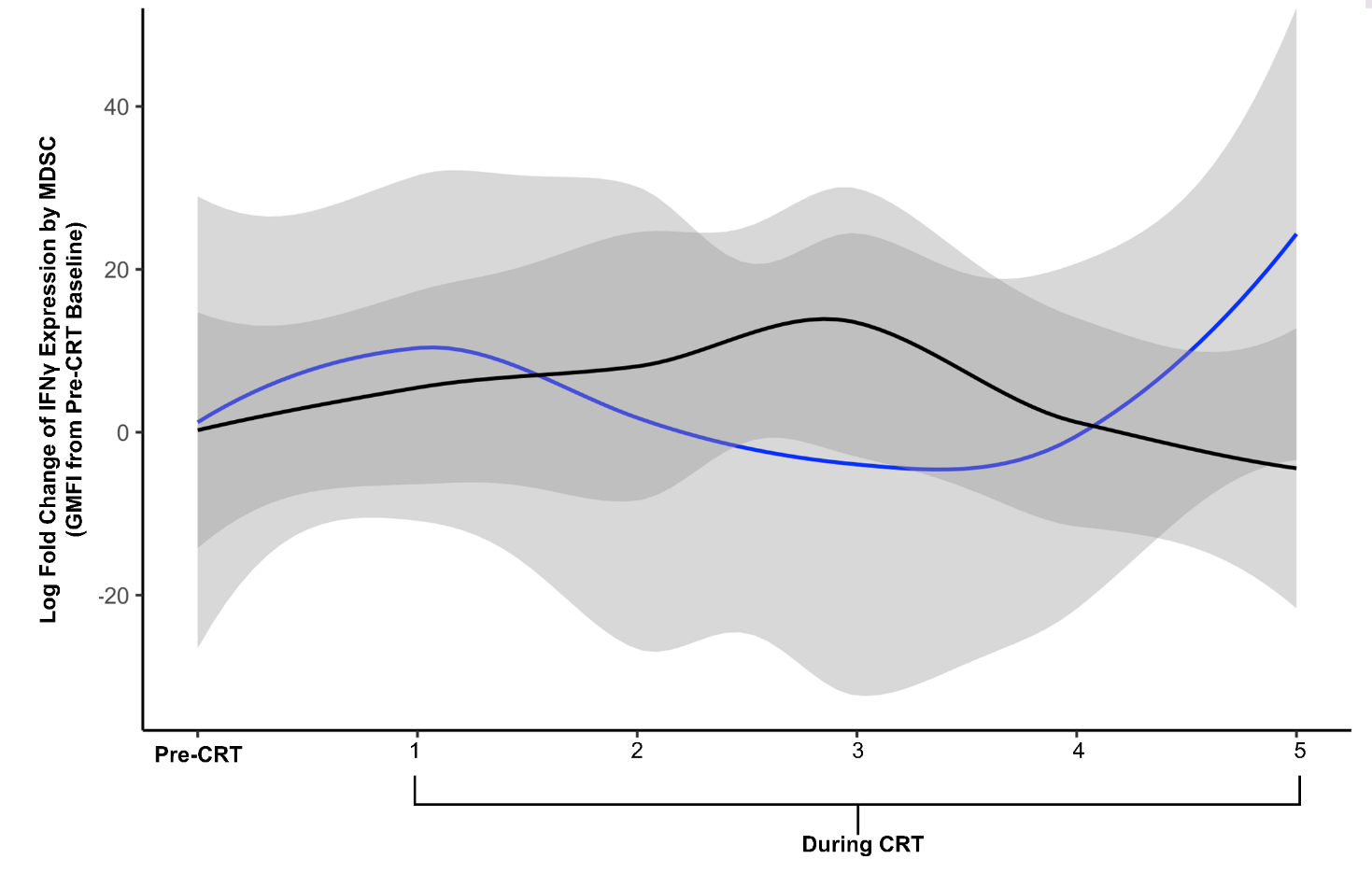


**Supplementary Figure 4f.** IFN$\gamma$ Expression by Flow Cytometry by MDSC: %fx (top) and GMFI (bottom)


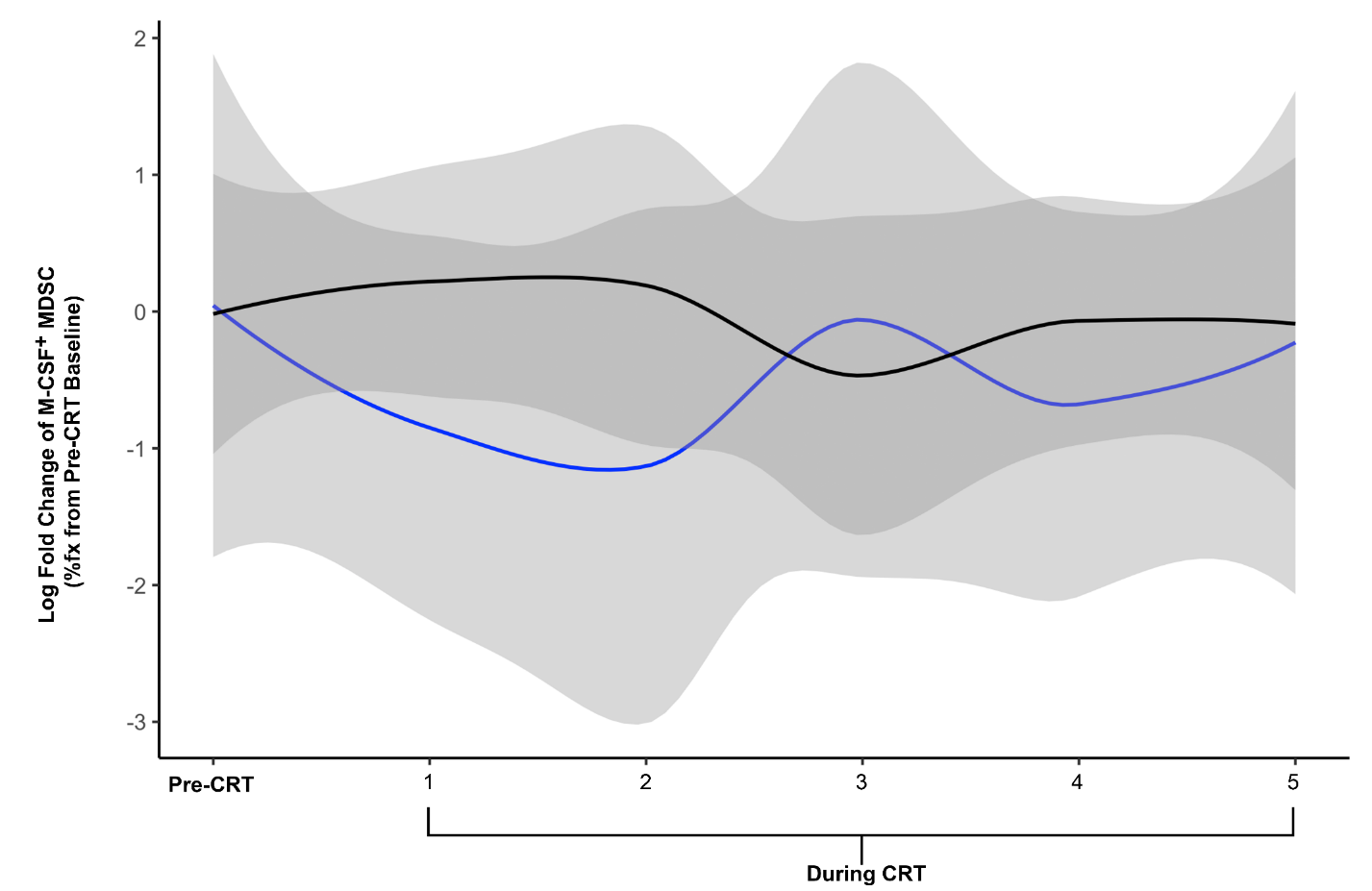


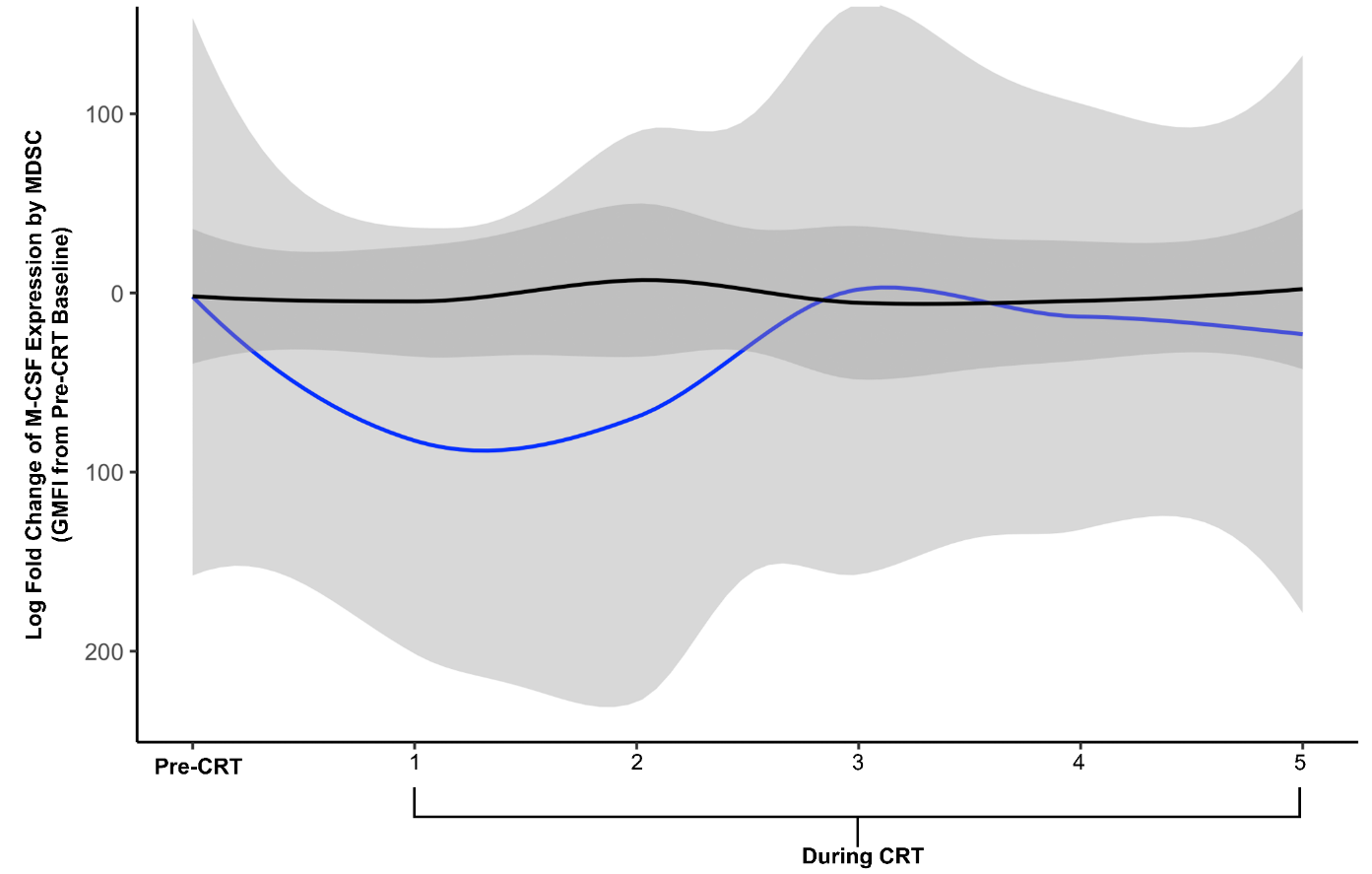


**Supplementary Figure 4g.** M-CSF Expression by Flow Cytometry by MDSC: %fx (top) and GMFI (bottom).


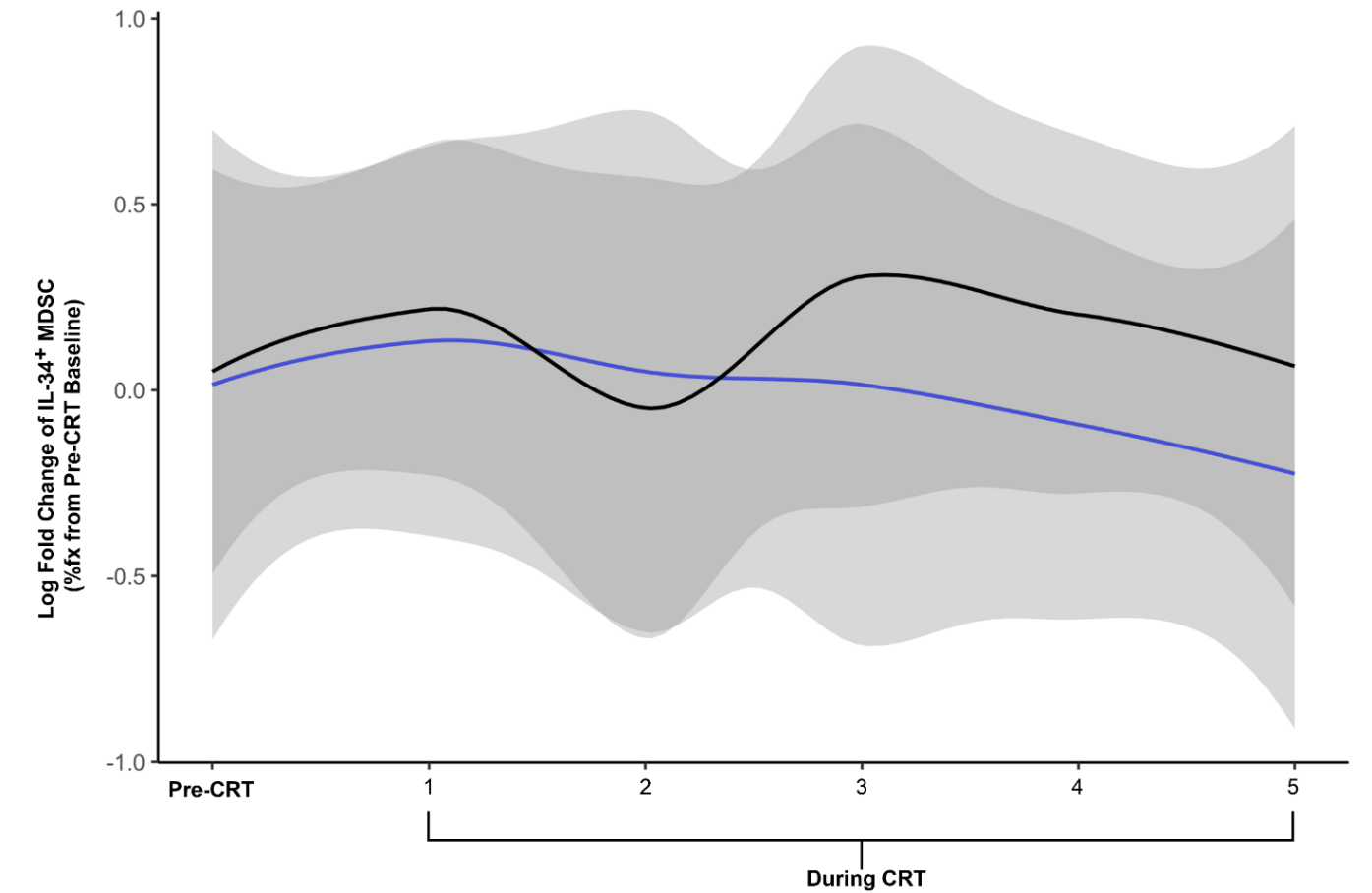


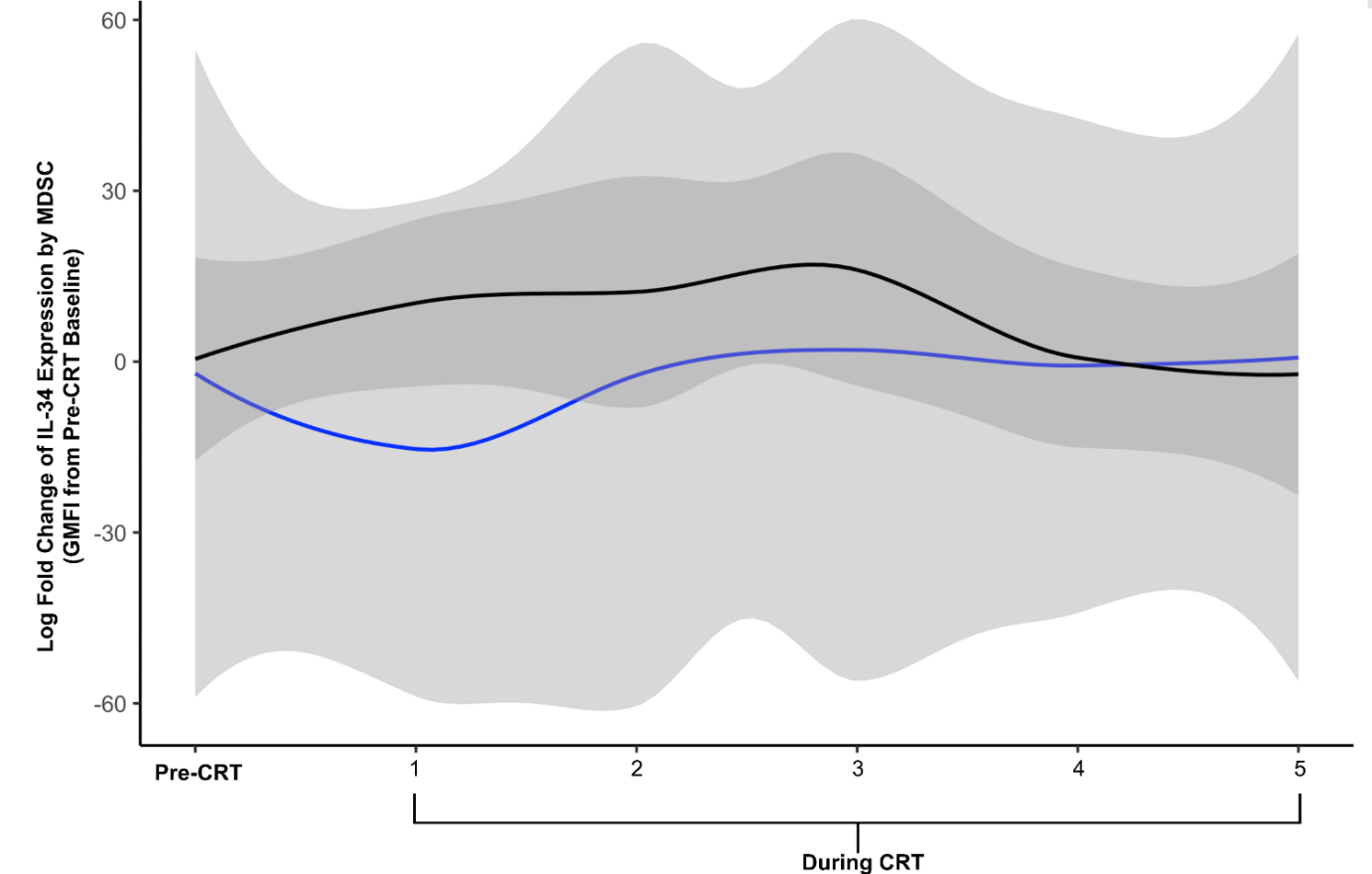


**Supplementary Figure 4h.** IL-34 Expression by Flow Cytometry by MDSC: %fx (top) and GMFI (bottom).


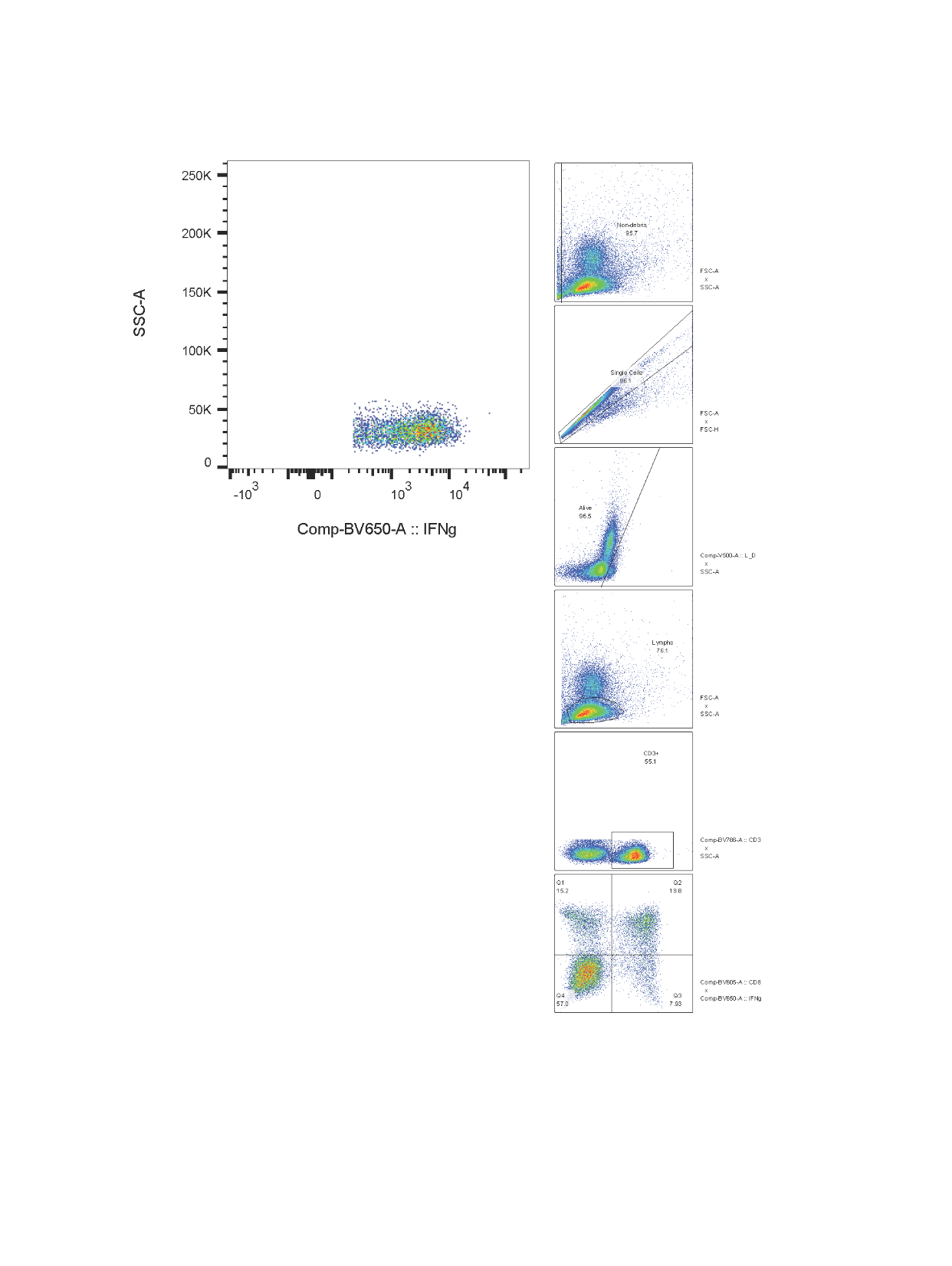


**Supplementary Figure 5a.** Example of Backgating of CD8^+^ T lymphocytes that Co-express IFN$\gamma$ in the Peripheral Blood of Participant A.


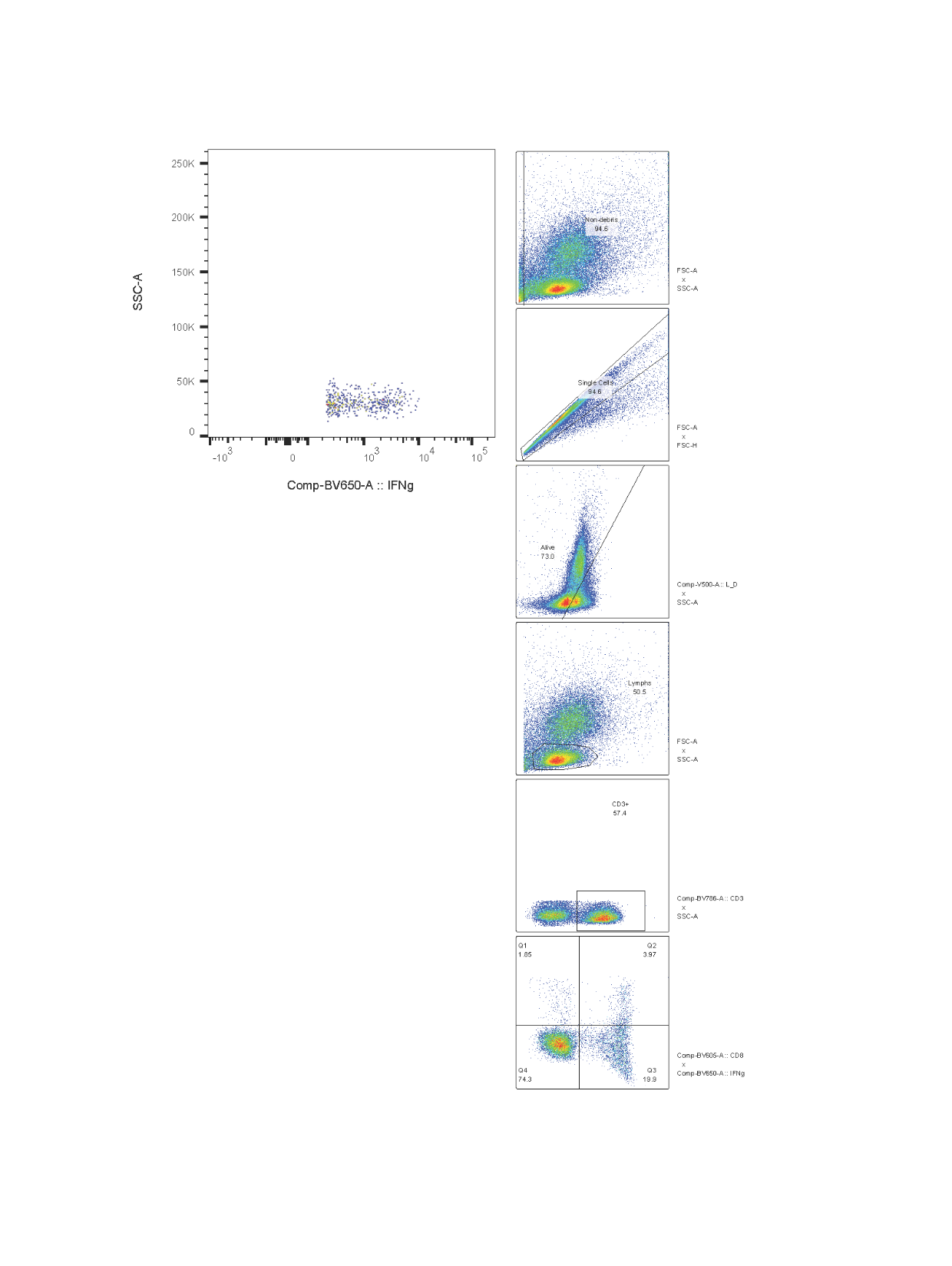


**Supplementary Figure 5b.** Example Gating Strategy for CD8^+^ T Lymphocytes that Co-express IFN$\gamma$ in the Peripheral Blood of Participant B.


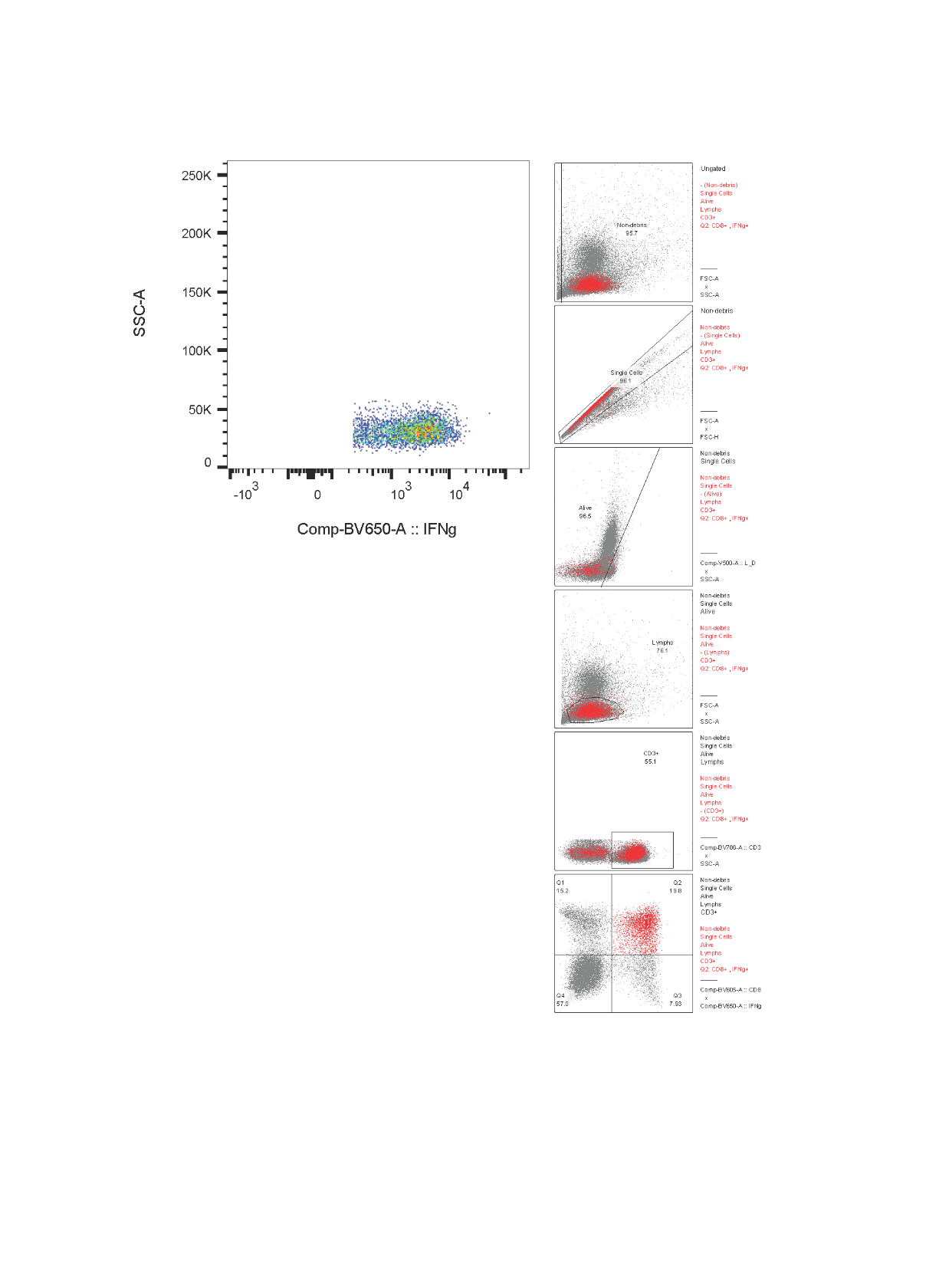


**Supplementary Figure 6a.** Example of Backgating of CD8^+^ T Lymphocytes that Co-express IFN$\gamma$ in the Peripheral Blood of Participant C.


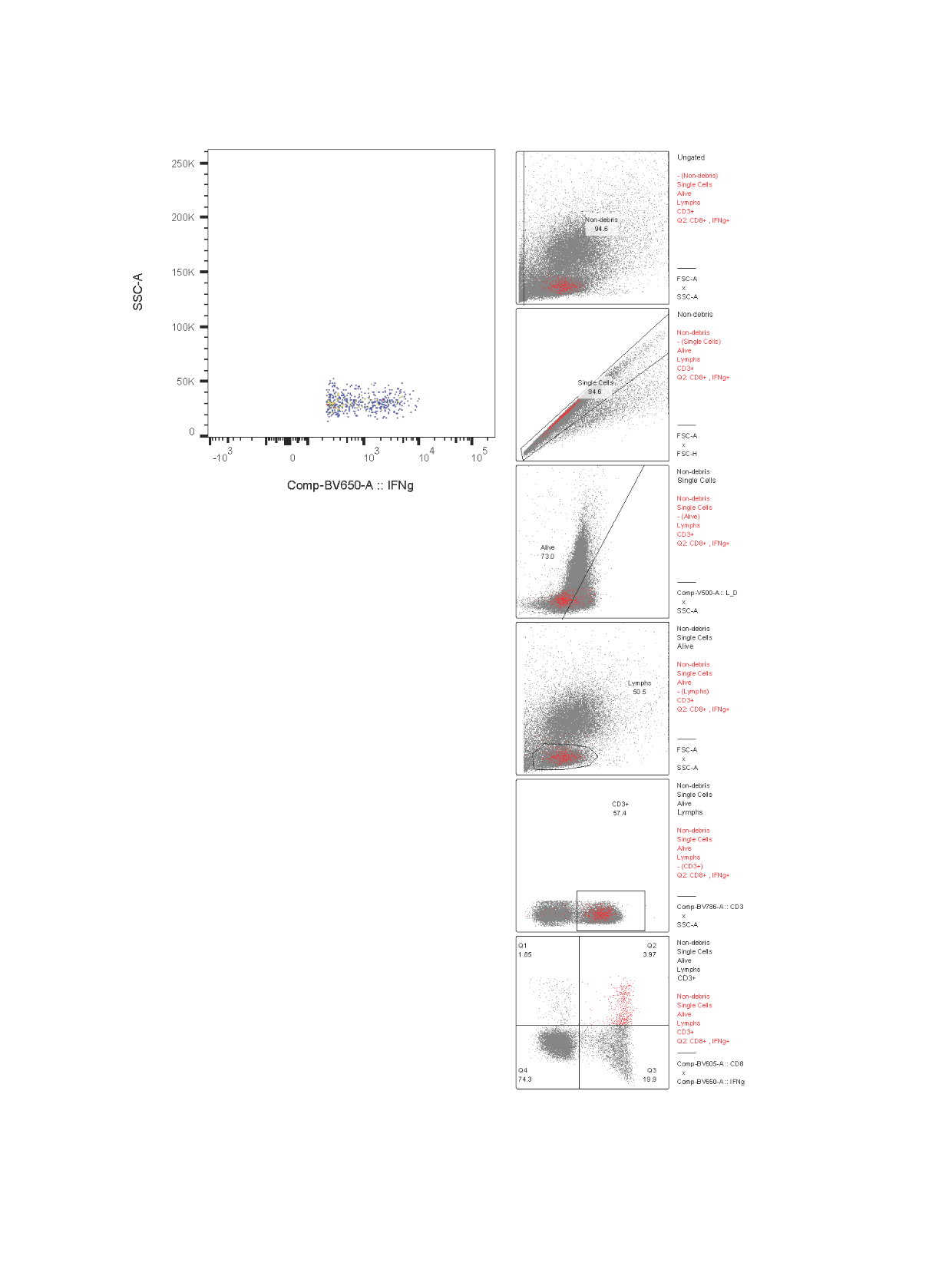


**Supplementary Figure 6b.** Example of Backgating of CD8^+^ T Lymphocytes that Co-express IFN$\gamma$ in the Peripheral Blood of Participant D.


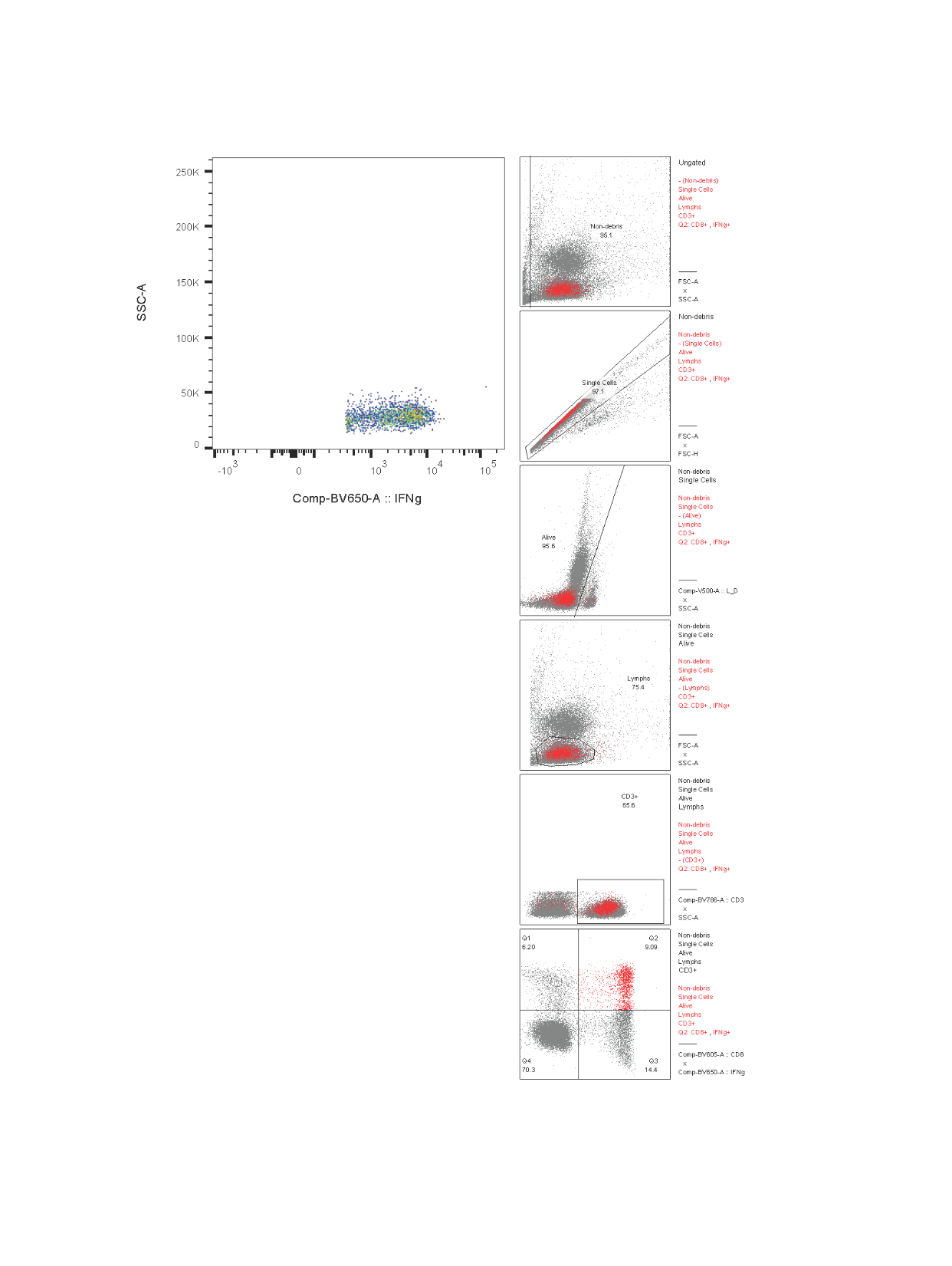


**Supplementary Figure 6c.** Example of Backgating of CD8^+^ T Lymphocytes that Co-express IFN$\gamma$ in the Peripheral Blood of Participant E.


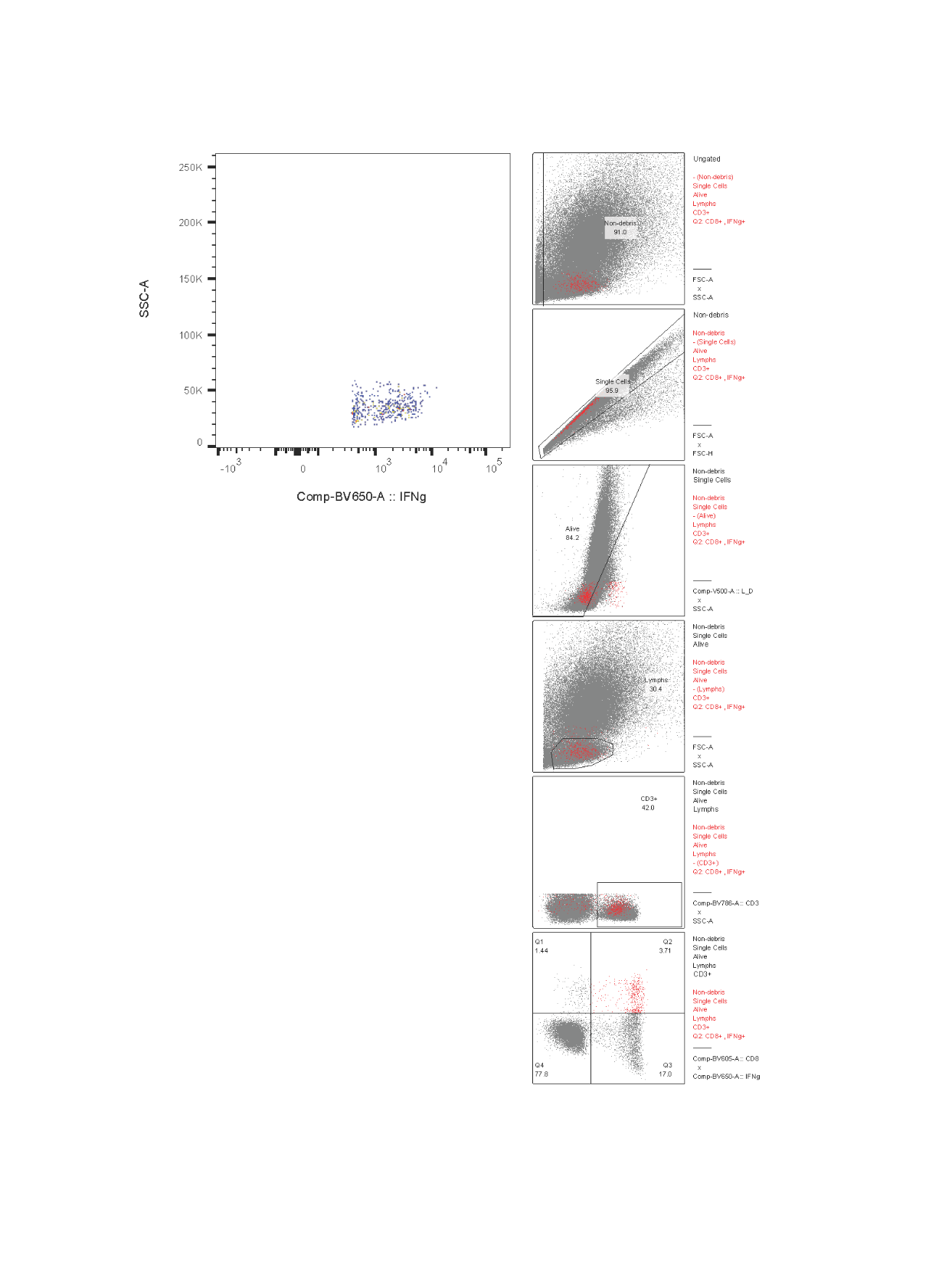


**Supplementary Figure 6d.** Example of Backgating of CD8^+^ T lymphocytes that Co-express IFN$\gamma$ in the Peripheral Blood of Participant D.


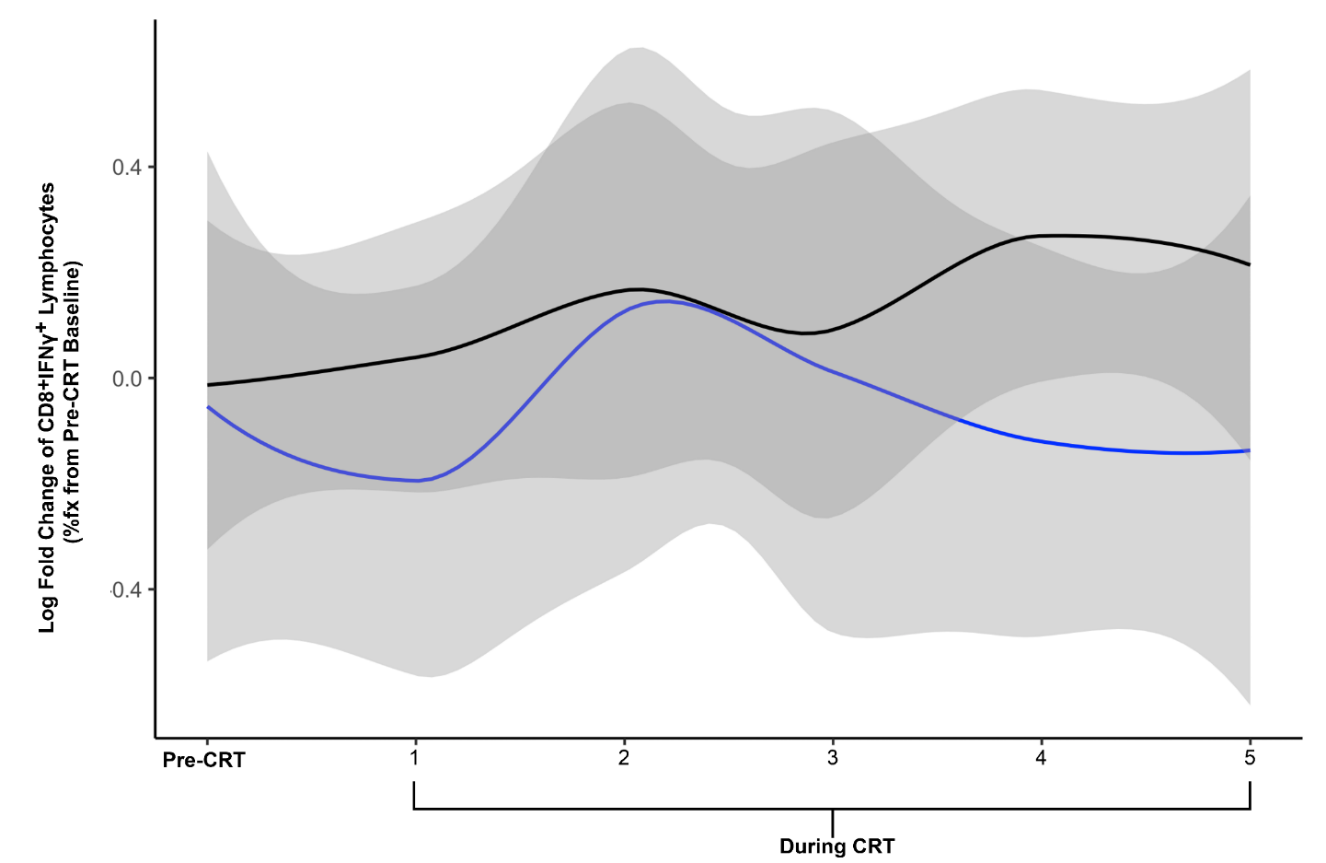


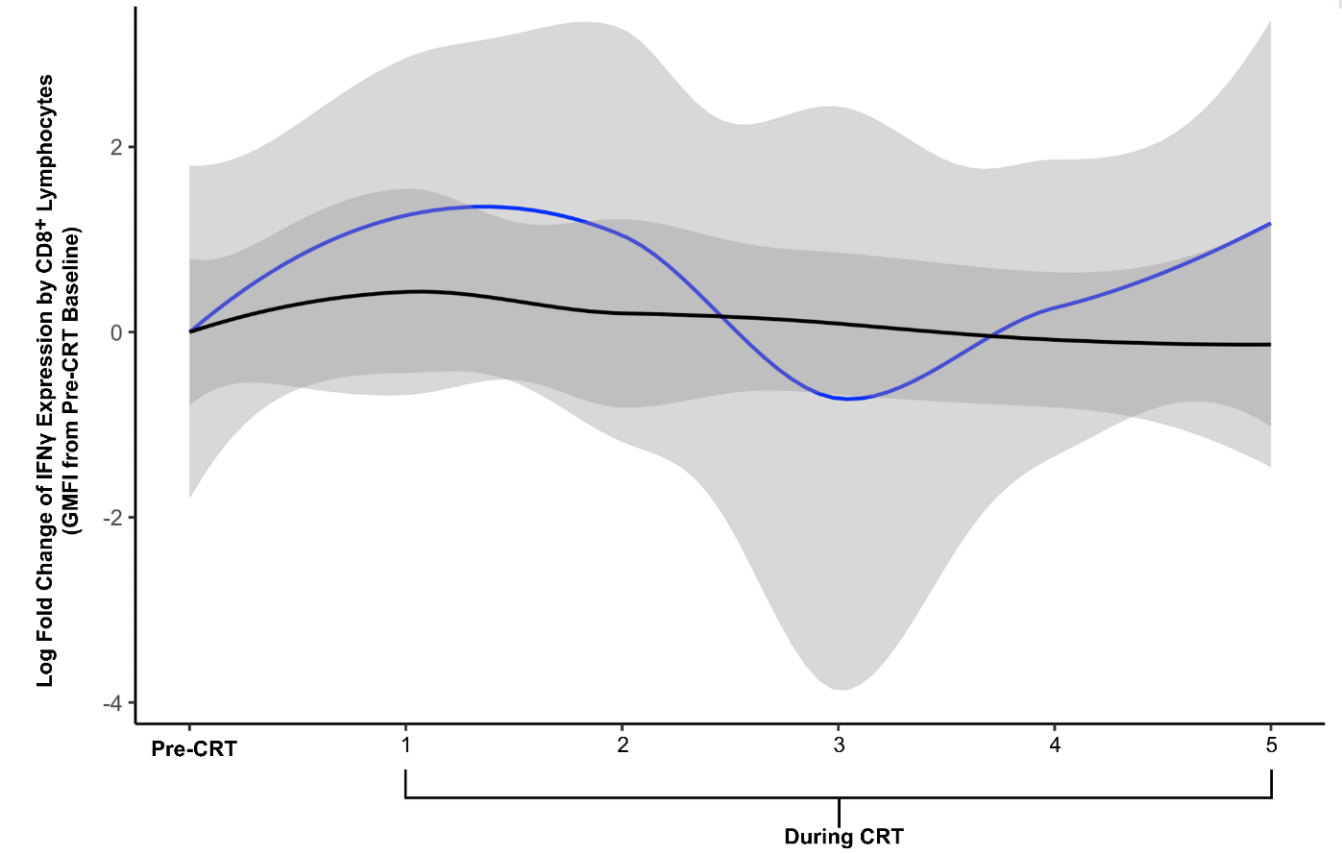


**Supplementary Figure 7a.** IFNγ Expression by Flow Cytometry by CD8^+^ T Lymphocytes: %fx (top) and GMFI (bottom).


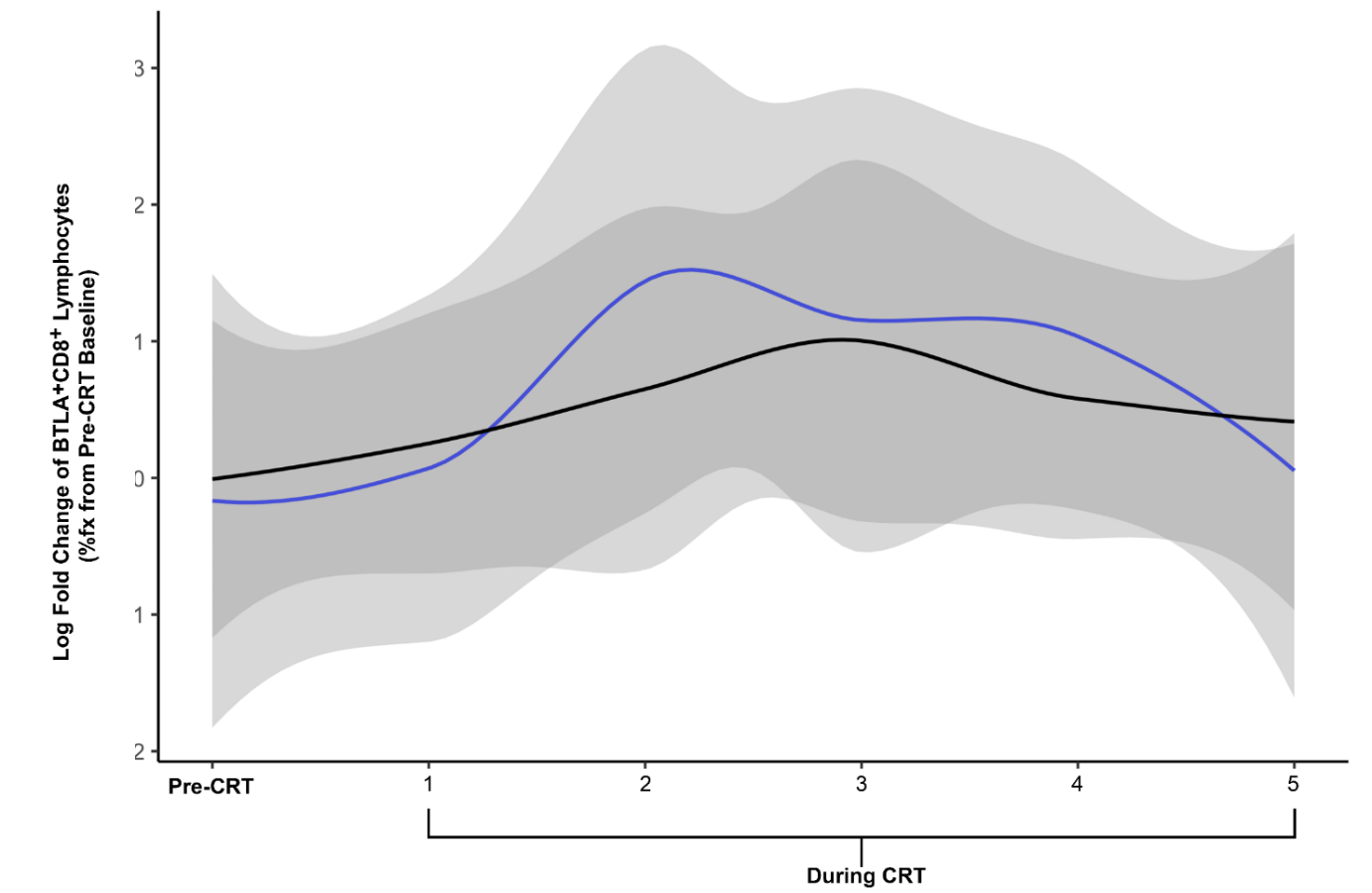


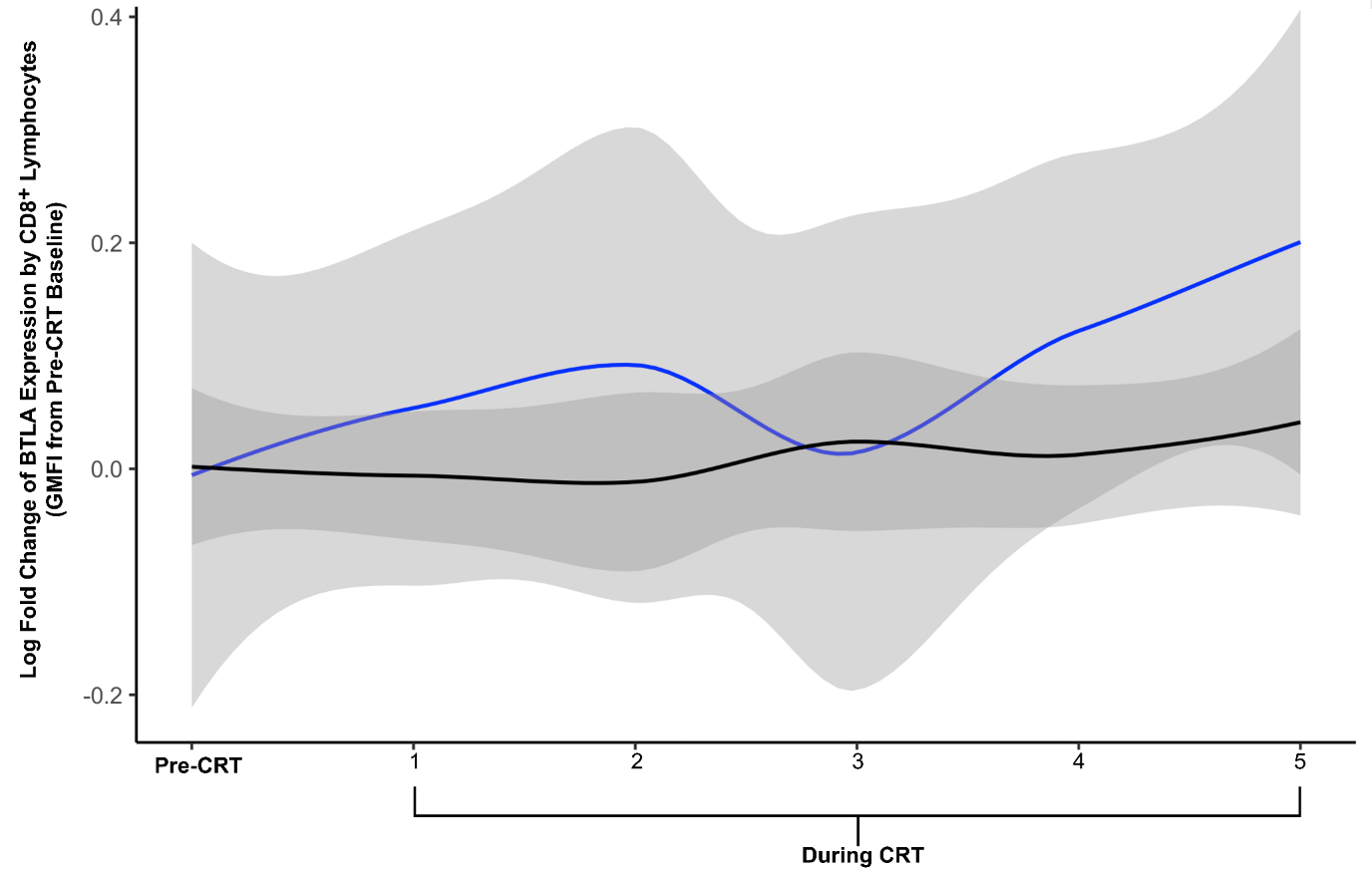


**Supplementary Figure 7b.** BTLA4 Expression by Flow Cytometry by CD8^+^ T Lymphocytes: %fx (top) and GMFI (bottom).


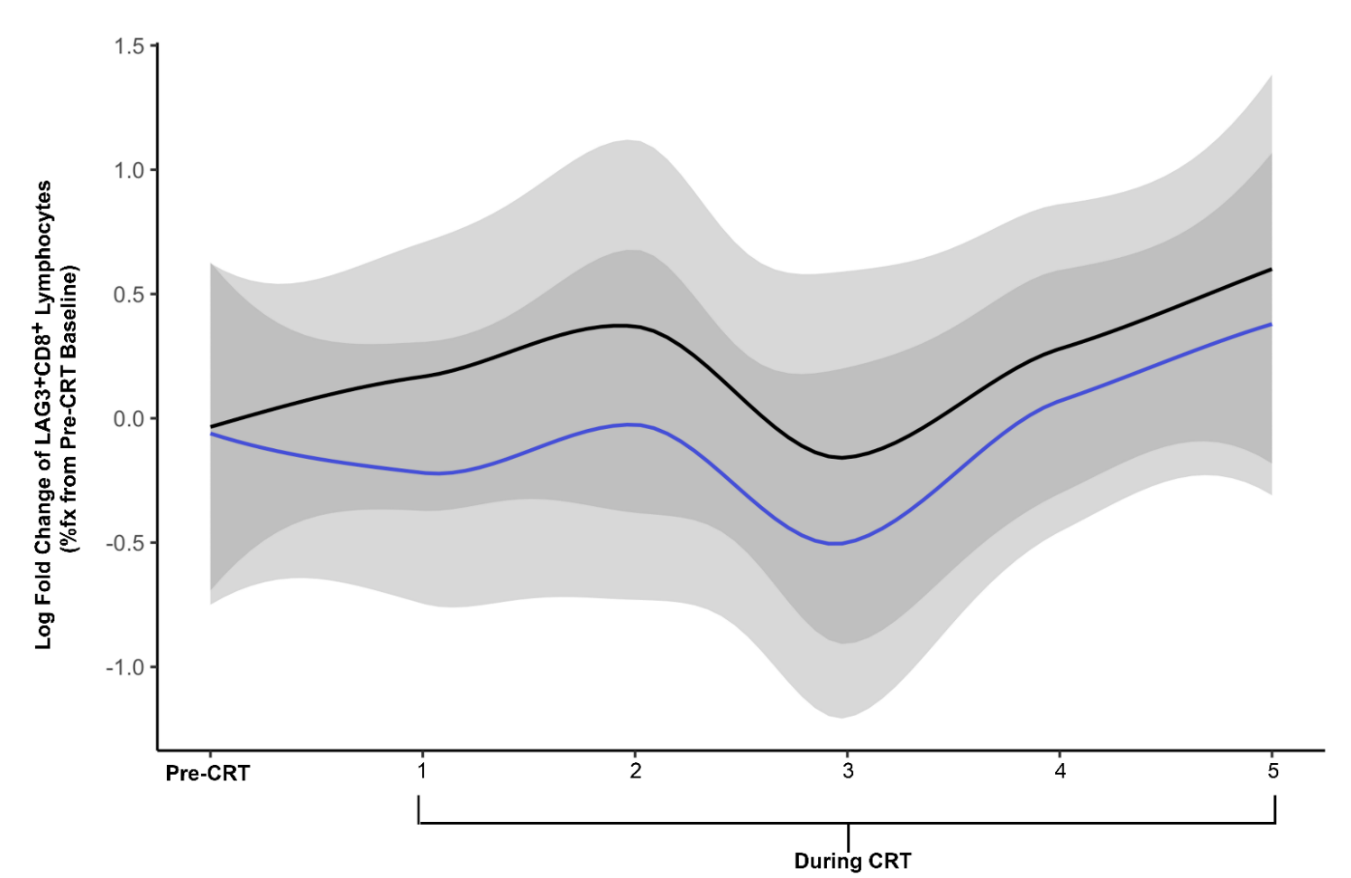


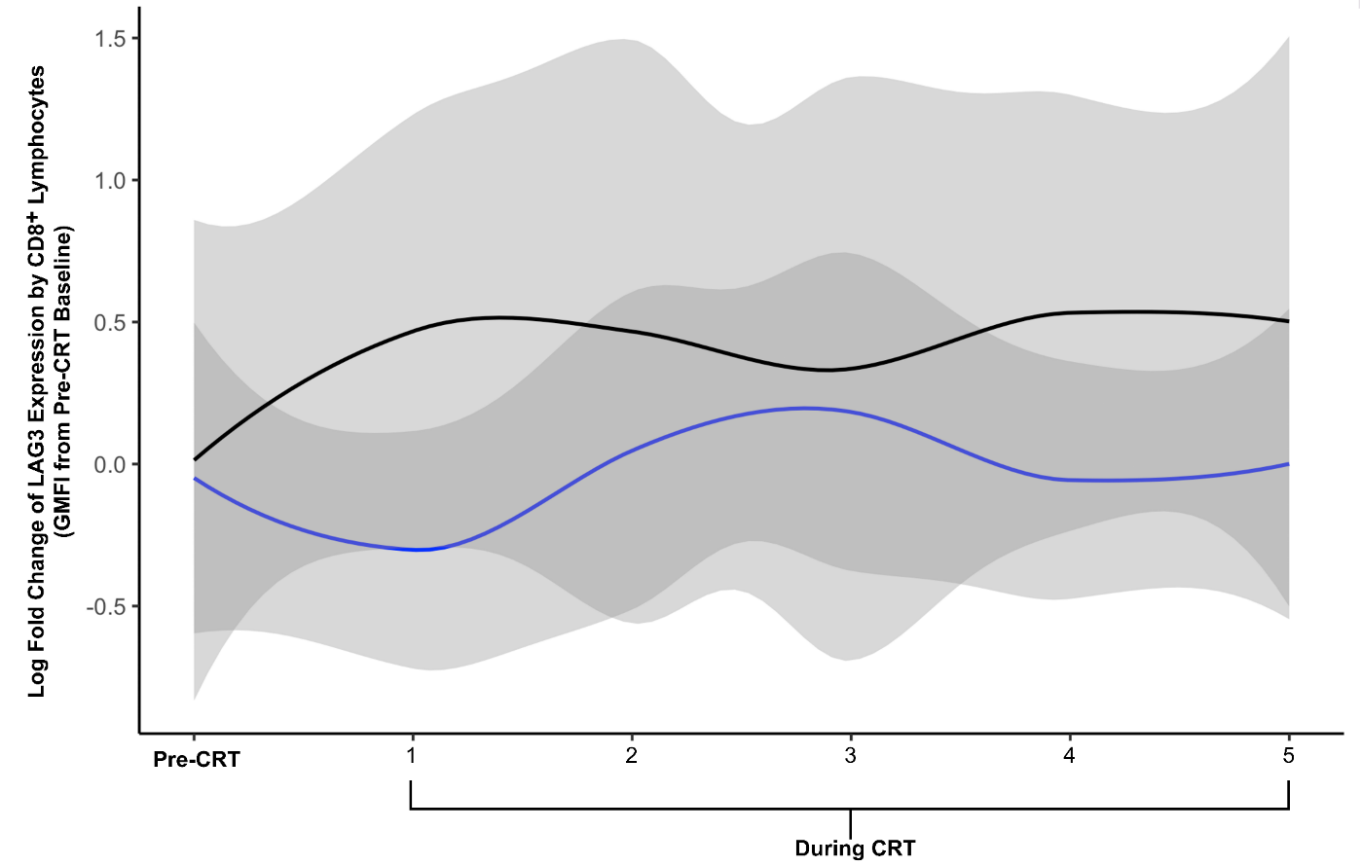


**Supplementary Figure 7c.** LAG3 Expression by Flow Cytometry by CD8^+^ T Lymphocytes: %fx (top) and GMFI (bottom).


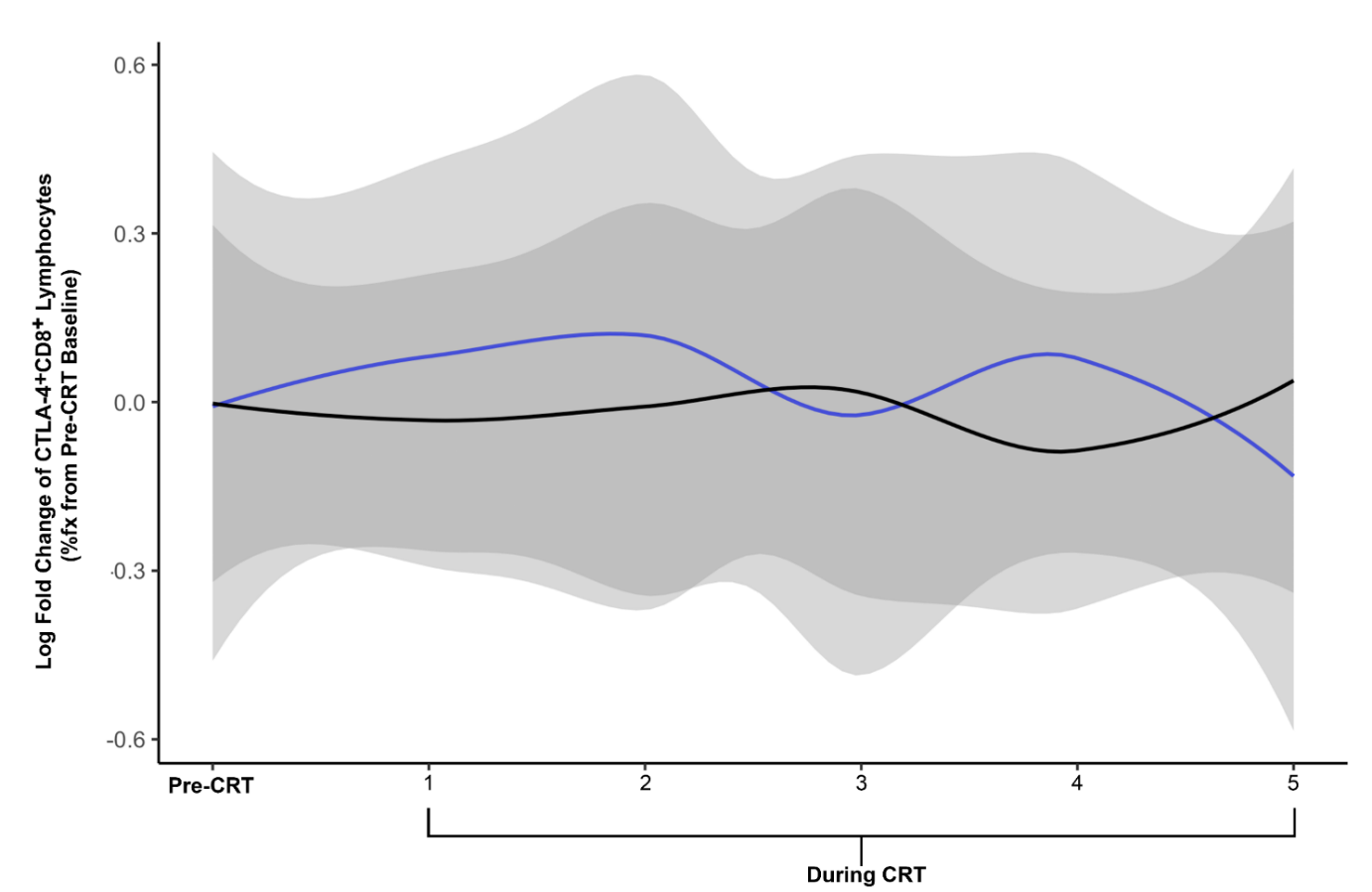


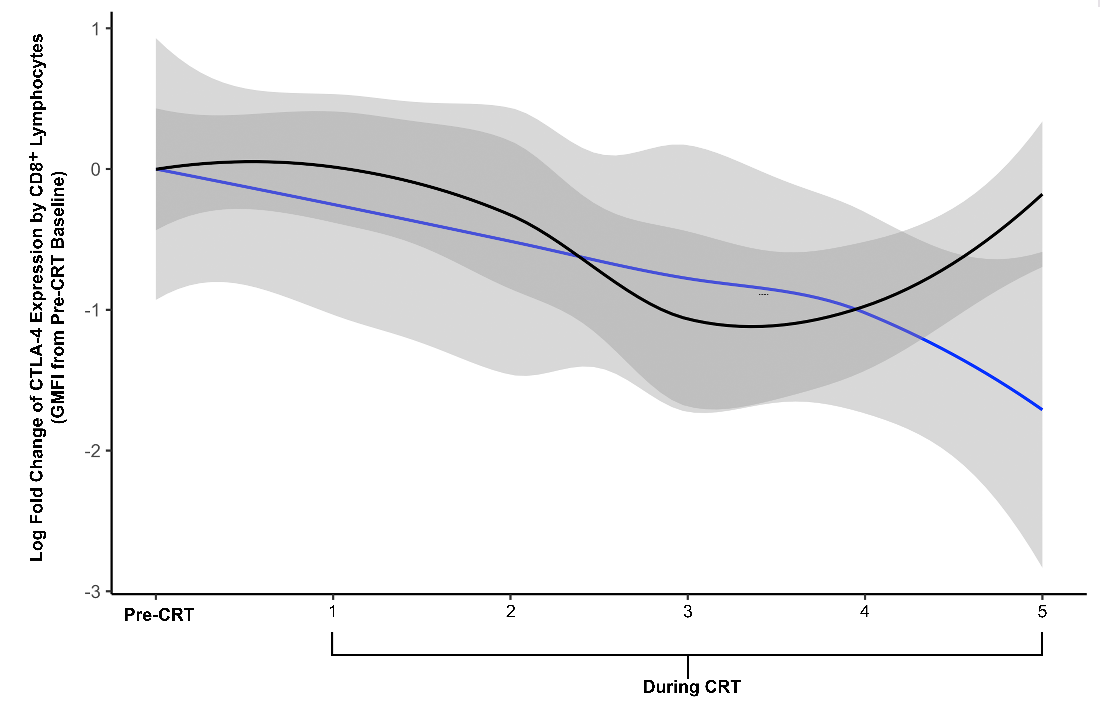


**Supplementary Figure 7d.** CTLA-4 Expression by Flow Cytometry by CD8^+^ T Lymphocytes: %fx (top) and GMFI (bottom).


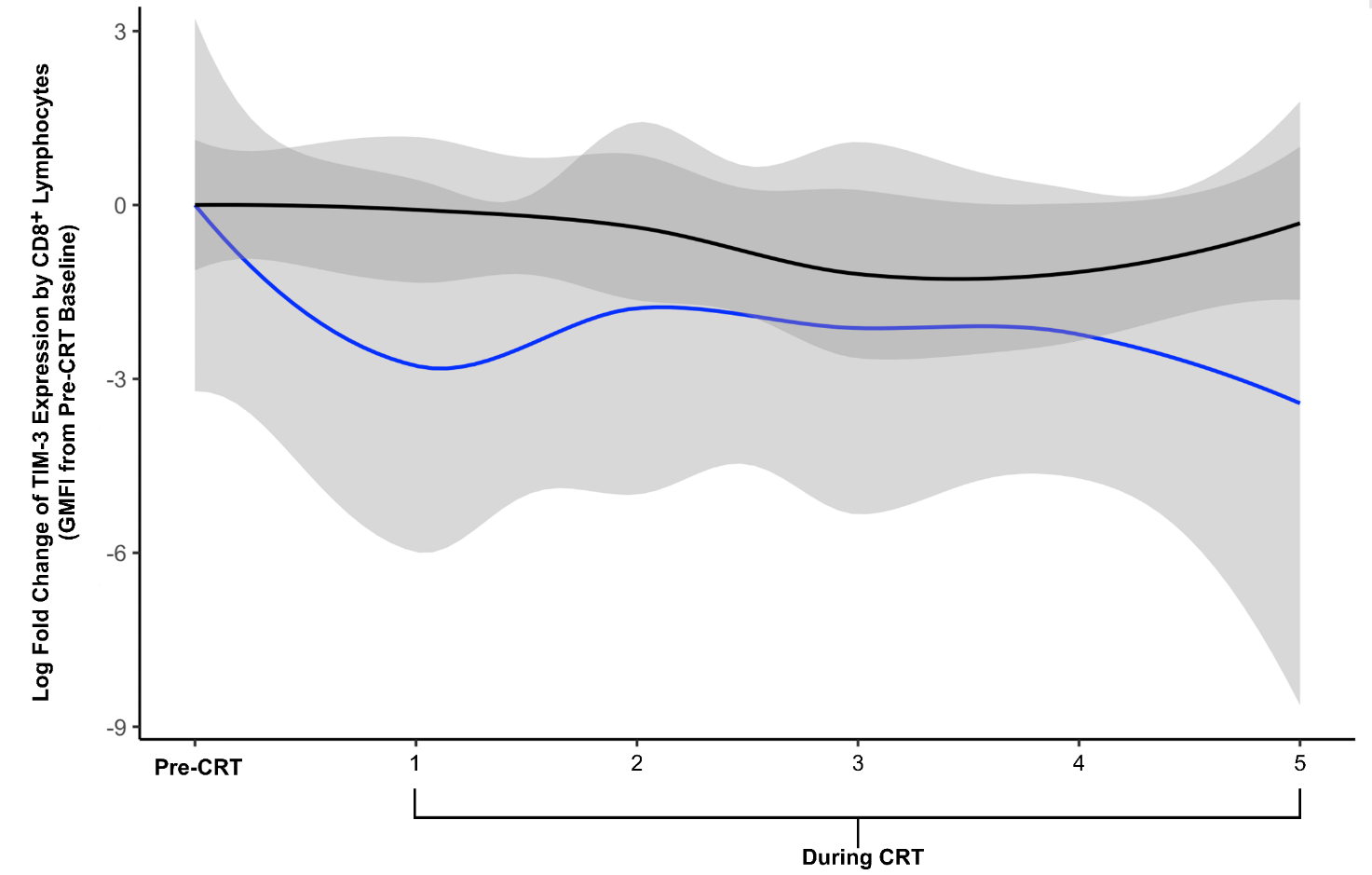


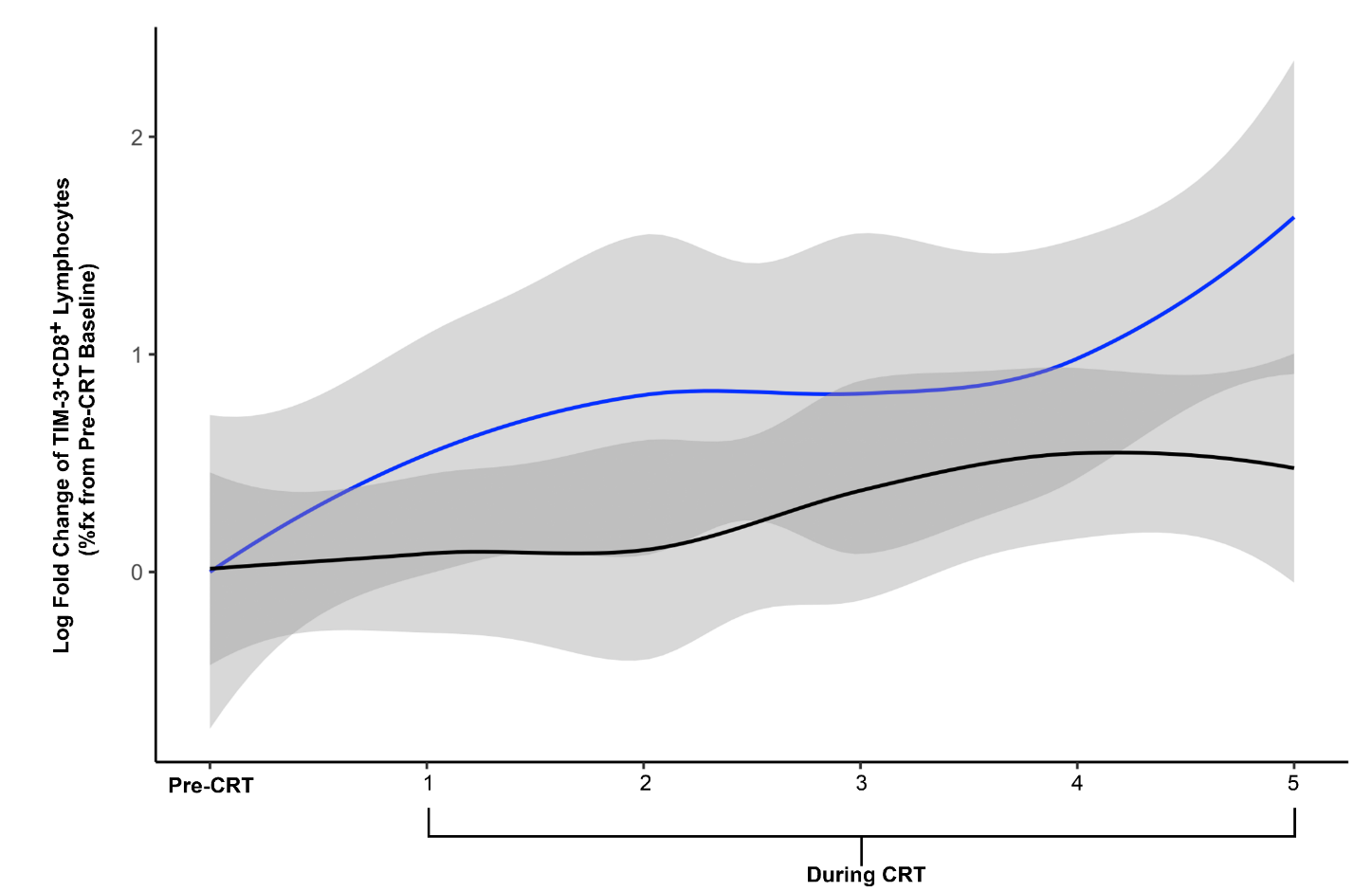


**Supplementary Figure 7e.** TIM-3 Expression by Flow Cytometry by CD8^+^ T Lymphocytes: %fx (top) and GMFI (bottom).


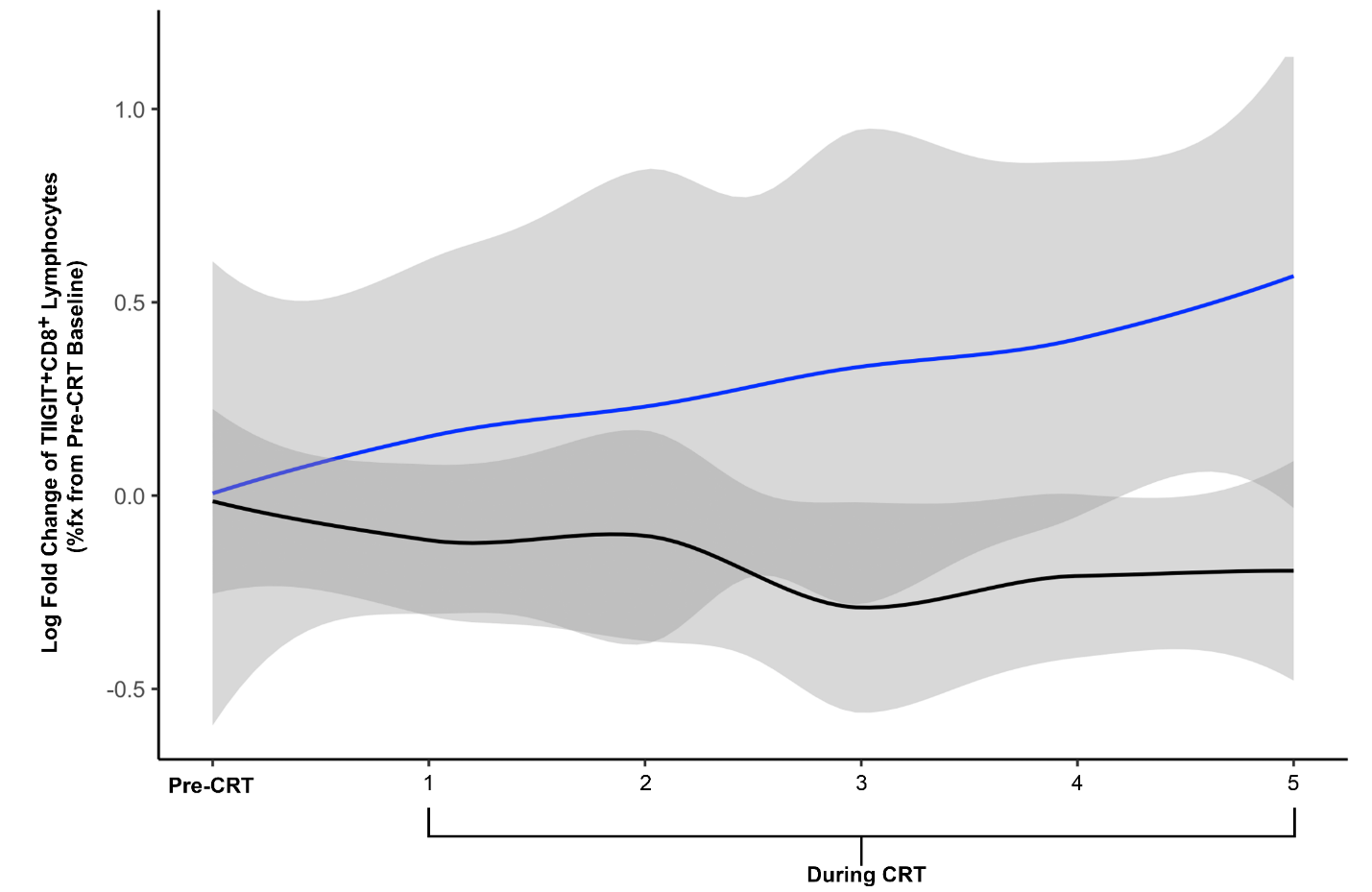


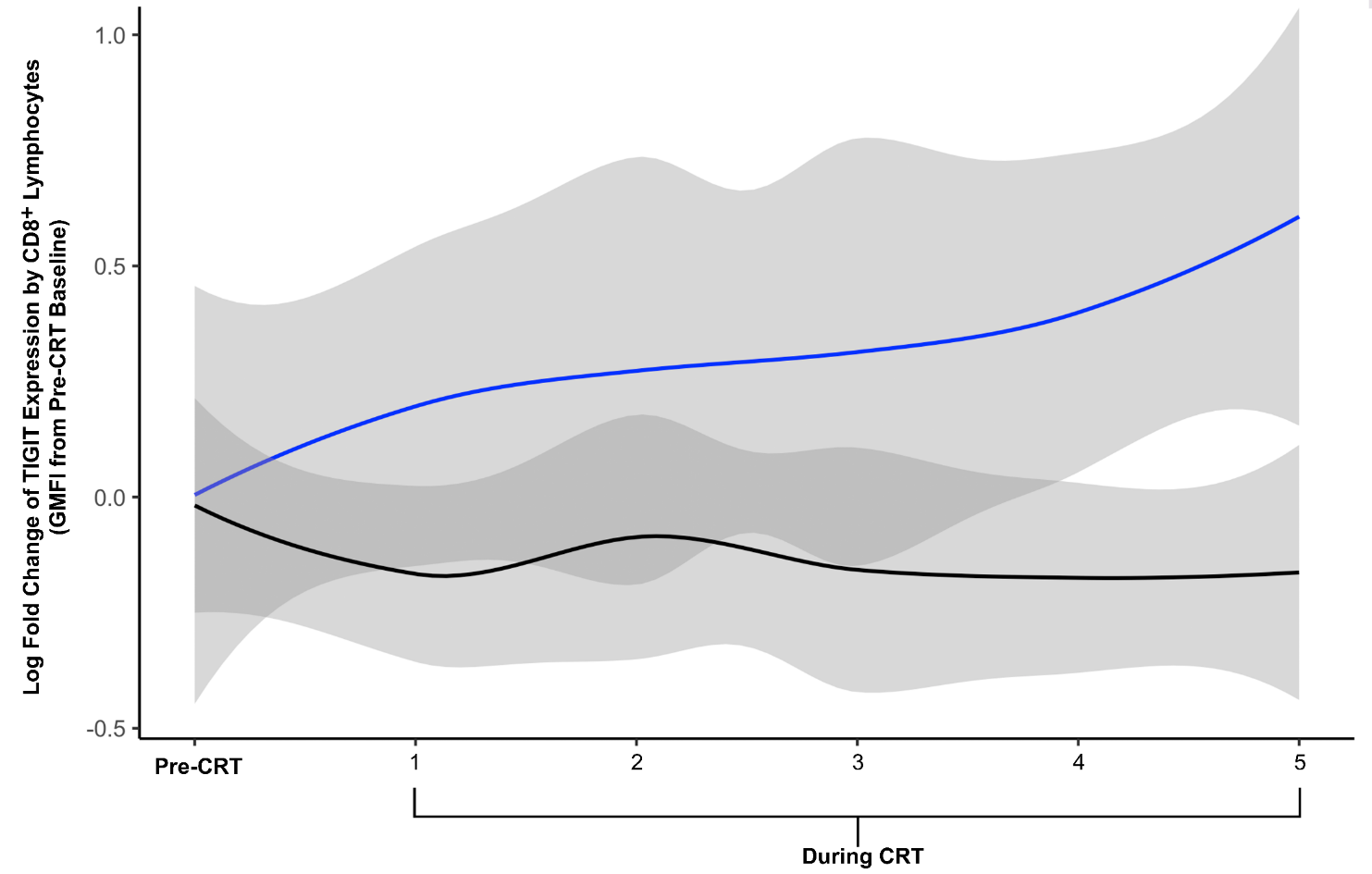


**Supplementary Figure 7f.** TIGIT Expression by Flow Cytometry by CD8^+^ T Lymphocytes: %fx (top) and GMFI (bottom).


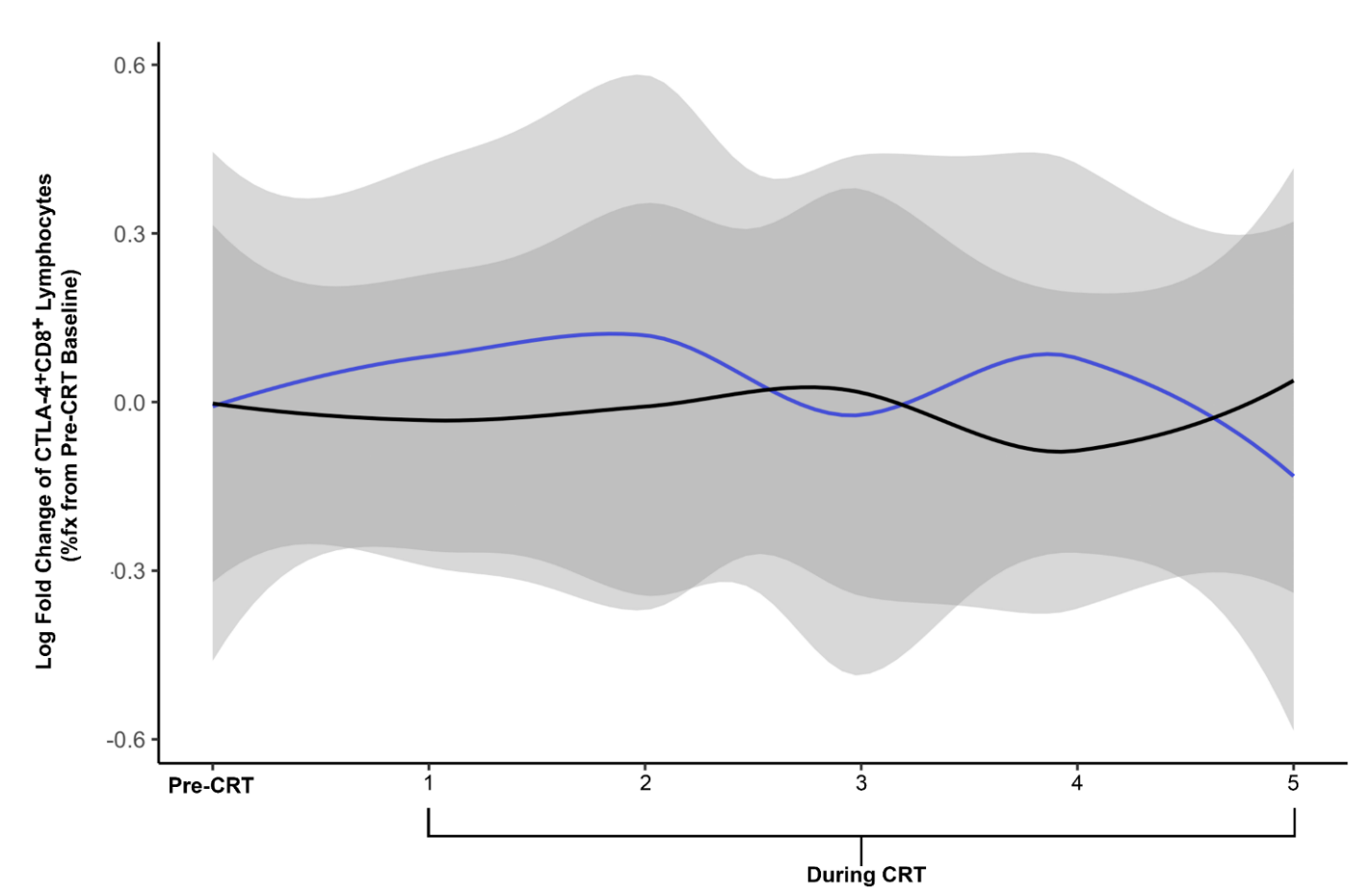


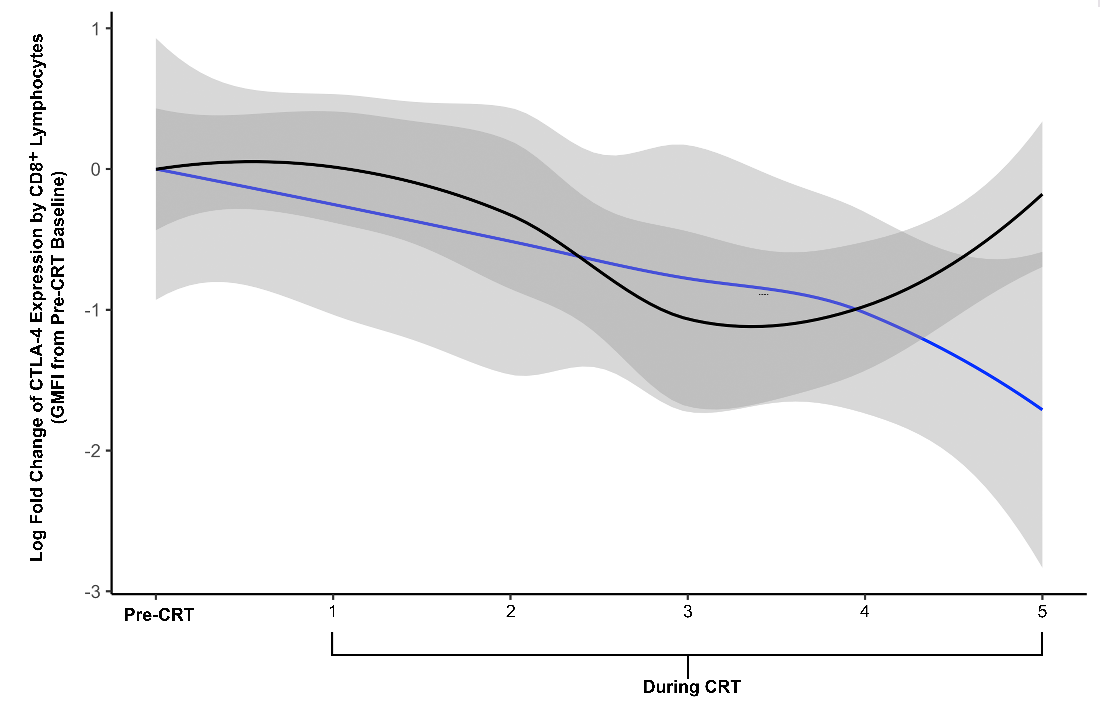


**Supplementary Figure 7g.** CTLA-4 Expression by Flow Cytometry by CD8^+^ T Lymphocytes: %fx (top) and GMFI (bottom).


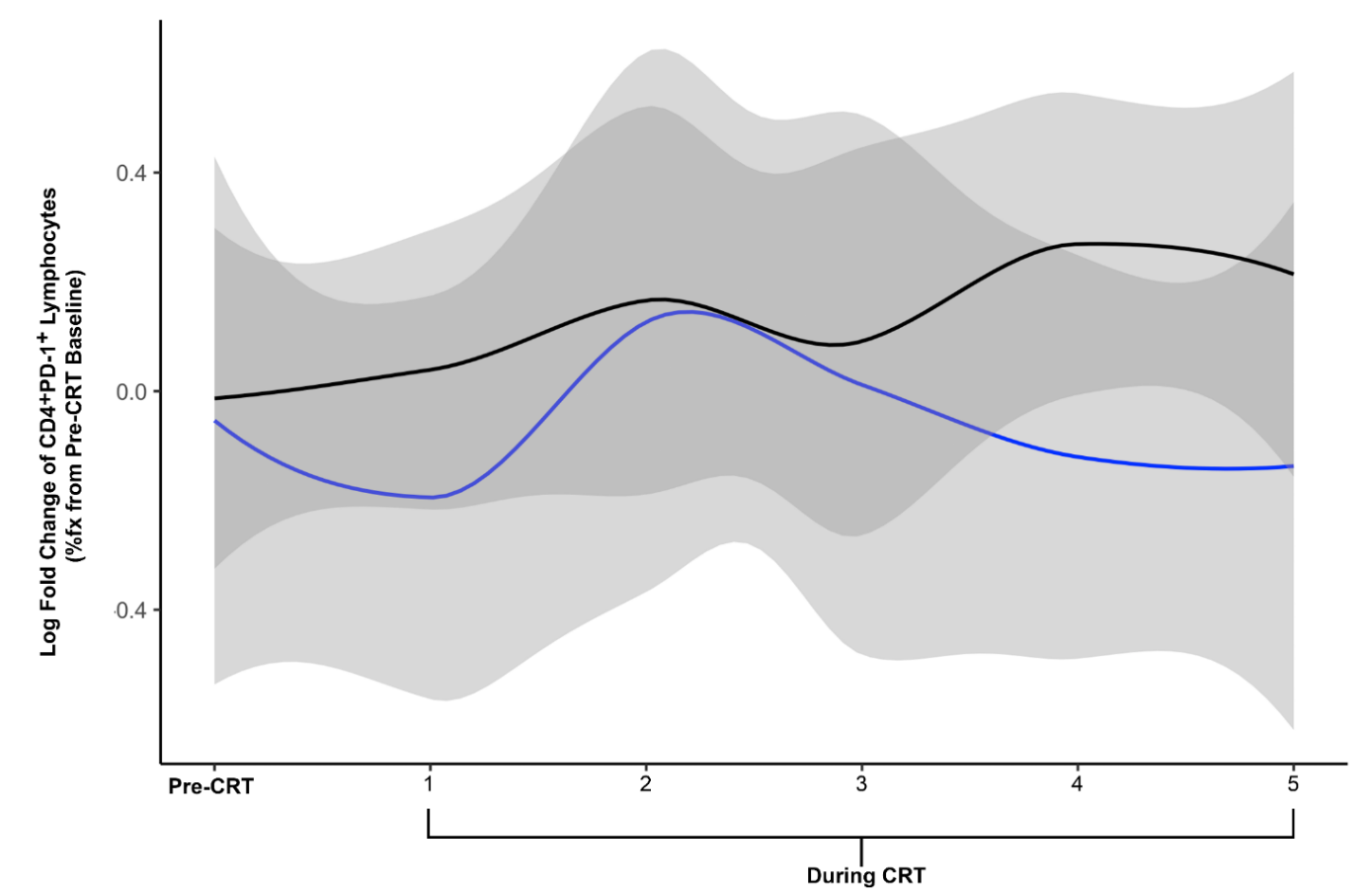


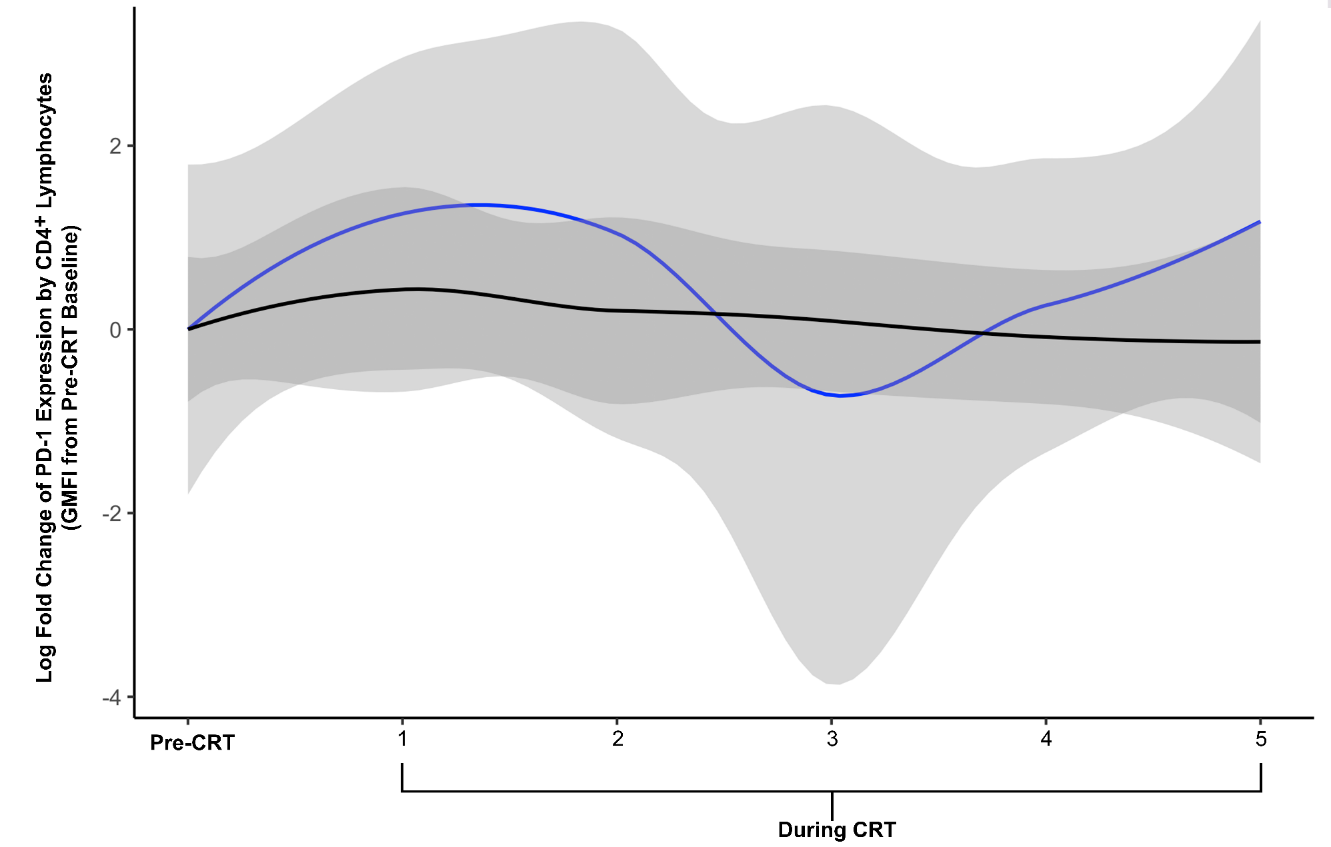


**Supplementary Figure 8a.** PD-1 Expression by Flow Cytometry by CD4^+^ T Lymphocytes: %fx (top) and GMFI (bottom).


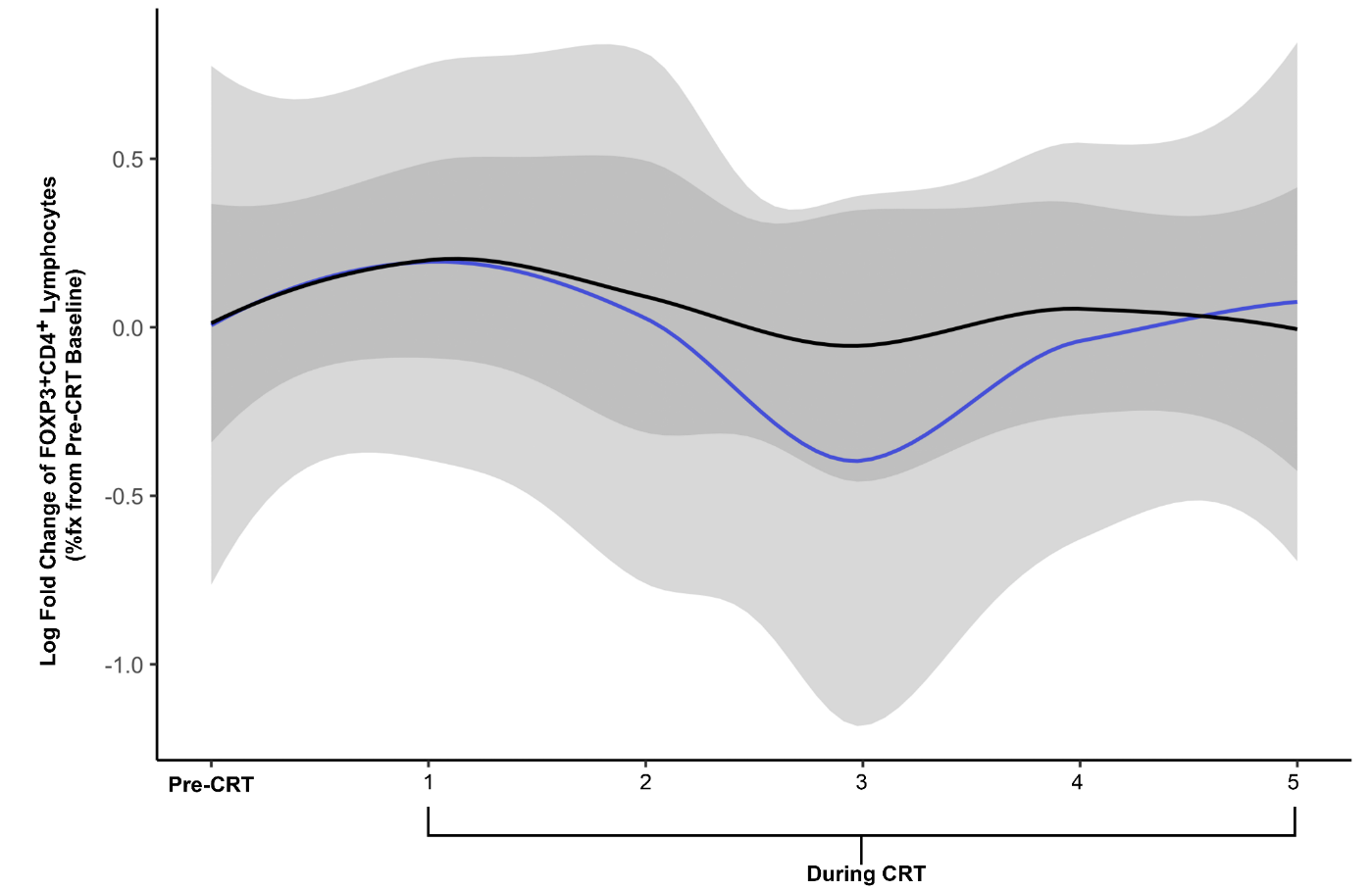


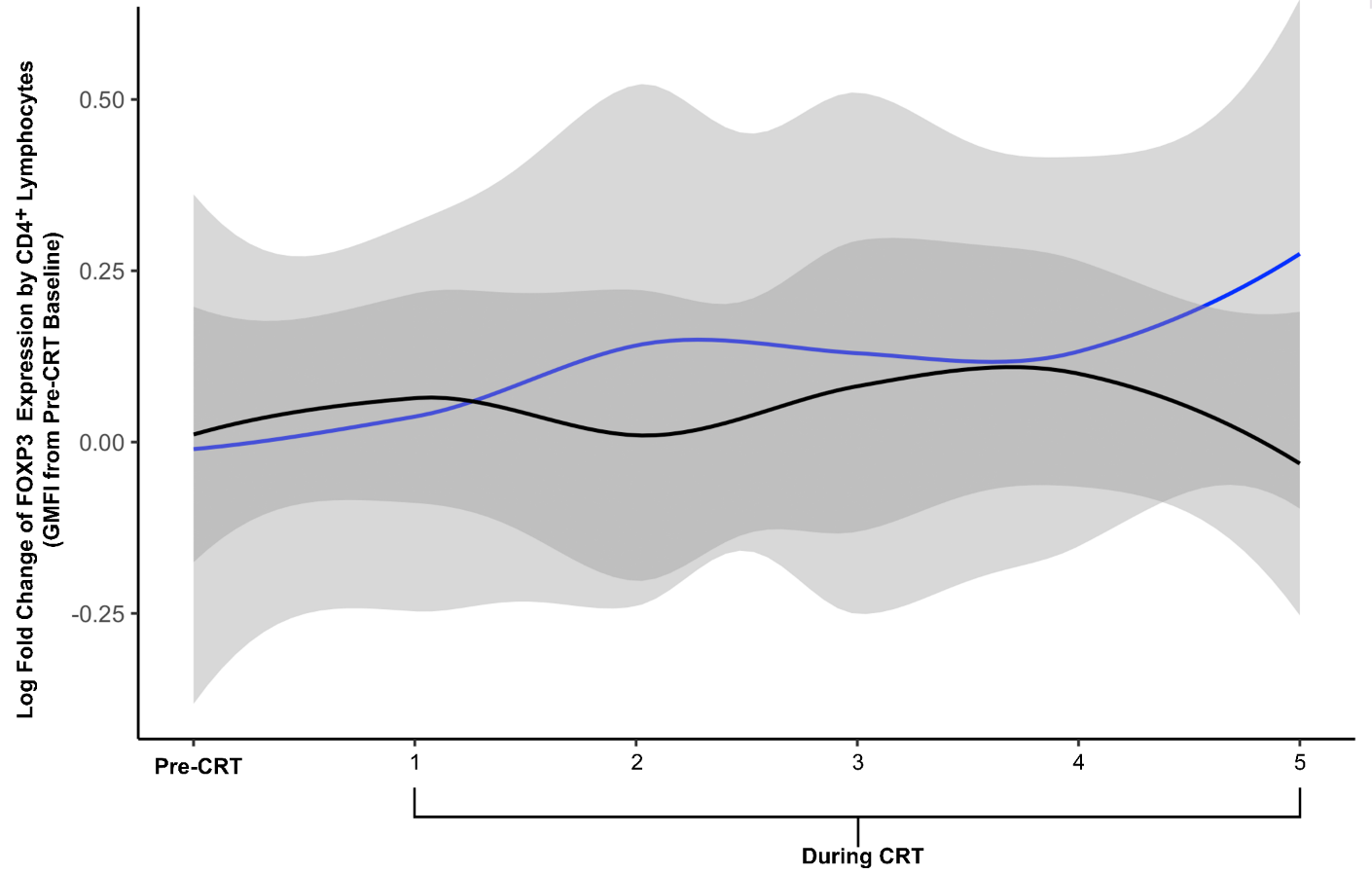


**Supplementary Figure 8b.** FOXP3 Expression by Flow Cytometry by CD4^+^ T Lymphocytes: %fx (top) and GMFI (bottom).


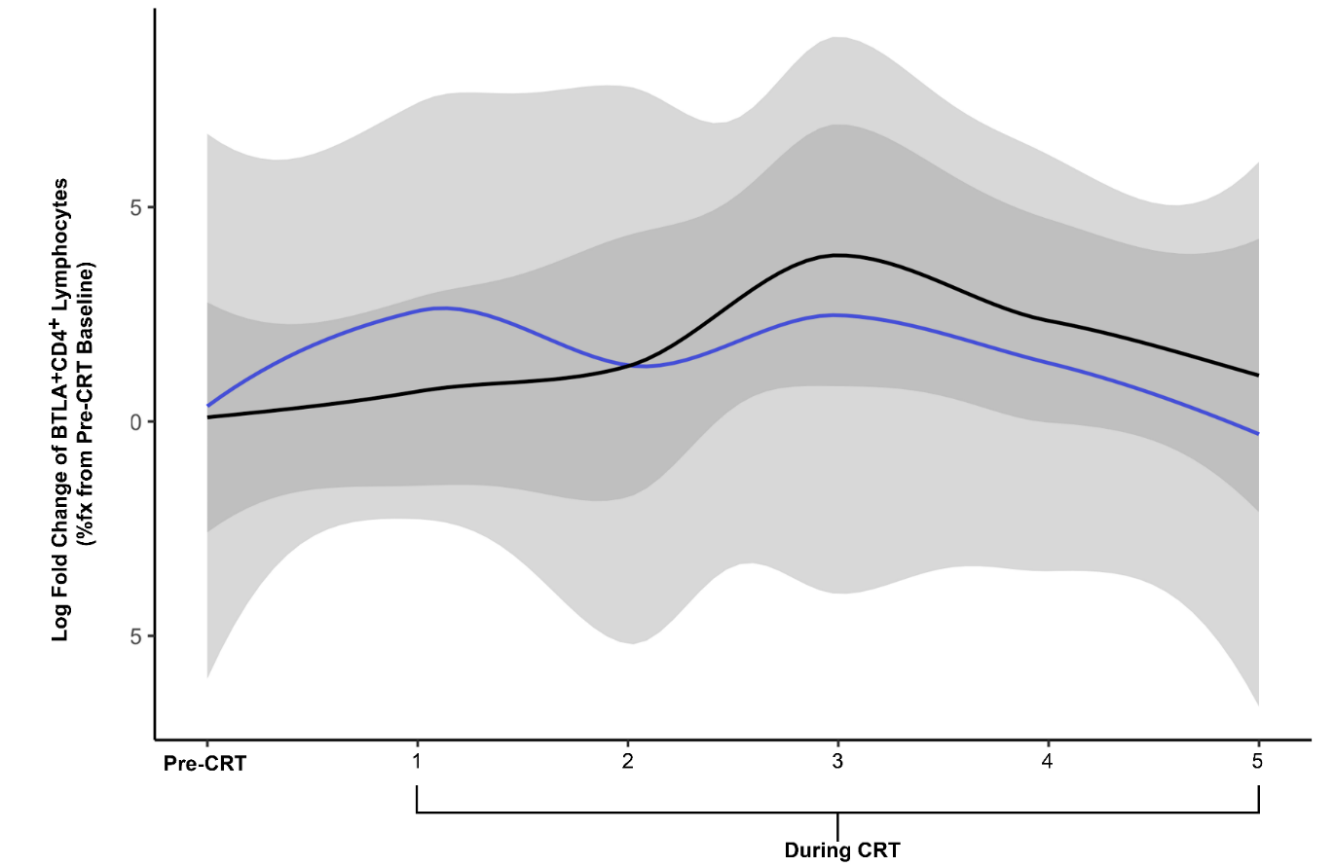


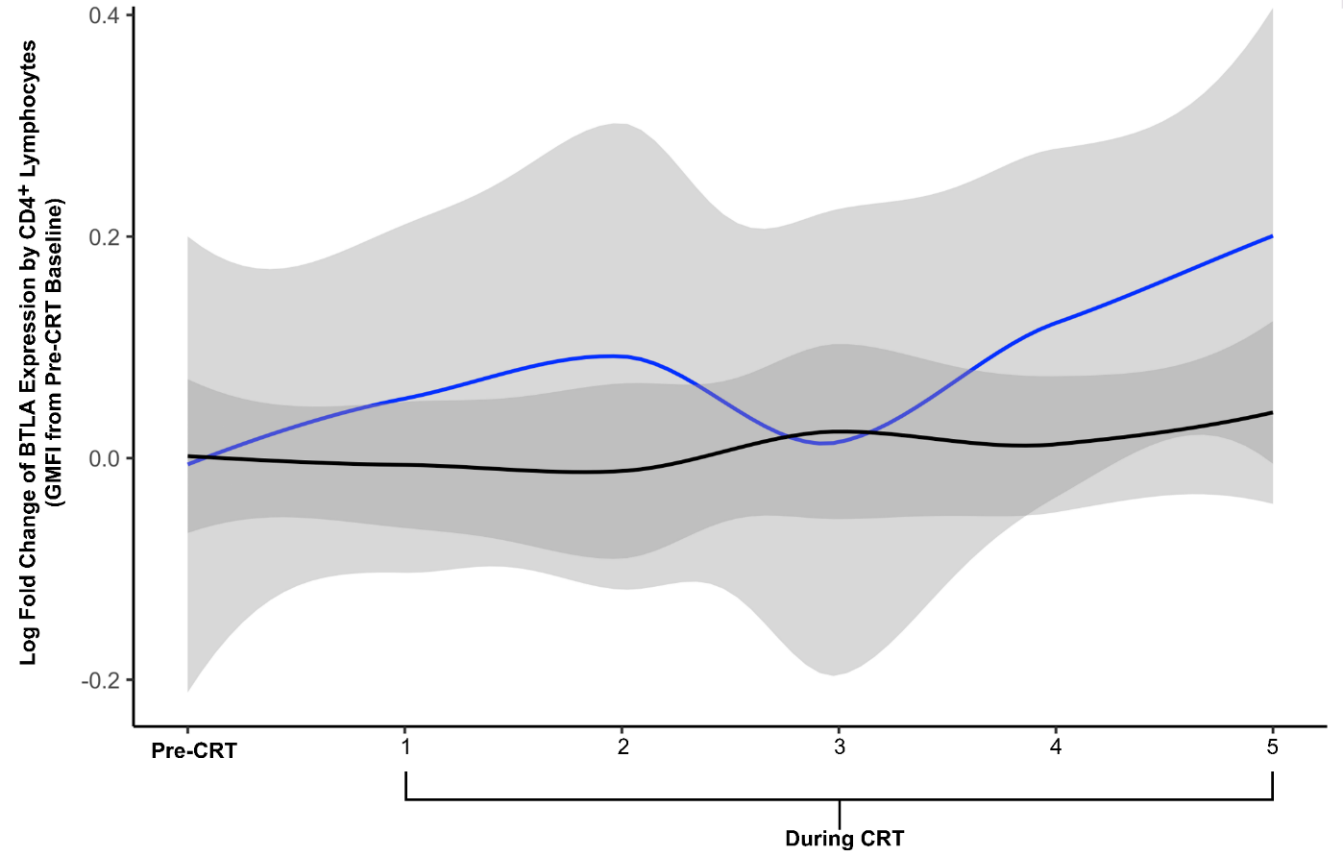


**Supplementary Figure 8c.** BTLA Expression by Flow Cytometry by CD4^+^ T Lymphocytes: %fx (top) and GMFI (bottom).


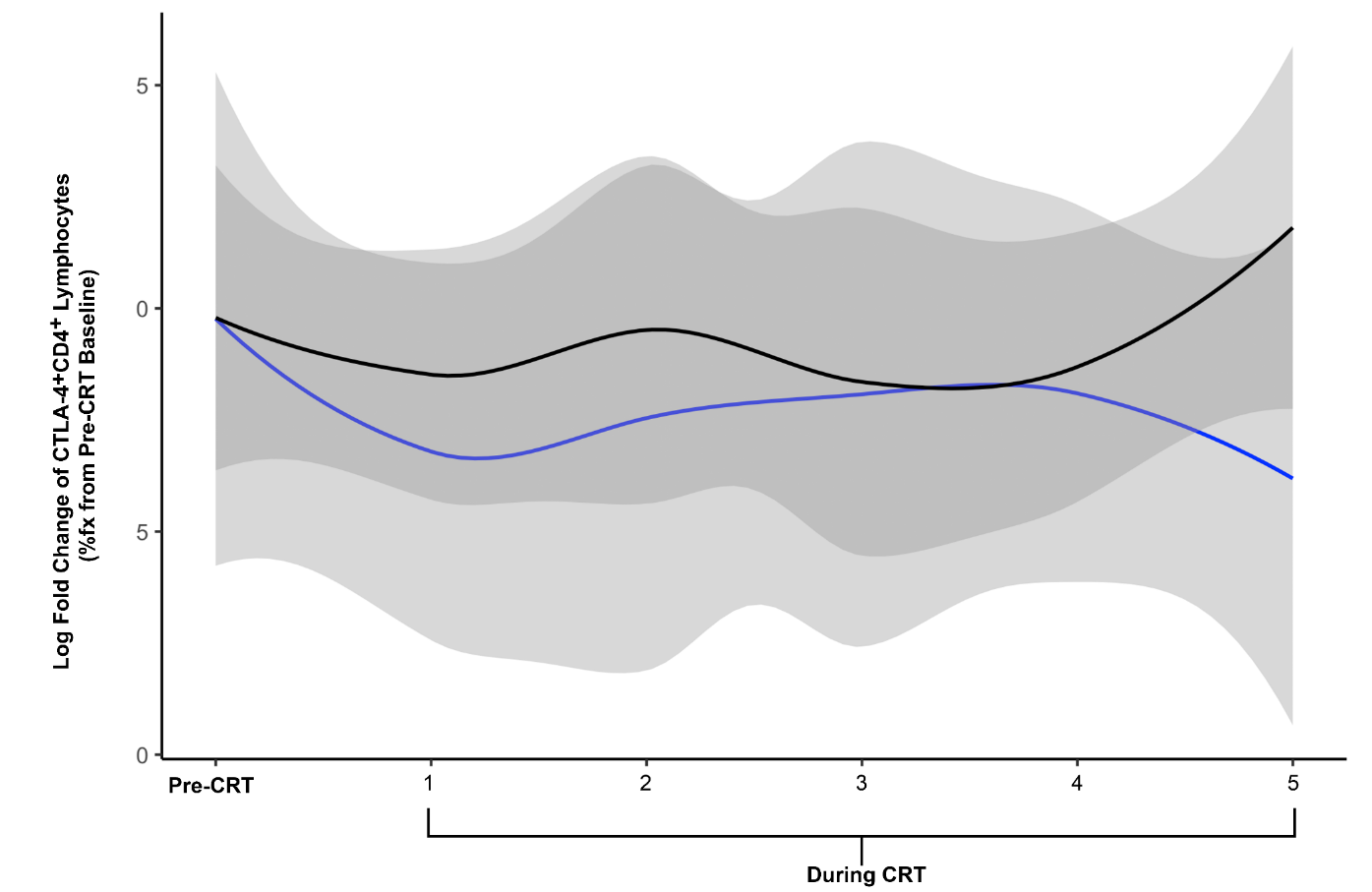


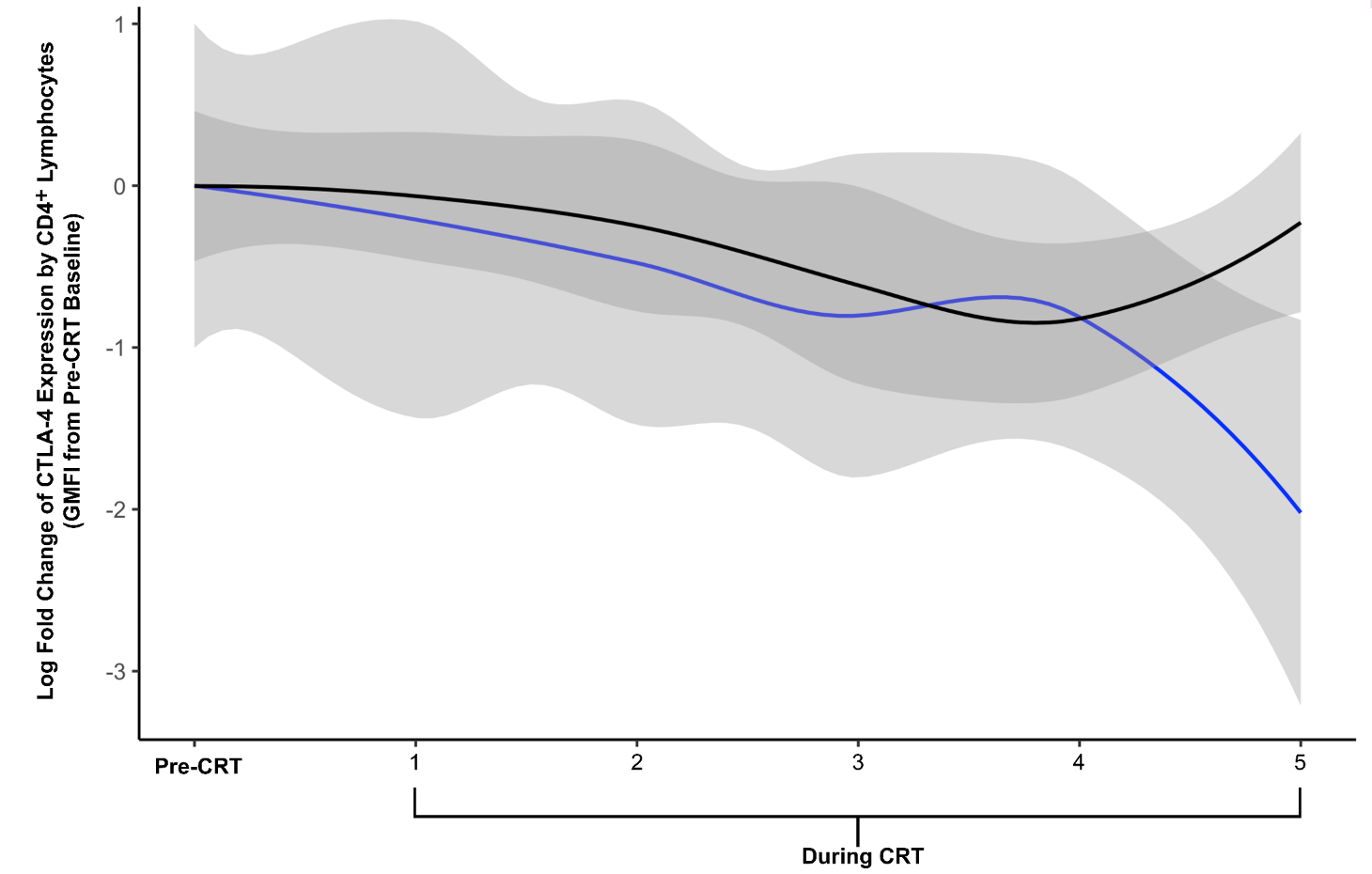


**Supplementary Figure 8d.** CTLA-4 Expression by Flow Cytometry by CD4^+^ T Lymphocytes: %fx (top) and GMFI (bottom).


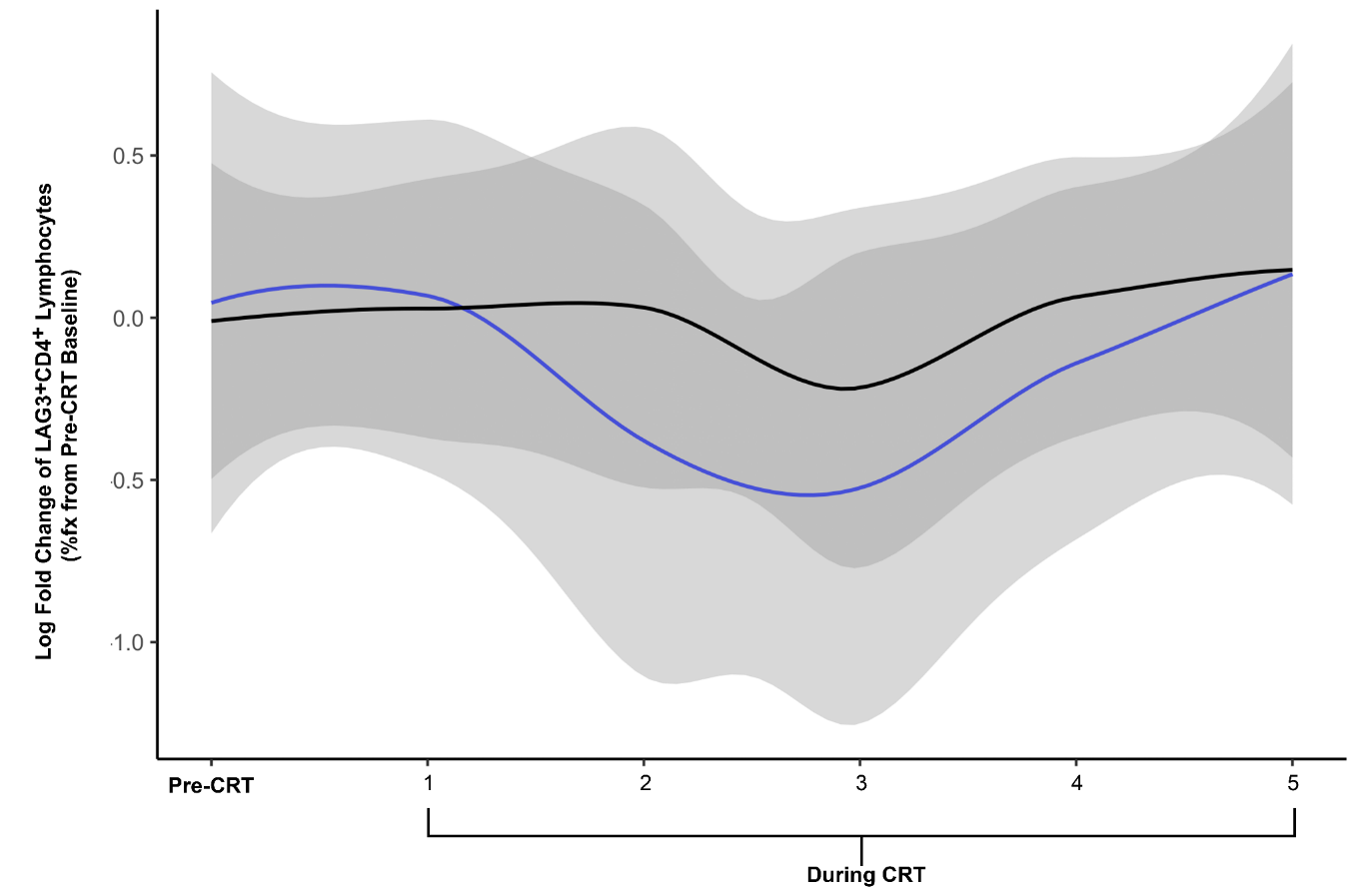


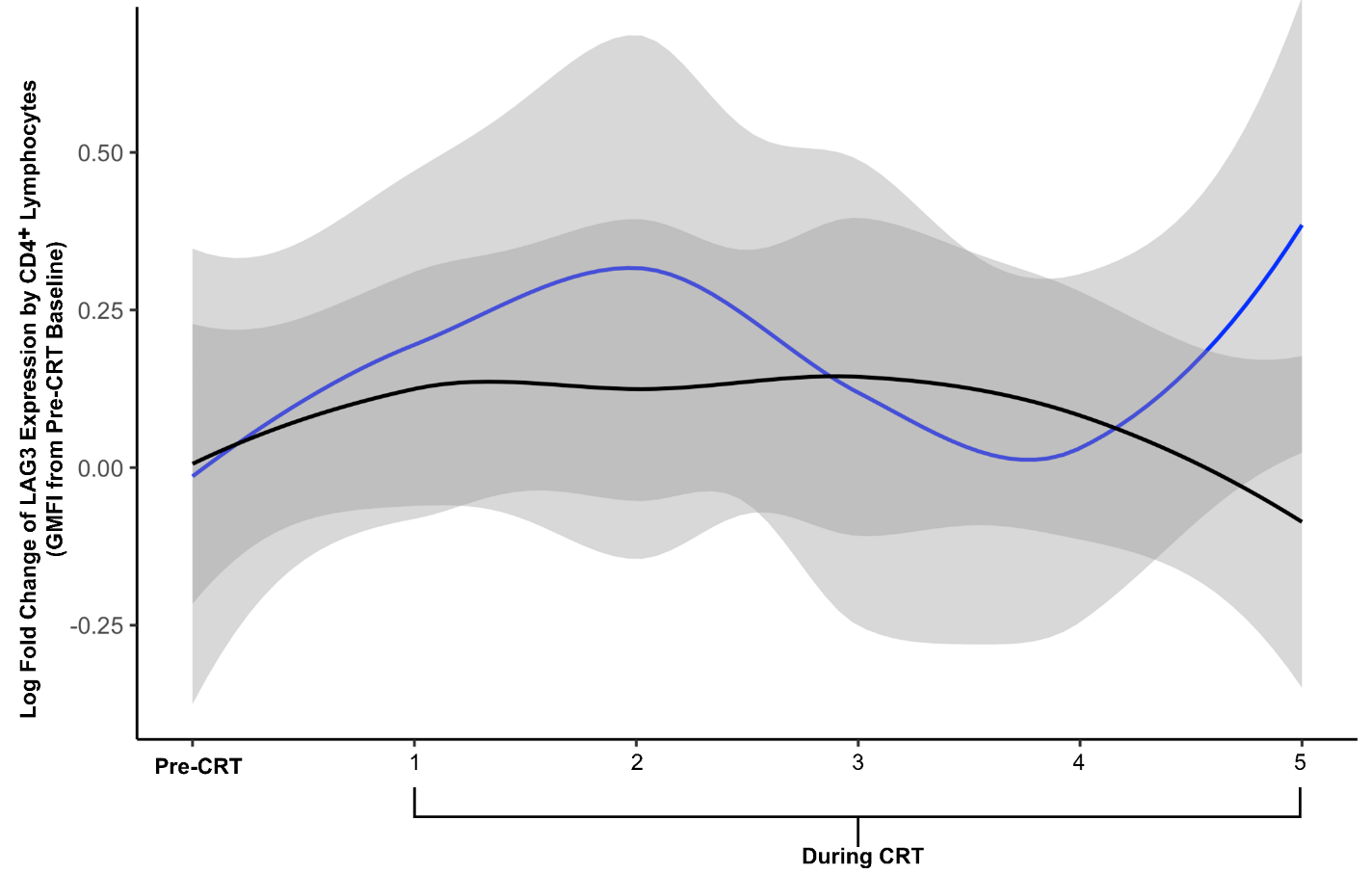


**Supplementary Figure 8e.** LAG3 Expression by Flow Cytometry by CD4^+^ T Lymphocytes: %fx (top) and GMFI (bottom).


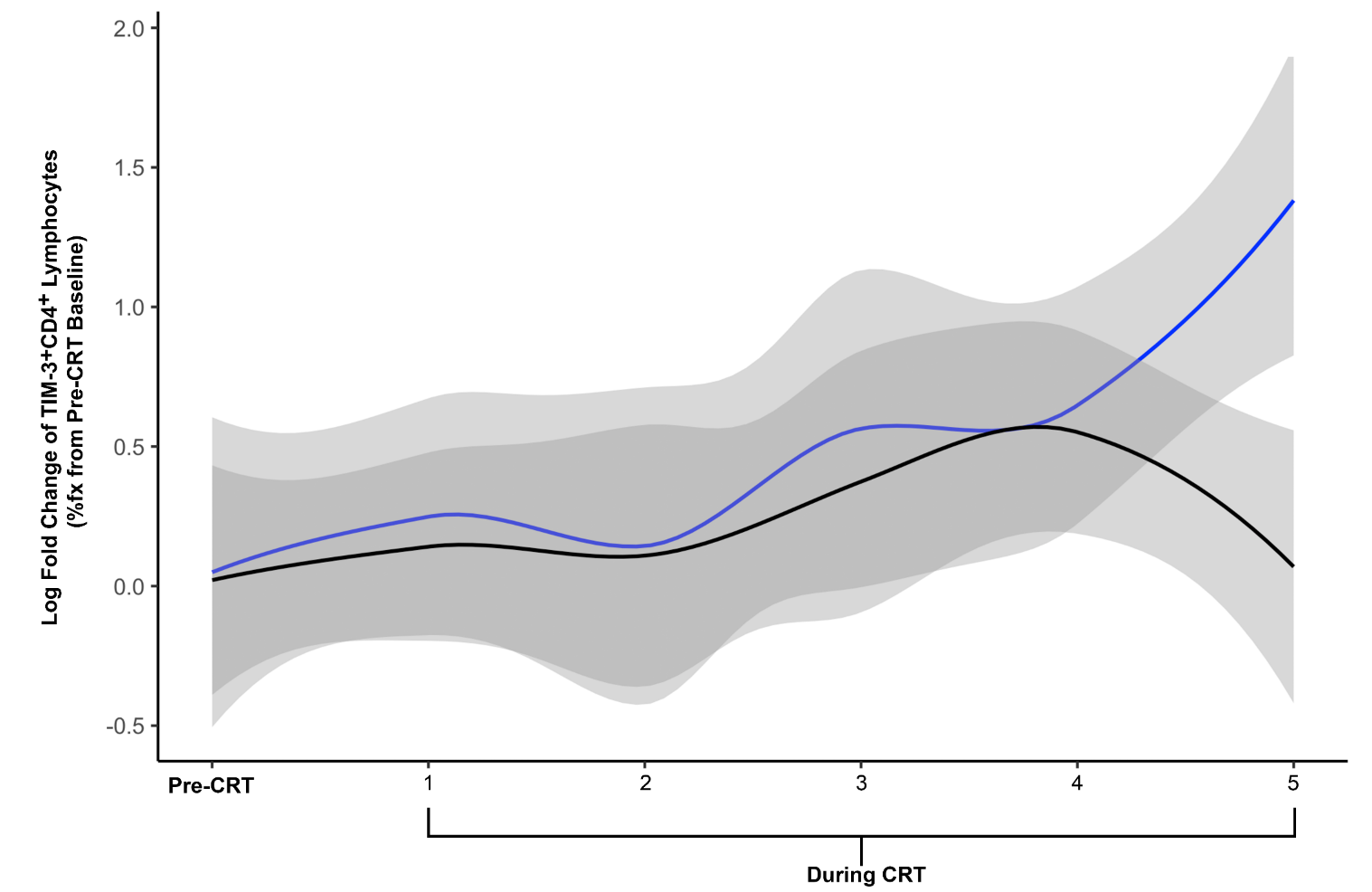


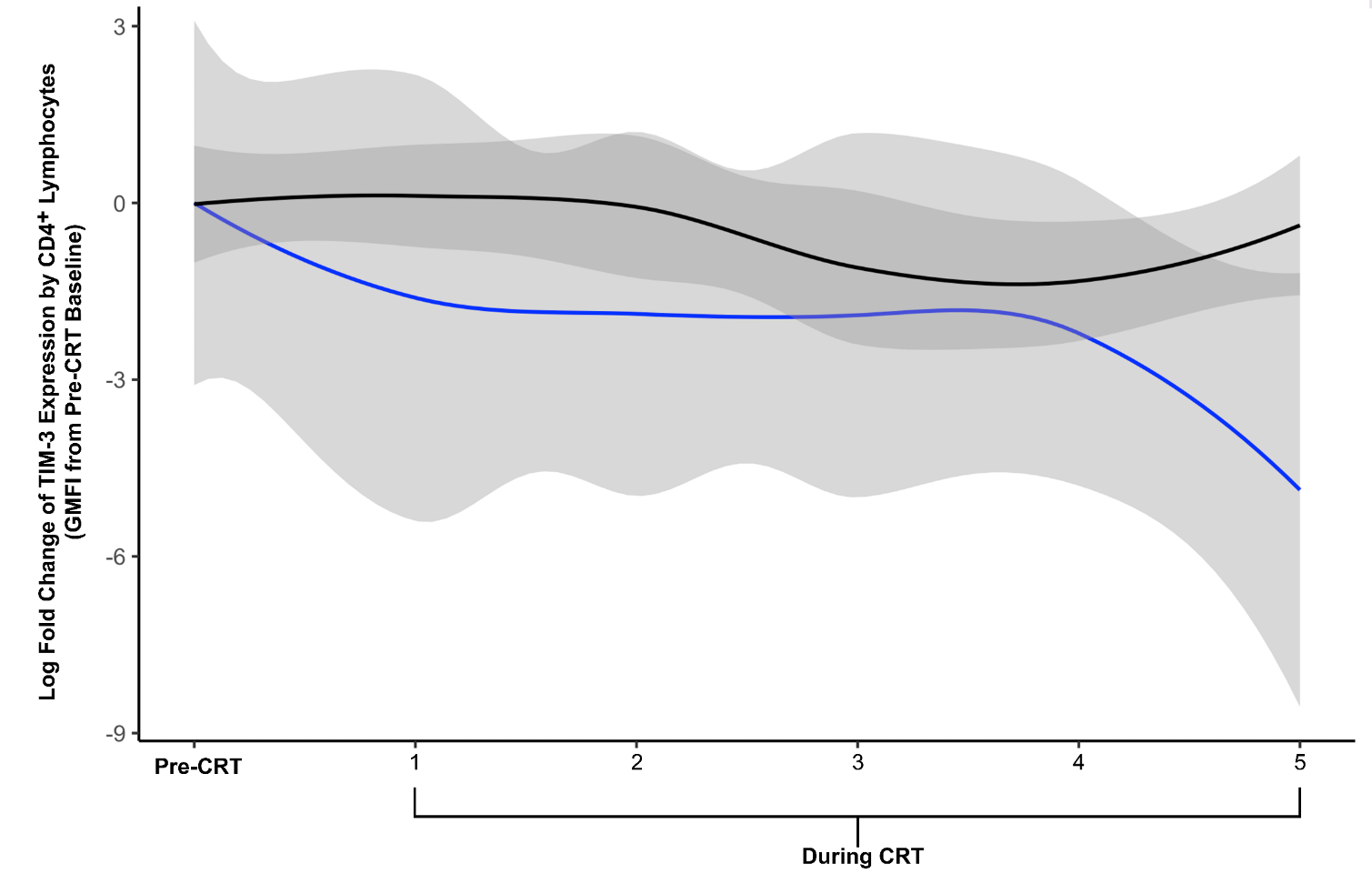


**Supplementary Figure 8f.** TIM-3 Expression by Flow Cytometry by CD4^+^ T Lymphocytes: %fx (top) and GMFI (bottom).


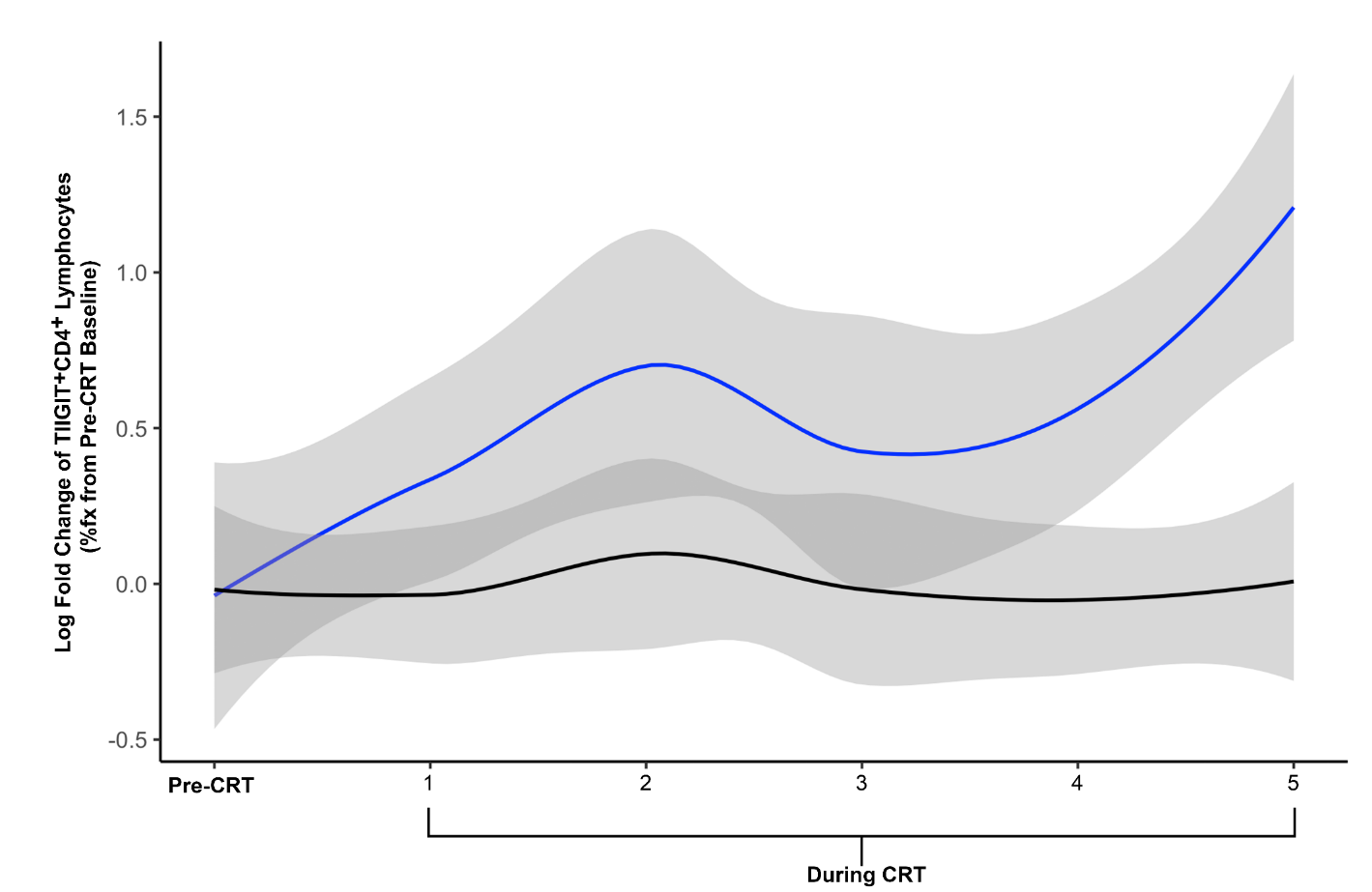


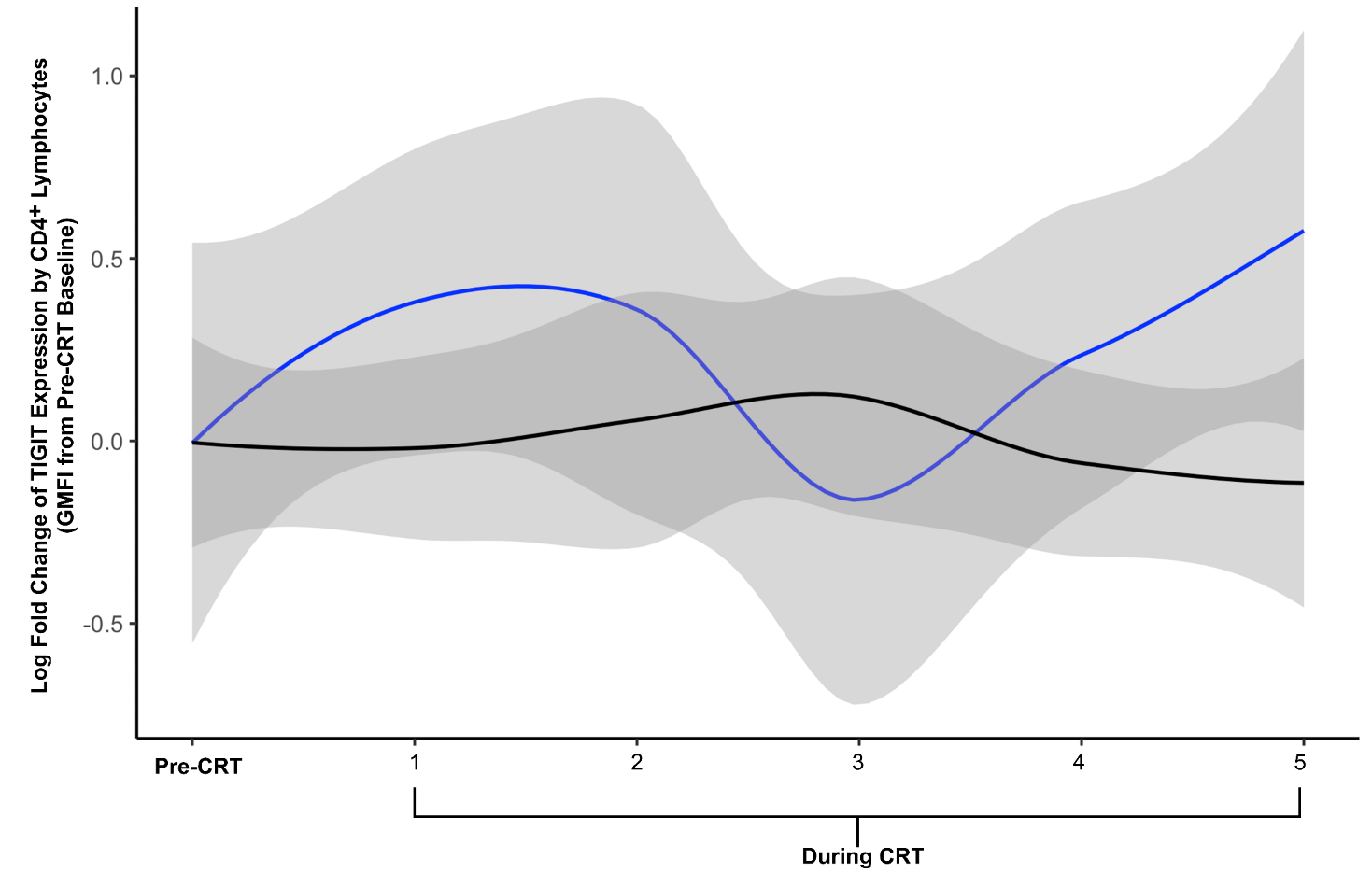


**Supplementary Figure 8g.** TIGIT Expression by Flow Cytometry by CD4^+^ T Lymphocytes: %fx (top) and GMFI (bottom).


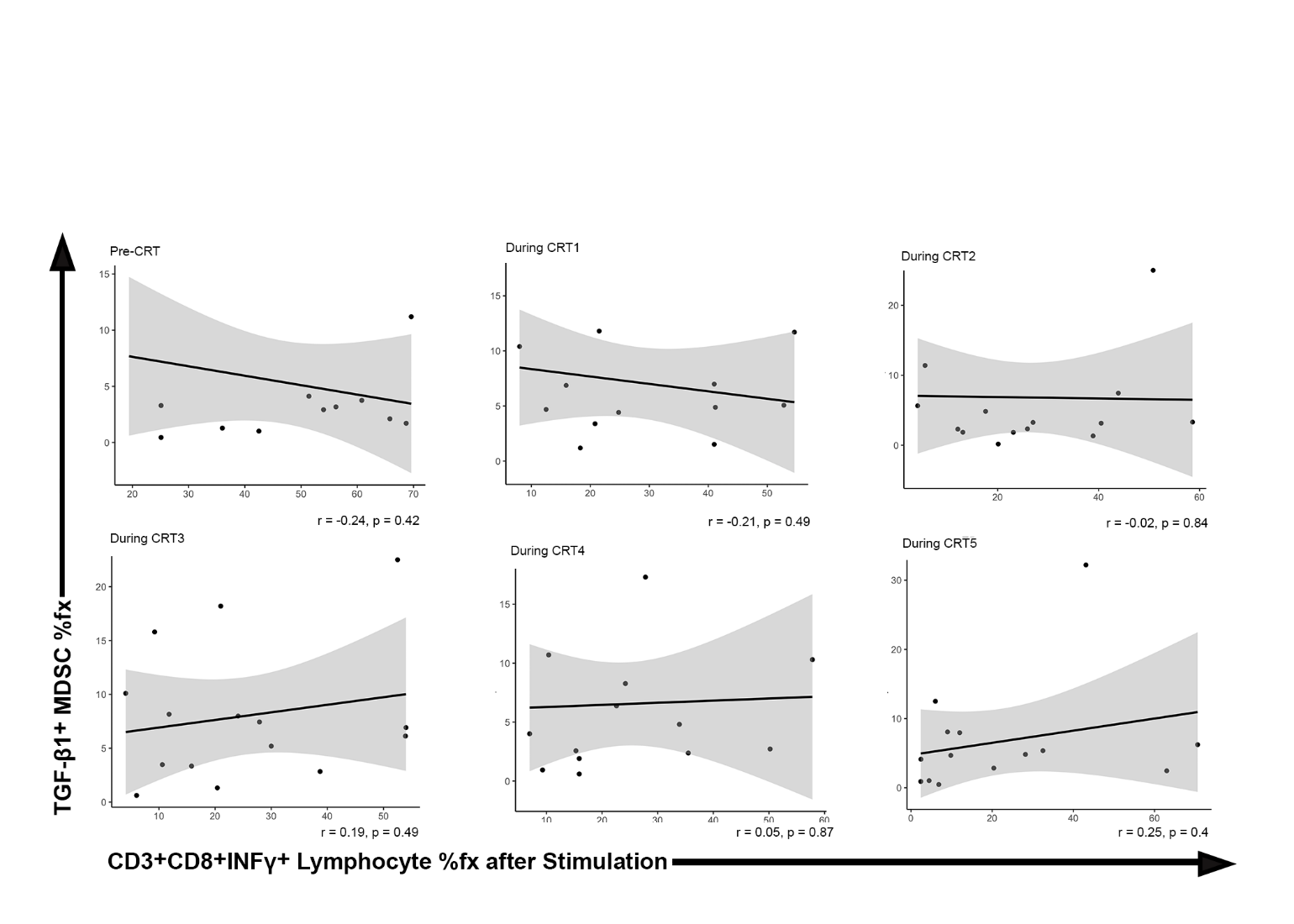


**Supplementary Figure 9a.** Correlation between TGFβ^+^ MDSC %fx by Flow Cytometry and CD3^+^CD8^+^IFNγ^+^ Lymphocyte %fx after Stimulation.


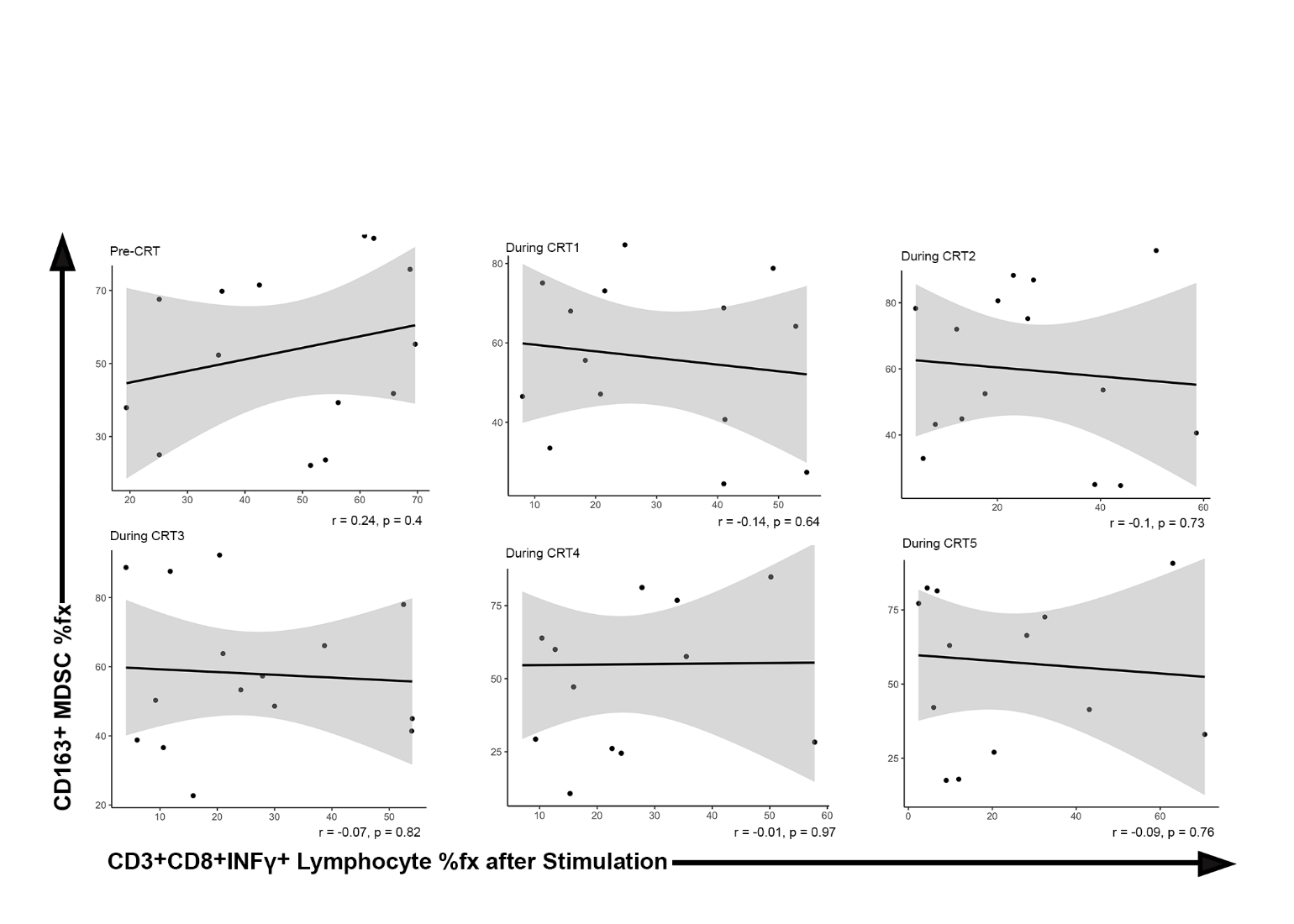


**Supplementary Figure 9b.** Correlation between CD163^+^ MDSC %fx by Flow Cytometry and CD3^+^CD8^+^IFNγ^+^ Lymphocyte %fx after Stimulation.


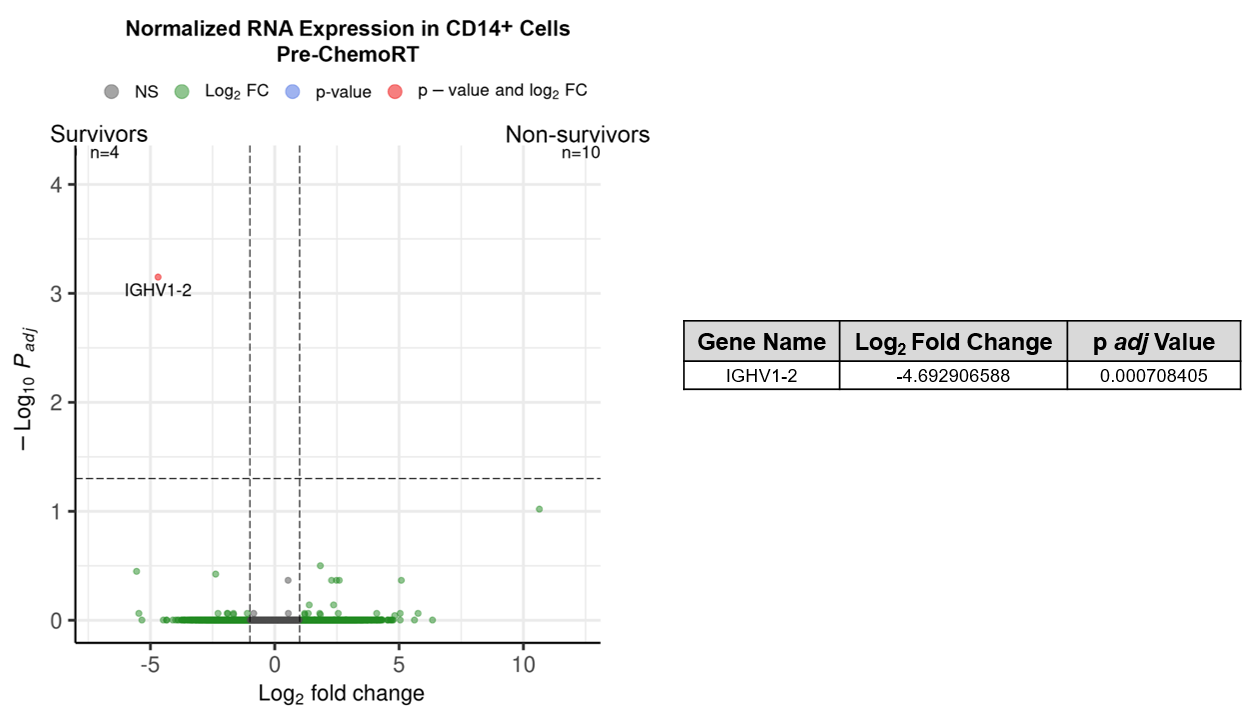


**Supplementary Figure 10a.** RNA Expression by CD14^+^ Cells by Bulk RNAseq Pre-ChemoRT.


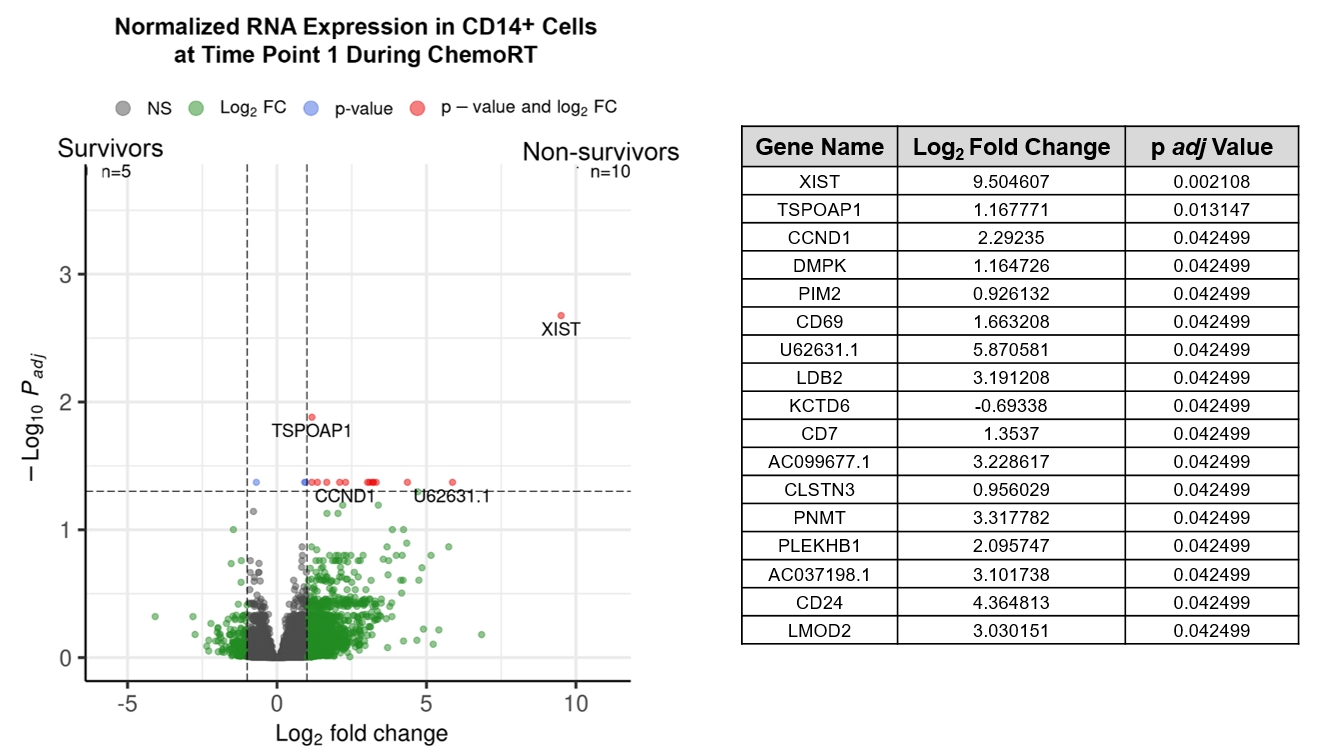


**Supplementary Figure 10b.** RNA Expression by CD14^+^ Cells by Bulk RNAseq at During CRT 1.


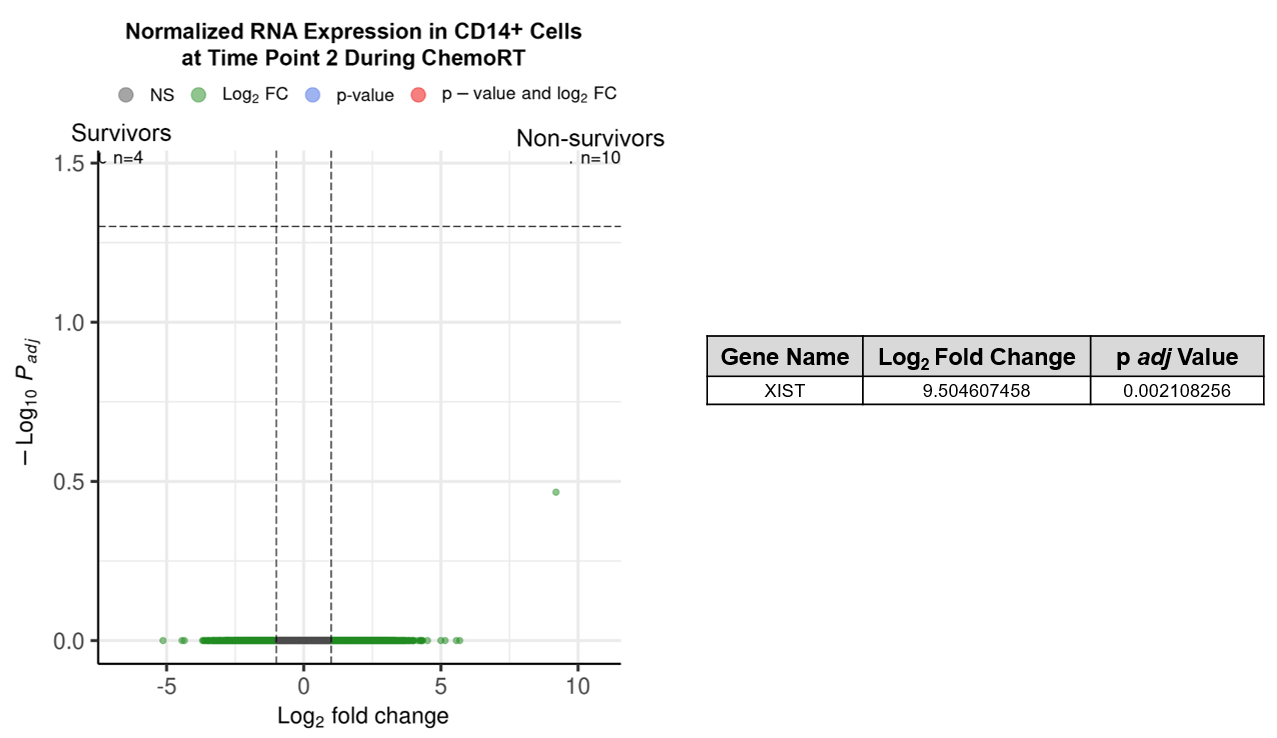


**Supplementary Figure 10c.** RNA Expression by CD14^+^ Cells by Bulk RNAseq at During CRT 2.


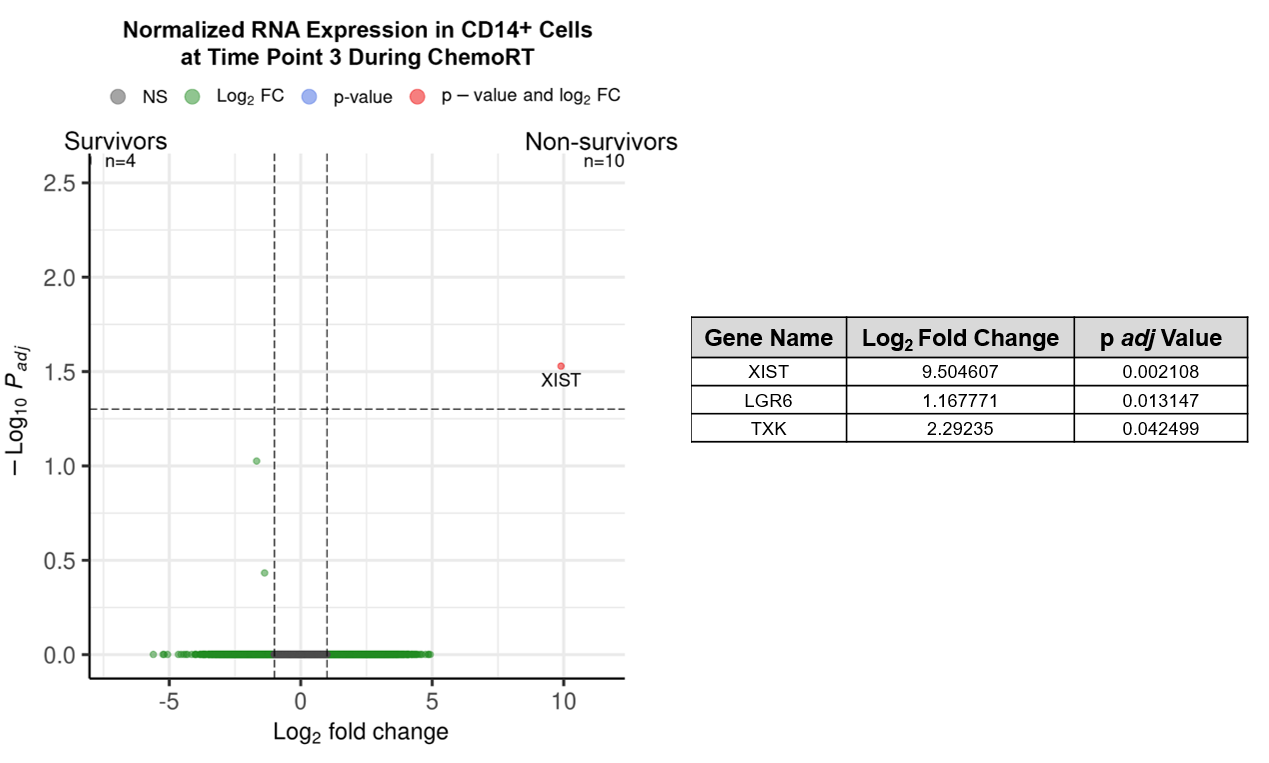


**Supplementary Figure 10d.** RNA Expression by CD14^+^ Cells by Bulk RNAseq at During CRT 3.


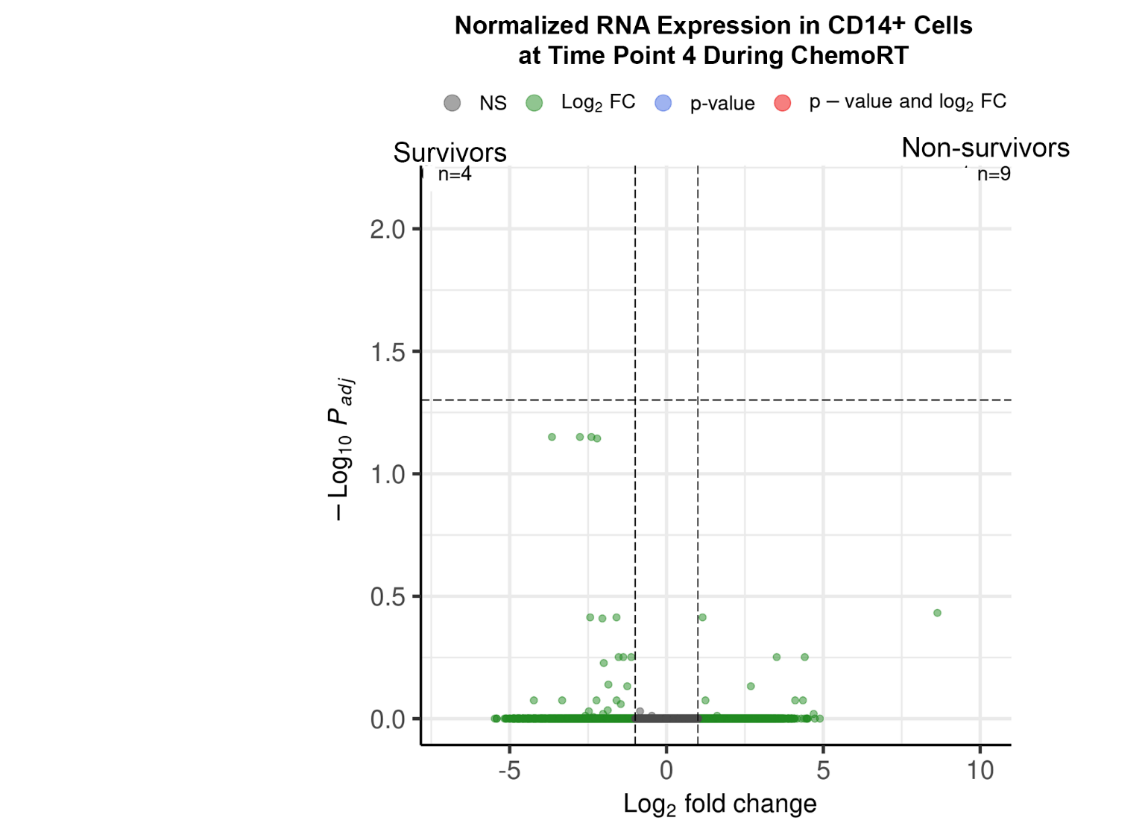


**Supplementary Figure 10e.** RNA Expression by CD14^+^ Cells by Bulk RNAseq at During CRT 4.


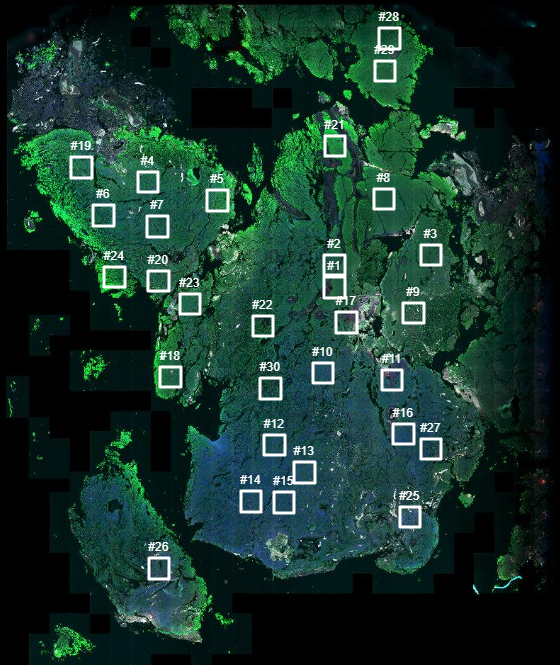


**Supplementary Figure 11a.** Digital Spatial Profiling (DSP) Example of GBM Tumor Tissue: Example 2, Survivor.


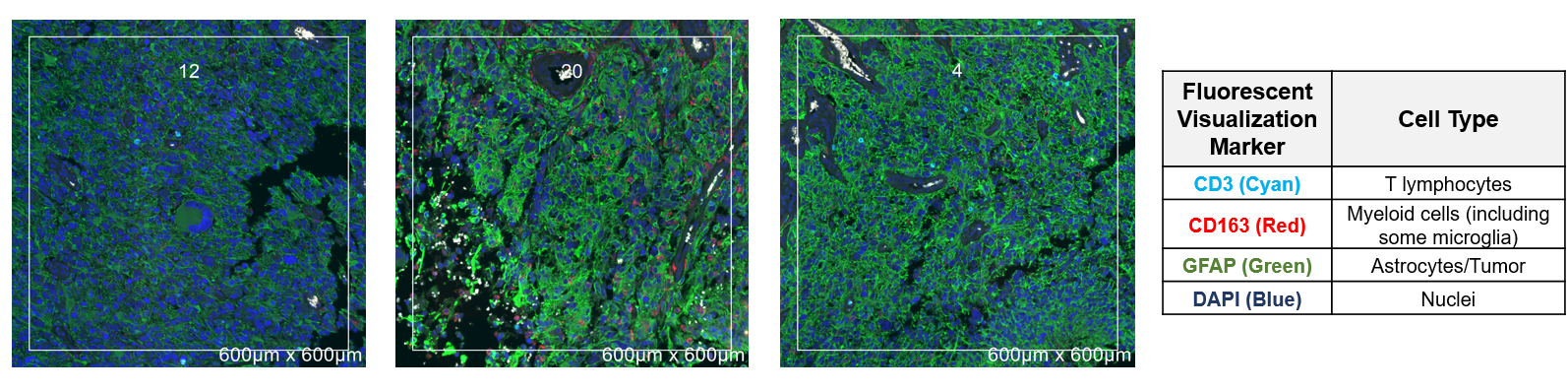
**Supplementary Figure 11b.** Example Regions of Interest (ROIs) of DSP: Example 2, Survivor. Magnified views (600 x 600 μm) of example regions of interest (ROIs).Visualization markers were used to identify ROIs by immunofluorescence.

**Supplementary Figure 12a.** Normalized CD163 Protein Expression by DSP ROI in GBM Tissue from Example 1, Non-survivor.

**Supplementary Figure 12b.** Normalized CD163 Protein Expression by DSP ROI in GBM Tissue from Example 2, Survivor.

|  | **Company** | **Catalogue No.** | **Clone** |
| --- | --- | --- | --- |
| CD14 | BioLegend | 367120 | 63D3 |
| CD3 | BioLegend | 300326 | HIT3a |
| CD4 | BioLegend | 344622 | SK3 |
| CD8 | BD | 564116 | SK1 |
| CD16 | BD | 563689 | 3G8 |
| CD33 | BD | 562492 | WM53 |
| CD163 | BD | 563888 | GHI/61 |
| CD80 | BD | 565157 | L307.4 |
| CD86 | BioLegend | 305420 | IT2.2 |
| HLA-DR | BioLegend | 307640 | L243 |
| CSF1R | BioLegend | 347310 | 9-4D2-1E4 |
| PD-1 | BD | 565936 | EH12.1 |
| PD-L1 | BD | 557924 | MIH1 |
| LAG3 | BioLegend | 369204 | 7H2C65 |
| TIM-3 | BD | 565563 | 7D3 |
| CTLA-4 | BioLegend | 369628 | BNI3 |
| TIGIT | BioLegend | 372716 | A15153G |
| VSIG4 | eBioscience | 17-5757-42 | JAV4 |
| BTLA4 | BioLegend | 344506 | MIH26 |
| IL-10 | BD | 564049 | JES3-9D7 |
| IFNγ | BioLegend | 502538 | 4S.B3 |
| M-CSF | R&D Systems | IC2161G | 26786 |
| IL-34 | R&D Systems | IC5265P | 578416 |
| TGFβ1 | BioLegend | 300006 | S20006A |
| FoxP3 | BioLegend | 563955 | 236A/E7 |

**Supplementary Table 1.** Flow Cytometry Reagent Details.
